# Reconstruction of the pelvic girdle and hindlimb musculature of the early tetanurans Piatnitzkysauridae (Theropoda, Megalosauroidea)

**DOI:** 10.1111/joa.13983

**Published:** 2023-12-01

**Authors:** Mauro B. S. Lacerda, Jonathas S. Bittencourt, John R. Hutchinson

**Affiliations:** ^1^ Structure and Motion Laboratory, Department of Comparative Biomedical Sciences The Royal Veterinary College Hatfield UK; ^2^ Pós‐Graduação em Zoologia Instituto de Ciências Biológicas, Universidade Federal de Minas Gerais Belo Horizonte Brazil; ^3^ Departamento de Geologia Instituto de Geociências, Universidade Federal de Minas Gerais Belo Horizonte Brazil

**Keywords:** Dinosauria, extant phylogenetic bracket, functional morphology, Jurassic, soft‐tissue

## Abstract

Piatnitzkysauridae were Jurassic theropods that represented the earliest diverging branch of Megalosauroidea, being one of the earliest lineages to have evolved moderate body size. This clade's typical body size and some unusual anatomical features raise questions about locomotor function and specializations to aid in body support; and other palaeobiological issues. Biomechanical models and simulations can illuminate how extinct animals may have moved, but require anatomical data as inputs. With a phylogenetic context, osteological evidence, and neontological data on anatomy, it is possible to infer the musculature of extinct taxa. Here, we reconstructed the hindlimb musculature of Piatnitzkysauridae (*Condorraptor*, *Marshosaurus*, and *Piatnitzkysaurus*). We chose this clade for future usage in biomechanics, for comparisons with myological reconstructions of other theropods, and for the resulting evolutionary implications of our reconstructions; differential preservation affects these inferences, so we discuss these issues as well. We considered 32 muscles in total: for *Piatnitzkysaurus*, the attachments of 29 muscles could be inferred based on the osteological correlates; meanwhile, in *Condorraptor* and *Marshosaurus*, we respectively inferred 21 and 12 muscles. We found great anatomical similarity within Piatnitzkysauridae, but differences such as the origin of *M*. *ambiens* and size of *M*. *caudofemoralis brevis* are present. Similarities were evident with Aves, such as the division of the *M*. *iliofemoralis externus* and *M*. *iliotrochantericus caudalis* and a broad depression for the *M*. *gastrocnemius pars medialis* origin on the cnemial crest. Nevertheless, we infer plesiomorphic features such as the origins of *M*. *puboischiofemoralis internus 1* around the “cuppedicus” fossa and *M*. *ischiotrochantericus* medially on the ischium. As the first attempt to reconstruct muscles in early tetanurans, our study allows a more complete understanding of myological evolution in theropod pelvic appendages.

## Graphical abstract

Piatnitzkysauridae were Jurassic theropods that represented one of the earliest lineages to have evolved moderate body size. Here, we reconstructed the hindlimb musculature of this clade, allowing a more complete understanding of myological evolution in theropod pelvic appendages.

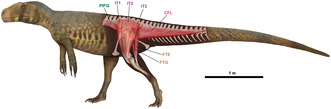

## 1 INTRODUCTION

Piatnitzkysauridae is a clade of medium‐sized (~4 to 6 m long; ~200 kg body mass) tetanuran theropods within Megalosauroidea (sensu Carrano et al., 2012), known from the Jurassic of South America and North America (Bonaparte, 1979; Madsen, 1976; Rauhut, 2005). However, in alternative phylogenies (Rauhut & Pol, 2019; Schade et al., 2023), Piatnitzkysauridae is considered to be an early divergent clade of Allosauroidea. Evolutionary implications related to the locomotor skeletal system of Tetanurae based on the alternative position of Piatnitzkysauridae were discussed by Lacerda et al. (2023).

Currently, at least three taxa constitute Piatnitzkysauridae: *Piatnitzkysaurus floresi* Bonaparte, 1979 and *Condorraptor currumili* Rauhut, 2005, from the late Toarcian to late Bajocian (Middle Jurassic) assemblages of the Cañadón Asfalto Formation in Patagonia, Argentina (Cúneo et al., 2013; Olivera et al., 2015); and *Marshosaurus bicentesimus* Madsen, 1976, from the Kimmeridgian (Upper Jurassic) assemblages of the Morrison Formation in the United States (Utah; possibly Colorado). A phylogenetic definition of the clade was presented by Carrano et al. (2012) as all megalosauroid theropods that are more closely related to *Piatnitzkysaurus* than to *Spinosaurus* or *Megalosaurus*. However, in some phylogenetic studies/hypotheses (e.g., Benson, 2010; Dai et al., 2020; Rauhut et al., 2016), the poorly preserved Middle Jurassic taxon *Xuanhanosaurus* from China falls within piatnitzkysaurids as an early diverging species. However, this taxon also has been recovered as an early tetanuran (Holtz et al., 2004) or an allosauroid (Carrano et al., 2012); and, therefore, is considered a “wildcard” taxon (Carrano et al., 2012).

*Condorraptor* and *Piatnitzkysaurus* are taxa of great importance, both geographically and temporally, as they are some of the few known Middle Jurassic theropods with a relatively well‐preserved skeleton, especially considering the fossil record from South America (Bonaparte, 1979; Carrano et al., 2012; Rauhut, 2003, 2004, 2005, 2007). They also provide important phylogenetic clues about the evolution of early theropod dinosaurs (Carrano et al., 2012; Lacerda, 2023; Rauhut, 2003). Concerning the skull, the North American taxon *Marshosaurus* is better known than both *Condorraptor* and *Piatnitzkysaurus* (Carrano et al., 2012; Chure et al., 1997; Madsen, 1976), also preserving a rare case of osteopathological evidence (Chure et al., 1997). Additional skeletal elements (e.g., Chure et al., 1997) are as yet undescribed. The two Argentinean taxa are also known from decent skeletal material: both skeletons of *Piatnitzkysaurus* are relatively well‐preserved including a sizeable portion of the appendicular skeleton and braincase, for example; and *Condorraptor*, although more fragmentary, has numerous postcranial elements (e.g., Bonaparte, 1986; Novas, 2009; Paulina‐Carabajal, 2015; Rauhut, 2004, 2005, 2007).

Piatnitzkysauridae is a key clade for understanding the evolution of tetanuran theropods because they are the earliest and oldest known members of this clade (Carrano et al., 2012; Rauhut et al., 2016). The main distinctions between *Piatnitzkysaurus*, *Condorraptor* and *Marshosaurus* are based on characters present in the dentaries, axial skeleton, and tibia (Bonaparte, 1986; Carrano et al., 2012; Madsen, 1976; Rauhut, 2005); however, additional dissimilarities in pelvic bones and zeugopodial elements are also recognizable (Lacerda et al., 2023). Furthermore, the Middle Jurassic was an important time for the diversification of tetanuran theropods, which soon populated all continents, although these main evolutionary patterns remain poorly known (e.g., Rauhut, 2004, 2005; Sereno, 1999).

Piatnitzkysaurid species can be diagnosed, for example, by the following morphological features: (1) short or absent anterior maxillary ramus, (2) presence of two parallel rows of foramina on the maxilla, (3) vertically striated paradental plates, and (4) anteriorly inclined neural spines of the posterior dorsal vertebrae (further details in Carrano et al., 2012). The first cladistic studies that phylogenetically positioned and characterized these species as a clade were Benson (2010) and Carrano et al. (2012), who included the piatnitzkysaurids within the clade Megalosauroidea, differing from other approaches. Historical classifications generally had assigned *Marshosaurus* and *Piatnitzkysaurus* as members of allosaurids or megalosaurids (e.g., Bonaparte, 1979, 1986; Russell, 1984; for a summary see Carrano et al., 2012).

*Marshosaurus* and *Piatnitzkysaurus* are known from skeletons of adult individuals (Bonaparte, 1986; Madsen, 1976), whereas *Condorraptor* is known from a probably subadult specimen (Rauhut, 2005). The estimated typical body length of the three species is 4.5 m, with a body mass of about 200 kg for *Marshosaurus* and *Condorraptor*; whereas the body mass of *Piatnitzkysaurus* was estimated as 275 kg (Paul, 1988, 2016). Hendrickx et al. (2015) estimated longer body lengths, between 5 and 6 m, and Foster (2020) estimated a slightly greater body mass for *Marshosaurus* (250 kg). Nevertheless, one estimate of body mass, which differs from the others, as it is based on femoral morphometrics, suggests that the Argentinean taxa *Condorraptor* and *Piatnitzkysaurus* could have reached ~360 and 750 kg in mass, respectively; exemplifying the origin of medium‐sized tetanurans during the Jurassic and thus suggesting an increase in theropod macropredatory habits (Benson et al., 2014).

## 2 MUSCLE RECONSTRUCTION IN EXTINCT VERTEBRATES

Reconstruction of muscles and estimation of their architecture and functions is an important approach in palaeobiology (e.g., Bates & Falkingham, 2018; Bishop et al., 2021; Cuff, Demuth et al., 2023; Witmer, 1995). Even with intrinsic limitations to these reconstructions for fossil organisms, biomechanical models and simulations; and other useful methods; have been developed with the aid of computational techniques (e.g., Hutchinson, 2012). Advances in morphofunctional and ecomorphological studies in extinct vertebrates, together with advances in evolutionary biomechanics applied to locomotion, for example, are essential for understanding broader macroevolutionary aspects such as paleoecology and potential selective pressures (e.g., Jones et al., 2021).

Over a century of studies has focused on the variations in pelvic and hindlimb functional morphology in extinct and extant archosaur species and its implications for muscle architecture and locomotor biomechanics. These studies have provided broad datasets, a solid background, and general inferences that have led to a greater understanding of comparative myology and biomechanical evolution of locomotion at different levels (e.g., Allen et al., 2021; Bates, Benson, & Falkingham, 2012; Bates, Maidment, et al., 2012; Bishop, Hocknull, Clemente, Hutchinson, Barret, et al., 2018; Bishop, Hocknull, Clemente, Hutchinson, Farke, et al., 2018; Bishop, Cuff, & Hutchinson, 2021; Carrano & Hutchinson, 2002; Cerroni et al., 2022; Costa et al., 2014; Cuff, Demuth, et al., 2023; Cuff, Wiseman, et al., 2023; Farlow et al., 1995, 2000; Gatesy, 1990; Gatesy & Middleton, 1997; Gregory & Camp, 1918; Grillo & Azevedo, 2011; Hutchinson, 2001a, 2004a, 2004b, 2012; Hutchinson et al., 2005; Hutchinson & Allen, 2009; Hutchinson & Garcia, 2002; Langer, 2003; Liparini & Schultz, 2013; Maidment & Barrett, 2011; Mallison, 2010; Piechowski & Tałanda, 2020; Romer, 1923a, 1923b, 1923c, 1927; Rowe, 1986; Russell, 1972; Schachner et al., 2011; Smith, 2021, 2023; Tarsitano, 1983; Zinoviev, 2011).

However, how can these reconstructions be accurately performed for extinct vertebrates? In general, soft tissues (e.g., muscles/tendons) are not normally preserved in fossils. Yet there are rare exceptions where favourable geochemical conditions occurred during fossil diagenesis, providing rare preservation. These exceptions include muscle fibres or tendons, partial musculature and internal organs (e.g., Dal Sasso & Signore, 1998; Kellner, 1996; Surmik et al., 2023), as well as integumentary structures (e.g., Barbi et al., 2019; Bell et al., 2022) in dinosaurs. With few exceptions, almost all vertebrate fossils consist of some degree of biomineralization (e.g., bones, teeth, ossified ligaments/tendons). Nonetheless, some bony structures (e.g., muscle origins/insertions) leave discernible anatomical traces on fossils; thus, this muscle–bone interface allows reconstruction of unpreserved locomotor musculature based on a reliable osteological set of features (e.g., Bishop et al., 2021; Carrano & Hutchinson, 2002; Dilkes, 2000; Gatesy, 1990; Grillo & Azevedo, 2011; Hutchinson, 2001a, 2001b; Maidment & Barrett, 2011; Rhodes et al., 2021; Romer, 1923b, 1923c, 1927; Smith, 2021).

A methodology that has been widely used in recent decades is the Extant Phylogenetic Bracket (EPB), formalized by Witmer (1995). The EPB is based on the phylogenetic relationships of the extinct clade under study, with at least two evolutionarily outgroups having extant representatives. The EPB method represents a rigorously explicit method that aims to minimize speculations in muscle reconstruction, allowing tissue reconstruction to be performed and then judged through inference levels (see Section 4 below). Additionally, the inclusion of fossil taxa facilitates interpretations about muscular homology and evolution, because extinct relatives of the study taxon may present evidence for transitional character states or even novel states; either of these being absent in extant taxa (Bishop et al., 2021; Dilkes, 2000; Hutchinson, 2001a, 2001b, 2002; Maidment & Barrett, 2011).

The EPB has been particularly popular for studying locomotor form and function in archosaurs (e.g., Bates, Maidment, et al., 2012; Bishop, Hocknull, Clemente, Hutchinson, Barret, et al., 2018; Bishop, Hocknull, Clemente, Hutchinson, Farke, et al., 2018; Carrano & Hutchinson, 2002; Grillo & Azevedo, 2011; Hutchinson, 2001a, 2001b; Langer, 2003; Liparini & Schultz, 2013; Otero, 2018; Otero et al., 2017; Rhodes et al., 2021; Smith, 2021). Because many extinct organisms do not have analogous extant taxa (Bishop et al., 2021; Costa et al., 2014), muscle reconstructions can provide different *a posteriori* interpretations and revisions of previously raised hypotheses (e.g., for *Tyrannosaurus rex*, pelvic muscle reconstructions by Romer, 1923b vs. Carrano & Hutchinson, 2002; and running abilities by Paul, 1988 vs. Hutchinson & Garcia, 2002).

## 3 WHY STUDY MUSCULATURE IN NON‐AVIAN THEROPODS?

Hutchinson and Allen (2009) listed at least four questions considered fundamental for the understanding of macroevolution and morphofunctional adaptations that support and motivate researchers to reconstruct the musculature and locomotor aspects in theropod dinosaurs: (1) how did the bipedal stance and gait of birds evolve? (2) what myological/locomotor traits are novel for birds? (3) how far down the phylogenetic tree is it possible to trace ancestral traits in theropods (or other archosaurs), and what are the plesiomorphic traits? and (4) how did novelties such as bipedalism and flight arise and/or were modified, or even how did the performance of terrestrial/aerial locomotion change over evolutionary time?

To answer some of these questions, there are growing efforts in the study of musculature, especially the locomotor apparatus in dinosaurs (e.g., Dilkes, 2000; Langer, 2003; Maidment & Barrett, 2011; Mallison, 2010). Considering Theropoda, among the myological reconstructions and modelling carried out so far, in addition to pioneering work (e.g., Farlow et al., 2000; Gatesy, 1990; Gatesy & Middleton, 1997; Romer, 1923a, 1923b, 1927; Russell, 1972; Tarsitano, 1983), the results presented for one of the earliest theropods, the herrerasaurid *Staurikosaurus* (Grillo & Azevedo, 2011), are worth highlighting, in addition to the reconstruction of the coelophysoid *Coelophysis* (Bishop et al., 2021). Regarding ceratosaurs, Persons IV and Currie (2011) did not fully reconstruct the locomotor musculature, but explored the caudal musculature in the abelisauroid *Carnotaurus*. Cerroni et al. (2022) explored the pelvic and hindlimb musculature of the abelisaurid *Skorpiovenator*. Concerning early tetanuran theropods, the only efforts to date relate to the allosauroids *Allosaurus* and *Acrocanthosaurus*, not only on the basis of musculature (e.g., Cau & Serventi, 2017), but also body mass estimation and biomechanical analysis (Bates, Benson, & Falkingham, 2012). Other muscle reconstructions generally have been carried out for lineages that are more closely related to Aves, with great effort spent on Coelurosauria; for example, the tyrannosauroid *Tyrannosaurus* (Carrano & Hutchinson, 2002), Ornithomimidae (Russell, 1972), Megaraptoridae (White et al., 2016), unenlagiids (Motta et al., 2018), alvarezsaurids (Meso et al., 2021), and maniraptoran species (Hutchinson et al., 2008; Rhodes et al., 2021; Smith, 2021, 2023).

In addition to the studies cited above, there is an ongoing effort to understand the main evolutionary features related to bipedalism in theropod dinosaurs (e.g., Allen et al., 2021; Bishop, Hocknull, Clemente, Hutchinson, Barret, et al., 2018; Bishop, Hocknull, Clemente, Hutchinson, Farke, et al., 2018; Cuff, Demuth, et al., 2023). However, the earliest tetanuran clades studied generally include only allosauroids; whereas there have not been detailed studies of Megalosauroidea, the earliest‐diverging branch of tetanuran evolution. Recently, Lacerda et al. (2023) mapped the evolution and reconstructed the ancestral states of the morphological characters of the pelvic appendage in Megalosauroidea, characterizing potential variations related to muscle attachments; and tested whether different homoplastic signals in different regions of the locomotor system are present in theropods. That study provides a stronger basis for the muscle reconstruction performed here (see below).

Although piatnitzkysaurids are important representatives for understanding theropod evolution (Carrano et al., 2012; Rauhut, 2003), as well as tetanuran diversity and the acquisition of larger body size in terms of locomotor function and body support, little is known about these paleobiological issues (Lacerda et al., 2023). Our aim here is to begin addressing these deficiencies by reconstructing the hindlimb muscles (origins and insertions) of the three piatnitzkysaurid species (*Condorraptor*, *Marshosaurus*, and *Piatnitzkysaurus*), and to compare our findings with the myological reconstructions of other extinct and extant archosaurs. We chose these taxa not only for: (1) future usage in biomechanical models, and (2) comparisons with existing myological reconstructions of other theropods and resulting evolutionary implications, but also (3) addressing how similar their musculature might have been, (4) determining if any show unusual apomorphies, and (5) assessing how differential taphonomic preservation affects these inferences. We considered a total of 32 muscles, focusing on the major muscles (not the many, small, complex pedal muscles).

*Institutional abbreviations*. MACN, Museo Argentino de Ciencias Naturales “Bernardino Rivadavia,” Buenos Aires, Argentina. MPEF, Museo Paleontológico Egidio Feruglio, Trelew, Argentina. PVL, Fundación “Miguel Lillo,” San Miguel de Tucumán, Argentina. UMNH, Natural History Museum of Utah, Utah, United States.

## 4 MATERIALS AND METHODS

### 4.1 Species and specimens

For *Piatnitzkysaurus*, we personally examined the holotype PVL 4073, housed at Fundación Miguel Lillo (Universidad Nacional de Tucumán, Argentina), and the partial skeleton (hypodigm MACN‐Pv‐CH 895), housed at Museo Argentino de Ciencias Naturales “Bernardino Rivadavia” (Argentina). For *Condorraptor*, we directly inspected the holotype MPEF‐PV 1672, as well as the hypodigm specimens (MPEF‐PV 1676–1683, MPEF‐PV 1686–1688, MPEF‐PV 1690–1693, MPEF‐PV 1696–1697, MPEF‐PV 1700–1702, MPEF‐PV 1704–1705), deposited in the Museo Paleontológico Egidio Feruglio (Argentina). For *Marshosaurus*, although one of us (JRH) personally examined known specimens (UMNH VP 6372 [=UUVP 1845], UMNH VP 6374 [=UUVP 2742], UMNH VP 6380 [=UUVP 2878], UMNH VP 6384 [=UUVP 40–295], UMNH VP 6387 [=UUVP 4736]) deposited in the Natural History Museum of Utah (United States), the myological inferences presented here are based upon photographs and notes from those examinations, and the original description provided by Madsen (1976). More detailed information is focused only on the South American taxa, for which we have the best image quality and which have been studied recently by one of us (MBSL).

### 4.2 Myological reconstruction, homology and character mapping

**FIGURE 1:**
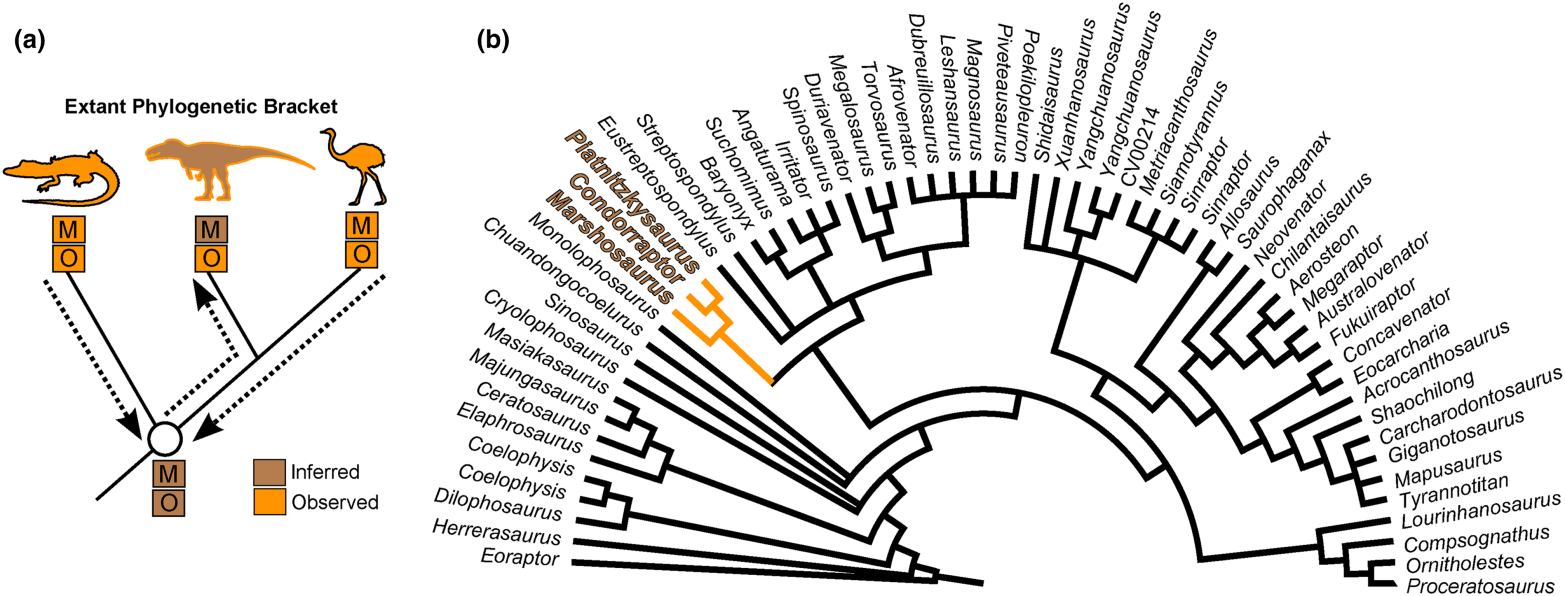
(a) Simplified example of the Extant Phylogenetic Bracket (EPB) application in Theropoda. (b), Theropod phylogeny (up to Coelurosauria on the right side of the phylogeny) highlighting the phylogenetic position of Piatnitzkysauridae. (a), adapted from Grillo & Azevedo, 2011; (b), adapted from Carrano et al., 2012. M, muscle; O, osteological correlate. Silhouettes are from phylopic.org; see Acknowledgements.

We used the EPB method (Witmer, 1995) for our reconstructions (Figure 1a). Three levels of inference are established by EPB to characterize the confidence in reconstructing a particular soft tissue for an extinct species: (I) represents an unequivocal structure of a particular feature, that is, when the two (or more) extant taxa have the homologous soft tissue and its osteological correlate; (II) represents an equivocal reconstruction, when the ancestral condition for two or more taxa is ambiguous, such as the presence of a particular soft tissue and the osteological correlate only in one of the extant taxa; (III) represents an unequivocal absence of a particular feature, that is, when the ancestral condition favoured by the EPB involves not having the soft tissue and its osteological evidence (i.e., inferring an absent feature; with no or contrary evidence). In addition, if soft tissue inferences lack conclusive data from their osteological correlates, they are qualified as level I′, II', and III' inferences (Witmer, 1995). Using the EPB, our comparisons mainly were based on Crocodylia and Aves, but not restricted to these groups; Lepidosauria and Testudines were also considered (Bishop et al., 2021; Hutchinson, 2002). The pelvic and thigh musculature of extant taxa was evaluated from the following literature on Crocodylia (e.g., Hattori & Tsuihiji, 2021; Otero et al., 2010; Romer, 1923a; Suzuki et al., 2011; Wilhite, 2023), Avialae (e.g., Clifton et al., 2018; Hattori & Tsuihiji, 2021; Hudson et al., 1959; Meso et al., 2021; Patak & Baldwin, 1998; Picasso, 2010; Romer, 1923c; Rowe, 1986; Suzuki et al., 2014), and other Tetrapoda/Reptilia (e.g., Dick & Clemente, 2016; Gregory & Camp, 1918; Hattori & Tsuihiji, 2021; Romer, 1942). Dissection of one *Crocodylus niloticus* and one *Numida meleagris* specimen during this study further enhanced our musculoskeletal comparisons and delineations of the locomotory muscle positioning.

The phylogenetic framework adopted here was provided by Carrano et al. (2012), where Piatnitzkysauridae is an early Megalosauroidea clade composed of *Marshosaurus* as the earliest piatnitzkysaurid taxon to diverge, being sister taxon of a subclade composed of *Piatnitzkysaurus* and *Condorraptor* (Figure 1b). However, see Rauhut and Pol (2019) and Schade et al. (2023) for an alternative hypothesis; as discussed above (see also Lacerda et al., 2023).

The nomenclature and homology of the musculoskeletal system here follow the propositions of Hutchinson and Gatesy (2000), Hutchinson (2001a, 2001b, 2002), Carrano and Hutchinson (2002), and Hattori & Tsuihiji, 2021 (adaptations summarized in Table 1), which built on earlier work by Romer (1923a, 1942) and Rowe (1986). The nomenclature of Baumel and Witmer (1993) is followed in the descriptions of osteological correlates and muscle scars.

**TABLE 1:** Muscular homologies in extant archosaurs, considering the musculature of the pelvic girdle and hindlimb (modified from Carrano & Hutchinson, 2002). The EPB uses the state in each most recent common ancestor of Crocodylia and of Aves as its bracket, informed by further data from outgroups Lepidosauria and Testudines (not shown here; see Hutchinson, 2002).

Muscles (Crocodylia)	Muscles (Aves)
Dorsal group	
*Triceps femoris*	
*M. iliotibialis 1* (IT1)	*M. iliotibialis cranialis* (IC)
*Mm. iliotibialis 2, 3* (IT2, IT3)	*M. iliotibialis lateralis* (2 main parts) (IL)
*M. ambiens* (AMB)	*M. ambiens* (AMB)
*M. femorotibialis externus* (FMTE)	*M. femorotibialis lateralis* (FMTL)
*M. femorotibialis internus* (FMTI)	*M. femorotibialis intermedius* (FMTIM) & *M. femorotibialis medialis* (FMTM)
*M. iliofibularis* (ILFB)	*M. iliofibularis* (ILFB)
Deep dorsal	
*M. iliofemoralis* (IF)	*M. iliofemoralis externus* (IFE) & *M. iliotrochantericus caudalis* (ITC)
*M. puboischiofemoralis 1* (PIFI1)	*M. iliofemoralis internus* (IFI)
*M. puboischiofemoralis internus 2* (PIFI2)	*M. iliotrochantericus cranialis* (ITCR) & *M. iliotrochantericus medius* (ITM)
Ventral group	
*Flexor cruris*	
*M. puboischiotibialis* (PIT)	[absent]
*M. flexor tibialis internus 1* (FTI1)	[absent]
*M. flexor tibialis internus 2* (FTI2)	[absent]
*M. flexor tibialis internus 3* (FTI3)	*M. flexor cruris medialis* (FCM)
*M. flexor tibialis internus 4* (FTI4)	[absent]
*M. flexor tibialis externus* (FTE)	*M. flexor cruris lateralis pars pelvica* (FCLP and *accessoria* FCLA)
*Mm. adductores femores*	
*M. adductor femoris 1* (ADD1)	*M. puboischiofemoralis pars medialis* (PIFM)
*M. adductor femoris 2* (ADD2)	*M. puboischiofemoralis pars lateralis* (PIFL)
*Mm. puboischiofemorales externi*	
*M. puboischiofemoralis externus 1* (PIFE1)	*M. obturatorius lateralis* (OL)
*M. puboischiofemoralis externus 2* (PIFE2)	*M. obturatorius medialis* (OM)
*M. puboischiofemoralis externus 3* (PIFE3)	[absent]
*M. ischiotrochantericus* (ISTR)	*M. ischiofemoralis* (ISF)
*Mm. caudofemorales*	
*M. caudofemoralis brevis* (CFB)	*M. caudofemoralis pars pelvica* (CFP)
*M. caudofemoralis longus* (CFL)	*M. caudofemoralis pars caudalis* (CFC)
Digital extensor group	
*M. extensor digitorum longus* (EDL)	*M. extensor digitorum longus* (EDL)
*M. extensor digitorum brevis* (EDB)	[absent]
*M. extensor hallucis longus* (EHL)	*M. extensor hallucis longus* (EHL)
*M. tibialis anterior* (TA)	*M. tibialis cranialis* (TC)
*Mm. gastrocnemii*	
*M. gastrocnemius externus* (GE)	*M. gastrocnemius pars lateralis* (GL) *et intermedia* (GIM)
*M. gastrocnemius internus* (GI)	*M. gastrocnemius pars medialis* (GM)
Lower leg muscles	
*M. fibularis longus* (FL)	*M. fibularis longus* (FL)
*M. fibularis brevis* (FB)	*M. fibularis brevis* (FB)

*Piatnitzkysaurus* was scored for character states of 86 characters related to the pelvic musculature (character ranges 1–71, 78–88, and 97–100; see Appendix A), to replace the “basal Tetanurae” lineage (which previously was a rough composite of transitional character states from this and other lineages) from Hutchinson (2001a, 2001b, 2002) and Bishop et al. (2021) in a new taxon‐character matrix. As usual for the EPB, we used the maximum parsimony criterion for our reconstructions, similar to previous studies (e.g., Bishop et al., 2021; Molnar et al., 2018; Witmer, 1995). By doing so, we refine character scoring for early Tetanurae in general, which will be useful for future studies. We only scored *Piatnitzkysaurus*, as it has more osteological correlates preserved than the other taxa do, and consequently, a greater number of muscles could be inferred for this species (see Section 5). However, we sought to test if any muscles reconstructed differed in any details across the three taxa. To score and trace evolutionary changes in locomotor muscles, as well as to assess the most parsimonious states in our reconstructions, we used Mesquite software version 3.6 (Maddison & Maddison, 2015), using an informal composite “consensus” tree of Reptilia based on the recent phylogenetic framework used by Bishop et al. (2021) and references therein.

## 5 RESULTS AND DISCUSSION

### 5.1 Myological reconstruction

#### 5.1.1 Triceps femoris

*Mm. iliotibiales* (*IT1, IT2*, and *IT3*): In Aves and Crocodylia, the *Mm*. *iliotibiales* is a large and superficial sheet that generally is composed of three heads over the dorsal and anteroposterior rim of the ilium, superficially positioned in relation to the other pelvic and thigh muscles (Clifton et al., 2018; Hudson et al., 1959; Hutchinson, 2001b, 2002; Otero et al., 2010; Patak & Baldwin, 1998; Picasso, 2010; Romer, 1923a). In other Reptilia, the homologous muscle presents one or two weakly separated heads (Dick & Clemente, 2016; Hutchinson, 2002; Romer, 1942). The IT1–3 muscles attach to the dorsal rim of the ilium and are dorsally delimited by the *crista dorsolateralis ilii*, marking the border between the dorsal and lateral surfaces of the supraacetabular iliac blade (Baumel & Witmer, 1993).

**FIGURE 2:**
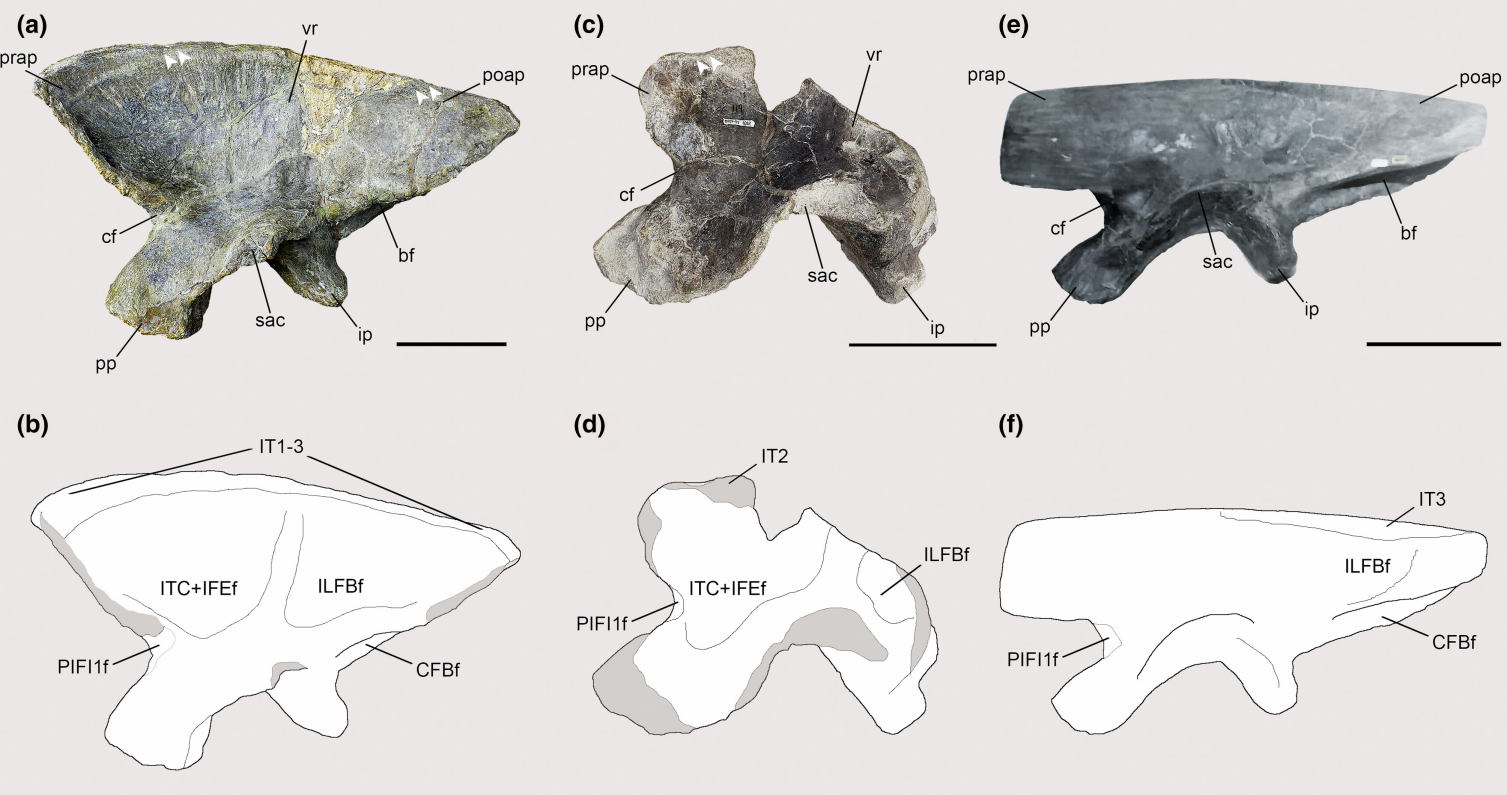
Osteological correlates observed in the ilia of Piatnitzkysauridae (left ilia, lateral view). (a, b) *Piatnitzkysaurus* (MACN‐Pv‐CH 895). (c, d) *Condorraptor* (MPEF‐PV 1687). (e, f) *Marshosaurus* (UMNH VP 6372). The *M*. *flexor tibialis externus* is not marked in the line drawings. Anatomical/muscular abbreviations: bf, brevis fossa; cf, “cuppedicus” fossa; CFBf, *M*. *caudofemoralis brevis* origin fossa; IFEf, *M*. *iliofemoralis externus* origin fossa; ILFBf, *M*. *iliofibularis* origin fossa; ip, ischiadic peduncle; IT1–3, *Mm*. *iliotibiales 1*–*3* origin scars; PIFI1, *M*. *puboischiofemoralis internus 1* origin fossa; poap, postacetabular process; pp, pubic peduncle; prap, preacetabular process; sac, supraacetabular crest; vr, vertical ridge. Arrows indicate potential subdivision of IT heads. Dark grey represents broken areas of bones. Scale bar = 100 mm.

**FIGURE 3:**
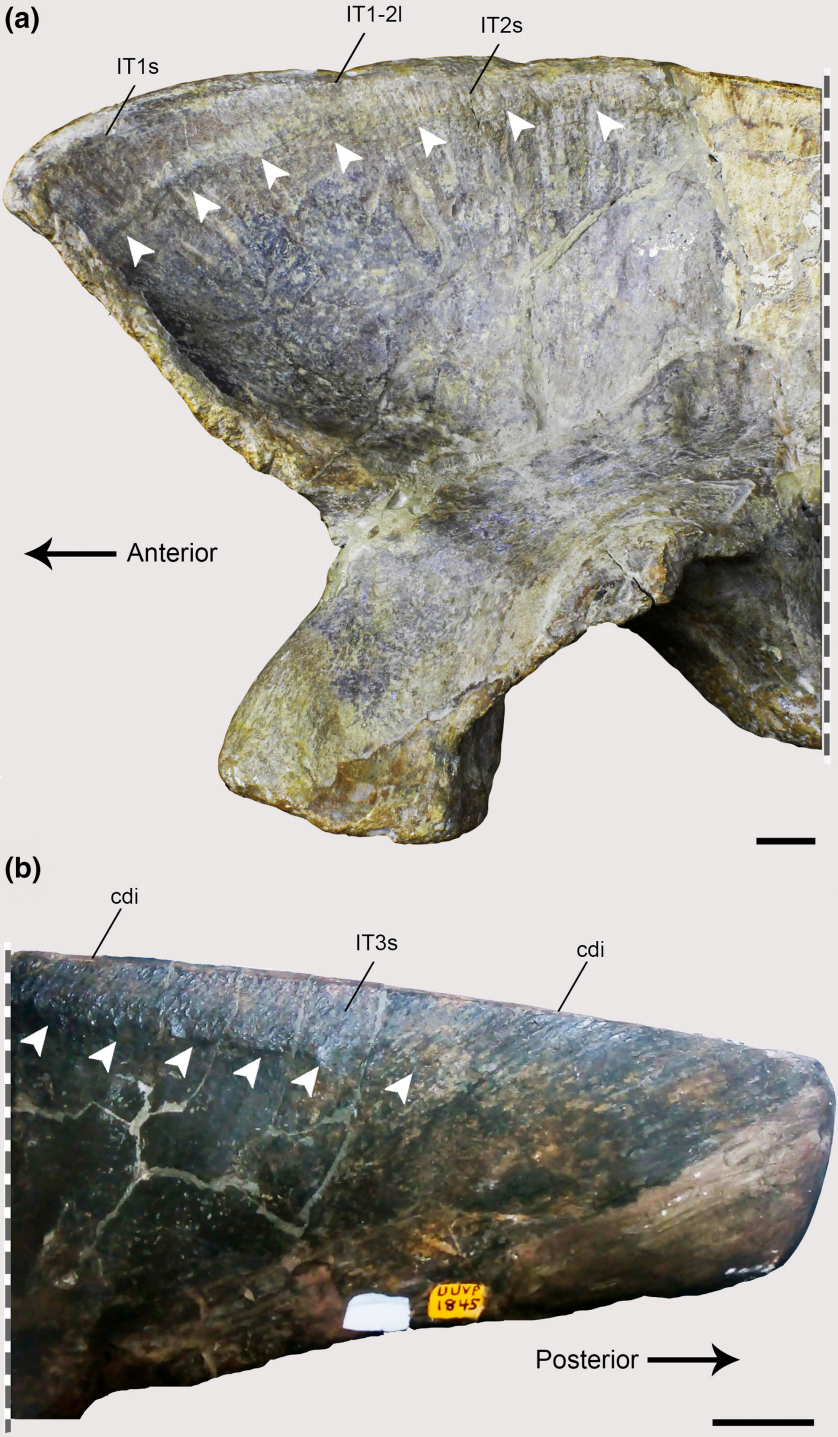
Osteological correlates of *M*. *iliotibiales* 1–3 observed on the ilia of Piatnitzkysauridae (left ilia, lateral view). (a), *Piatnitzkysaurus* (MACN‐Pv‐CH 895). (b), *Marshosaurus* (UMNH VP 6372). Anatomical/muscular abbreviations: cdi, *crista dorsolateralis ilii*; IT1–3s, *Mm*. *iliotibiales* scars; IT1–2l, *M*. *iliotibialis 1* and *2* limits. Arrows indicate muscle scars. Scale bar = 20 mm.

In the *Piatnitzkysaurus* ilium MACN‐Pv‐CH 895, the anteriormost margin of the preacetabular process is not preserved, so the anterior limits/extension of the IT1 are not possible to infer; however, a great part of the supraacetabular rim is preserved. On the anteriormost part of the preacetabular blade, an expanded area is evident. This area is posteriorly delimited by an invagination present over the dorsalmost part of the supraacetabular rim (Figure 2a,b). Furthermore, immediately ventral to the dorsal rim of the ilium, there is a rough osteological delimitation, which posteriorly becomes more dorsally positioned (Figure 3a). Because these osteological correlates are topologically compatible with the positions (and similar osteological correlates) noted in extant archosaurs (e.g., Carrano & Hutchinson, 2002; Hudson et al., 1959; Otero et al., 2010; Picasso, 2010; Romer, 1923a), the rough delimitation and the dorsal invagination seem to be the posterior edge of the IT1 (level I), as well as the anterior demarcation of the IT2 (Figures 2a and 3a). Concerning the IT2, we infer the anterior limits to be at the same position as the main axis of the pubic peduncle, on a dorsal invagination of the dorsal rim of the preacetabular blade (level I) (Figure 2a), as aforementioned. Although not clearly preserved, the posterior limits of this muscle head seem to be demarcated by a small protuberance on the dorsal postacetabular blade (Figure 2a), which is posterior to the posterior facet of the ischial peduncle. This protuberance also probably delimited the anterior origin of the IT3; the attachment area of the IT3 is on the posterior dorsal rim of the postacetabular blade of the ilium. A rough scar which becomes posteriorly large is on the ilium of MACN‐Pv‐CH 895, seeming to be dorsally delimited by the *crista dorsolateralis ilii*. This area of the IT3 is delimited by a faint osteological protuberance (level I). Most of the origination region of the IT1–3 muscles is not preserved in *Condorraptor*—the supraacetabular crest is highly damaged anterior and dorsal to the acetabulum in the only preserved ilium MPEF‐PV 1687 of this taxon (Figure 2c,d). Although fragmentary, this region has an osteological correlate indicating that the anterior boundaries of the IT2 origin were from an invagination preserved at the same axis of the pubic peduncle (level I). However, as a consequence of this poor preservation of the *Condorraptor* ilium, our reconstructions of the origins of IT1 and IT3, as well as the extent of IT2, are uncertain, although these origins should have been similar to those reconstructed for *Piatnitzkysaurus*. In the studied ilia of *Marshosaurus* (UMNH VP 6372 and UMNH VP 6374) and the holotype UMNH VP 6373 [=UUVP 2826] specimen (Madsen, 1976), the best‐preserved part is the postacetabular process of the ilium. Although the subdivisions of the IT heads are not as discernible as in *Piatnitzkysaurus*, the origin of the IT3 in both UMNH VP 6372 and UMNH VP 6373 is clearly discernible by several scars on the dorsal edge of the postacetabular blade and the presence of the *crista dorsolateralis ilii* (level I) (Figures 2e,f and 3b).

In Crocodylia, Aves and other Reptilia, those three heads of *Mm*. *iliotibiales* converge with *M*. *ambiens* and *Mm*. *femorotibiales* into at least one extensor tendon and fascial sheet, which inserts on the tibial cnemial crest or *crista cnemialis cranialis* (Baumel & Witmer, 1993) of the proximal metaphysis of the tibia (Dick & Clemente, 2016; Gregory & Camp, 1918; Hutchinson, 2002; Otero et al., 2010; Patak & Baldwin, 1998; Romer, 1923a, 1923b, 1942).

**FIGURE 4:**
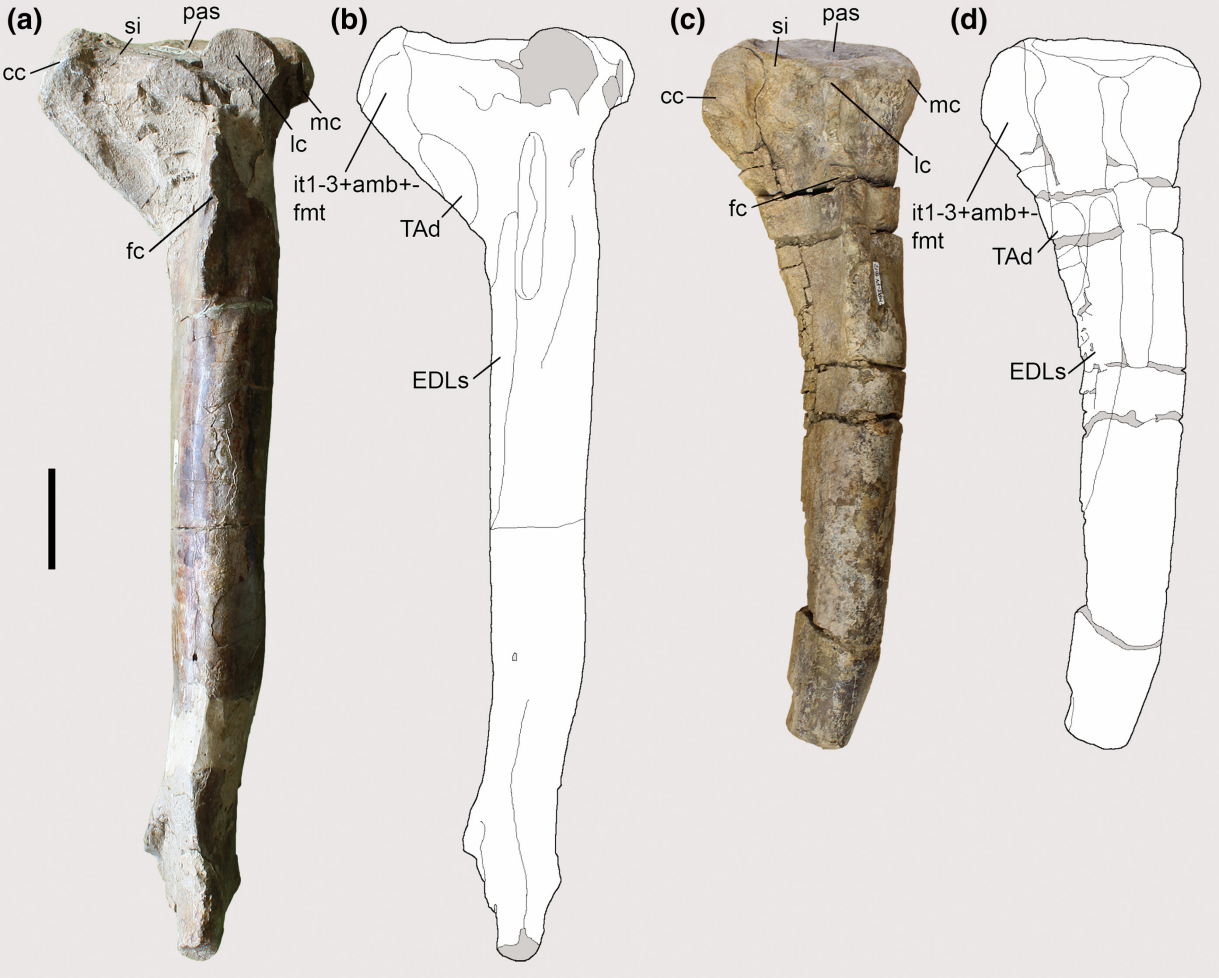
Osteological correlates of the *triceps femoris* insertion and origins of lower leg muscles from the tibiae of Piatnitzkysauridae (left tibiae, lateral view). (a, b) *Piatnitzkysaurus* (PVL 4073). (c, d) *Condorraptor* (MPEF‐PV 1672). Anatomical/muscular abbreviations: cc, cnemial crest; EDLs, *M*. *extensor digitorum longus* scar; fc, fibular crest; it1–3 + amb + fmt, insertion of the tendon from the *iliotibiales* + *ambiens* + *femorotibiales* muscles; lc, lateral condyle; mc, medial condyle; pas, proximal articular surface; si, sulcus intercnemialis; TAd, *M*. *tibialis anterior* depression. Scale bar = 50 mm.

The tibiae of both *Piatnitzkysaurus* specimens, MACN‐Pv‐CH 895 and PVL 4073 (Figure 4a,b), have an expanded and rough area on the tibial cnemial crest with an anterior protuberance, in lateral view, that is distal to the cnemial crest. On this basis, we infer the same condition that is observed in extant archosaurs, with the cnemial crest as the osteological correlate for the insertion of IT1–3 (and the remainder of the *triceps femoris*: AMB and FMTE, FMTI) (level I) (Figure 4a,b). In the *Condorraptor* holotype MPEF‐PV 1672, the cnemial crest is rounded and presents a small ridge (Figure 4c,d) when compared with *Piatnitzkysaurus*, and similar to other archosaurs, this was probably the same attachment area for the main tendon(s) of IT1–3 and other *triceps femoris* muscles (level I) (Figure 4c,d). No tibia associated with *Marshosaurus* has been formally described so far, to our knowledge.

*M. ambiens* (*AMB*): The AMB in extant Reptilia typically takes its origin from the pubic tubercle or *tuberculum preacetabulare* (Hutchinson, 2001b, 2002; Picasso, 2010; Romer, 1923a, 1923b, 1942), also termed the pectineal process (Hudson et al., 1959; Suzuki et al., 2014), preacetabular tubercle (Hutchinson, 2002), or ambiens process (Langer, 2003). In general, this muscle is wider at its origin, becoming more tapered distally. As noted by Hutchinson (2001b), the pubic tubercle is small or even absent in Crocodylia which have a derived feature, relative to other Reptilia, related to having mobile pubes and two heads of AMB (Gregory & Camp, 1918; Hutchinson, 2001b; Romer, 1923a, 1923b; Suzuki et al., 2011). In most Aves, as is ancestral for other non‐archosaurian Reptilia, the AMB has a single head (Hutchinson, 2001b; Picasso, 2010).

**FIGURE 5:**
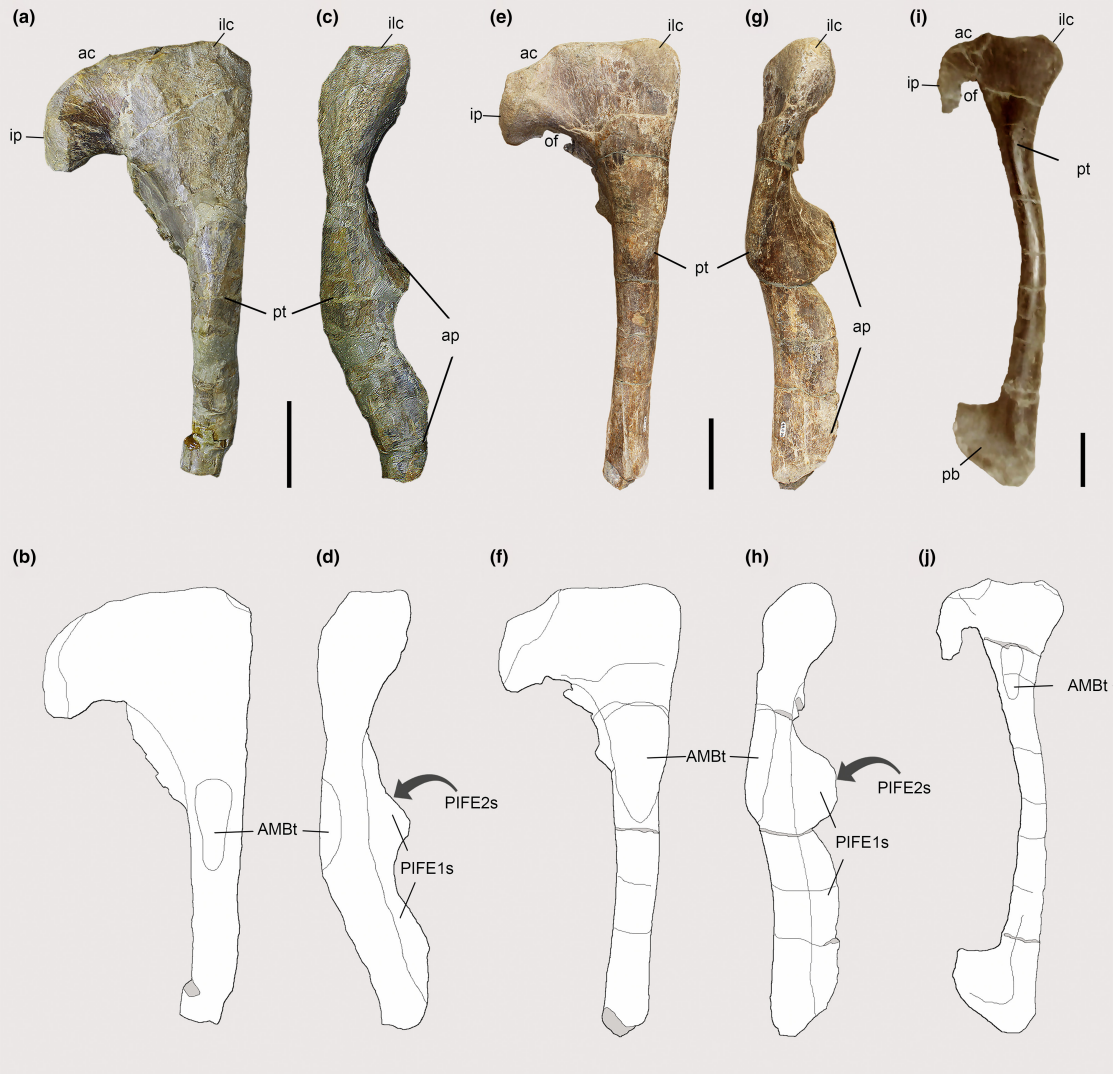
Osteological correlates observed on the pubes of Piatnitzkysauridae (right pubes, lateral and anterior views). (a–d) *Piatnitzkysaurus* (MACN‐Pv‐CH 895). (e–h) *Condorraptor* (MPEF‐PV 1696). (i, j) *Marshosaurus* (UMNH VP 6387). Anatomical/muscular abbreviations: ac, acetabulum; AMBt, *M*. *ambiens* tubercle; ap, apron; ilc, iliac peduncle; ip, ischial peduncle; of, obturator foramen; pb, pubic boot; PIFE1s, *M*. *puboischiofemoralis externus 1* scar; PIFE2s, *M*. *puboischiofemoralis externus 2* scar; pt, pubic tubercle. (a, b, e, f, i, j) in lateral view; (c, d, g, h) in anterior view. Scale bar = 50 mm.

The pubes of both *Piatnitzkysaurus* individuals (left and right in MACN‐Pv‐CH 895 and left in PVL 4073) have a pubic tubercle that is well‐developed (Figure 5a–d), as in Aves and other theropods (Carrano & Hutchinson, 2002; Gregory & Camp, 1918; Grillo & Azevedo, 2011; Hudson et al., 1959; Hutchinson, 2001b; Romer, 1923b). However, this tubercle slightly differs from other piatnitzkysaurid species in position—being more laterally and distally positioned instead of anterior as in *Condorraptor*, and more distally positioned than the condition in *Marshosaurus* (Madsen, 1976) (Figure 5). Nonetheless, the pubic tubercle is an osteological correlate of the presence and origin of the single head of the AMB in *Piatnitzkysaurus* (level I), as previously noted by Bonaparte (1986). The pubic tubercle in *Condorraptor* is remarkably large (Figure 5e–h); this strongly pronounced tubercle generally is not seen in other tetanuran theropods (Rauhut, 2005). It thus is plausible, based on the osteological correlate of the right pubis MPEF‐PV 1696 and a small fragment of the left pubis MPEF‐PV 1688, that the AMB had a robust attachment to the pelvic girdle of *Condorraptor* (level I). The best‐preserved pubis of *Marshosaurus* (right pubis UMNH VP 6387) also osteologically concurs with the single head of the AMB; as previously noted, the anterolateral part of the proximal portion of the pubis presents a visibly rough area (Madsen, 1976) topographically equivalent to the AMB origin (level I) (Figure 5i,j).

The AMB insertion converges onto the tibial cnemial crest with the rest of the triceps femoris muscle group (Hutchinson, 2001b, 2002; Romer, 1923a, 1923b; Suzuki et al., 2014). Furthermore, as noted by Romer (1923a, 1923b), McKitrick (1991), and Hutchinson (2002), the AMB muscle has a secondary tendon which perforates the extensor tendon and merges with the origin of *M*. *gastrocnemius externuslateralis* near the proximal fibula. Although this shared tendon might have been present in early tetanurans such as piatnitzkysaurids, as is ancestral for Archosauria, there is no evidence of it (Level I′).

Thus, as previously described, the insertion of the AMB in *Piatnitzkysaurus* and *Condorraptor* occurred via a shared tendon attached to the cnemial crest (level I) (Figure 4). As there is no formally described tibia for *Marshosaurus*, the insertion of this muscle is not reconstructed here.

*Mm. femorotibiales* (*FMTE and FMTI*): The *Mm*. *femorotibiales* of Crocodylia has two heads (i.e. *M*. *femorotibialis externus*—FMTE and *M*. *femorotibialis internus*—FMTI), whereas in Aves, there are three heads (i.e. *M*. *femorotibialis medialis*—FMTM, *M*. *femorotibialis intermedius*—FMTIM, and *M*. *femorotibialis lateralis*—FMTL) (Clifton et al., 2018; Hudson et al., 1959; Hutchinson, 2001a, 2002; Mckitrick, 1991; Otero et al., 2010; Picasso, 2010; Romer, 1923a; Suzuki et al., 2011; Zinoviev, 2011); here we use the names from Crocodylia as per other studies of non‐avian theropods (e.g., Bishop et al., 2021; Carrano & Hutchinson, 2002; Grillo & Azevedo, 2011). The origins of FMTE and FMTI are located between the trochanteric (proximal) and the condylar (distal) regions across a great portion of the femoral shaft by a fleshy attachment (Carrano & Hutchinson, 2002; Grillo & Azevedo, 2011; Hutchinson, 2001a, 2002; Mckitrick, 1991; Picasso, 2010; Romer, 1923a, 1923b; also see Cuff, Wiseman, et al., 2023). On the femoral shaft, the FMTE and FMTI origins are delimited by three ridges, namely: *linea intermuscularis cranialis* (*lia*), *linea intermuscularis caudalis* (*lip*) and *linea aspera* (=adductor ridge, *la*) (Baumel & Witmer, 1993; Hutchinson, 2001a). However, these structures are variable throughout ontogeny in both extant and extinct archosaurs (Griffin, 2018). The FMTE origin has boundaries delimited by the *lia* and *lip* (on the lateral femoral shaft), whereas the FMTI origin is delimited by the *lia* and *la* (on the anteromedial femoral shaft) (Griffin, 2018; Hutchinson, 2001a, 2002), also there seems to have been the participation of the craniomedial distal crest (cdc) in those subdivisions in some extinct archosaurs (Hutchinson, 2001a).

**FIGURE 6:**
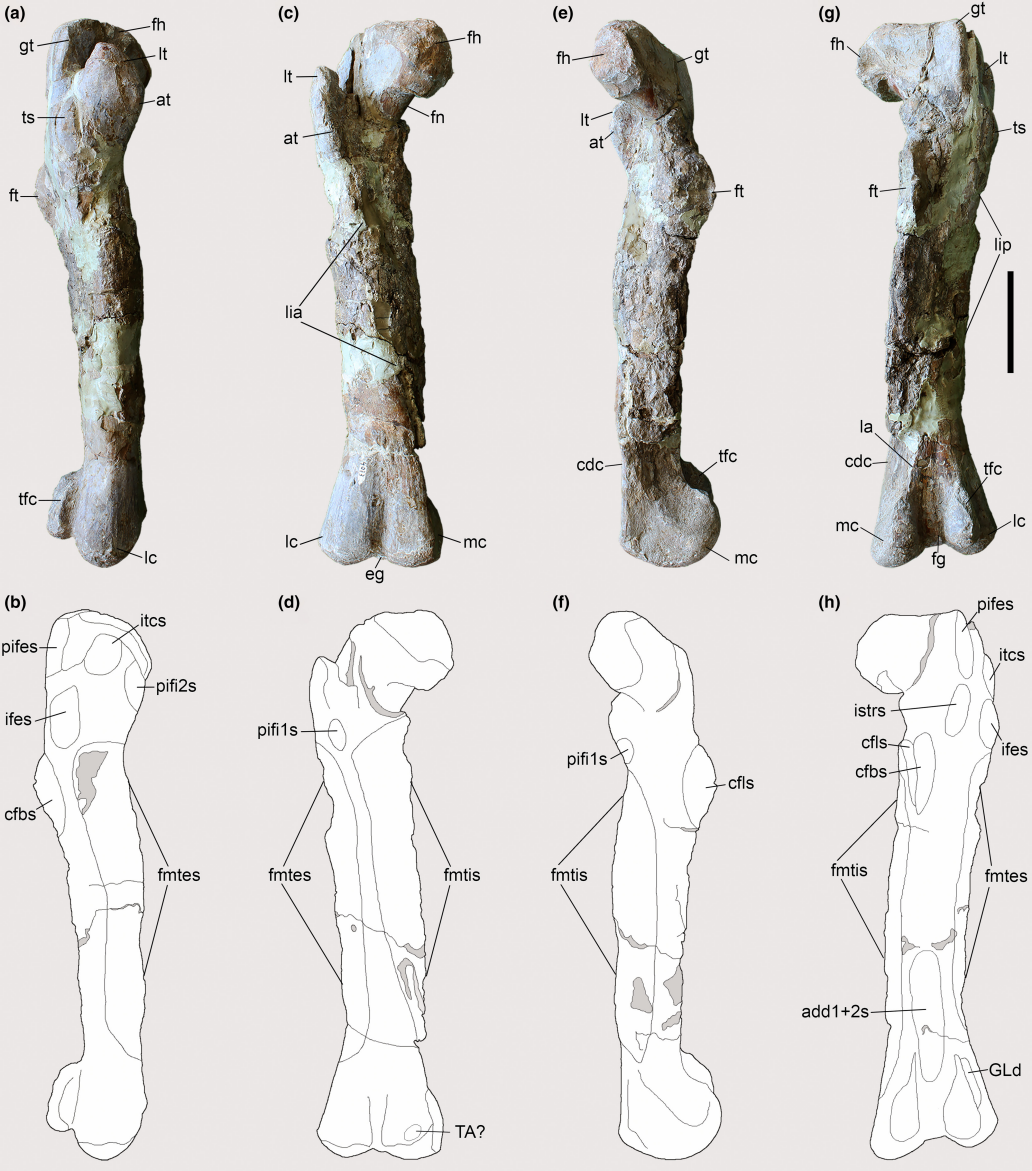
Osteological correlates observed on the femur of *Piatnitzkysaurus* (right femur, PVL 4073). (a, b) lateral view; (c, d) anterior view; (e, f) medial view; (g, h) posterior view. Anatomical/muscular abbreviations: add1 + 2 s, *Mm*. *adductores femores* insertion scar; at, acessory trochanter; cdc, craniomedial distal crest; cfbs, *M*. *caudofemoralis brevis* insertion scar; cfls, *M*. *caudofemoralis longus* insertion scar; eg, extensor groove; fh, femoral head; fg, flexor groove; fmtes, *M*. *femorotibialis externus* scar; fmtis, *M*. *femorotibialis internus* scar; fn, femoral neck; ft, fourth trochanter; GLd, *M. gastrocnemius pars lateralis* depression; gt, greater trochanter; ifes, *M*. *iliofemoralis externus* insertion scar; istrs, *M*. *ischiotrochantericus* insertion scar; itcs, *M*. *iliotrochantericus caudalis* insertion scar; *la*, *linea aspera*; lc, lateral condyle; *lia*, *linea intermuscularis cranialis*; *lip*, *linea intermuscularis caudalis*; lt, lesser trochanter; mc, medial condyle; pifes, *Mm*. *puboischiofemorales externi* insertion scar; pifi1s, *M*. *puboischiofemoralis internus 1* insertion scar; pifi2s, *M*. *puboischiofemoralis internus 2* insertion scar; TA?, *M*. *tibialis anterior*?, origin?; tfc, tibiofibular crest; ts, trochanteric shelf. Scale bar = 100 mm.

**FIGURE 7:**
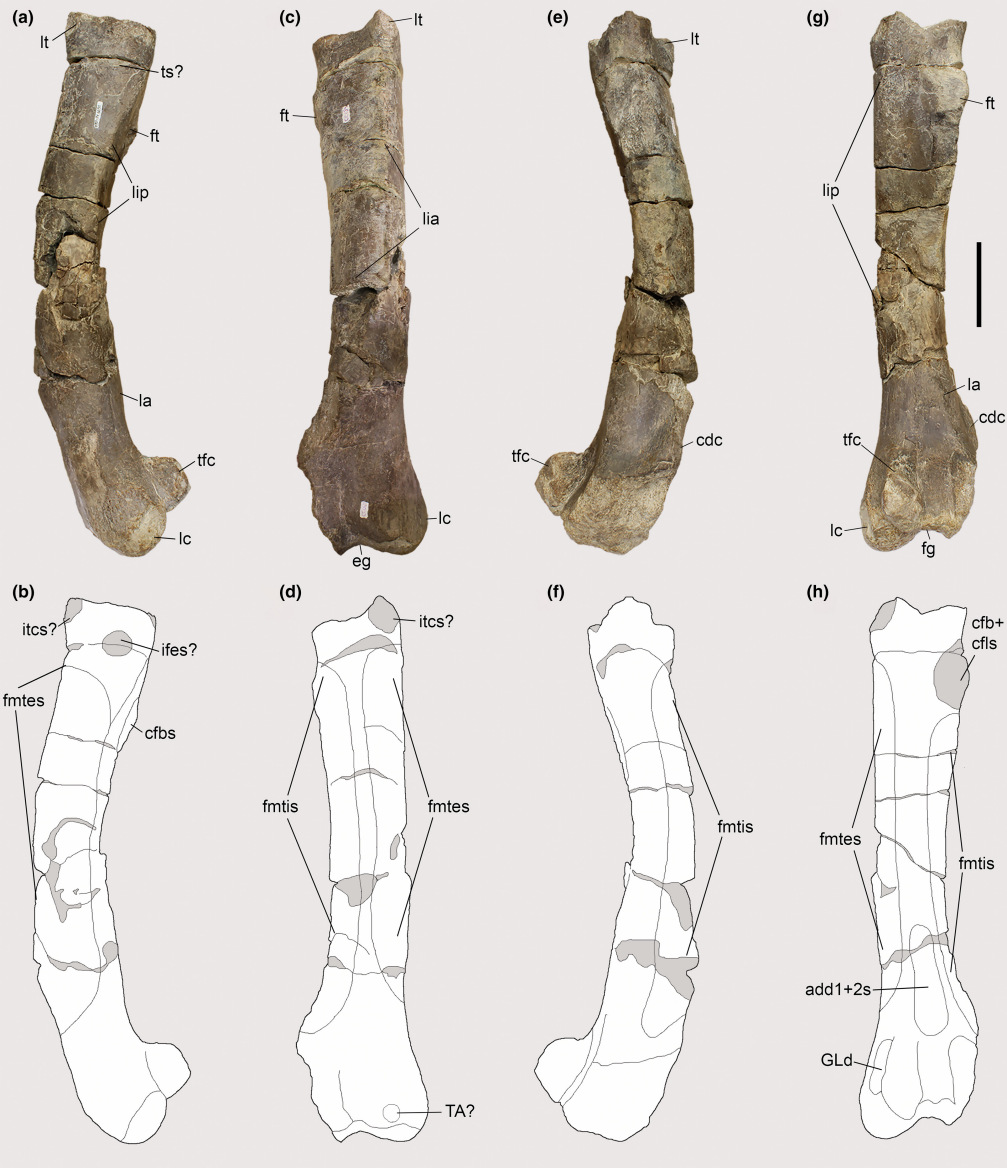
Osteological correlates observed on the femur of *Condorraptor* (left femur, MPEF‐PV 1690). (a, b) lateral view; (c, d) anterior view; (e, f) medial view; (g, h) posterior view. Anatomical/muscular abbreviations: add1 + 2 s, *Mm*. *adductores femores* insertion scar; cdc, craniomedial distal crest; cfb + cfls, *Mm*. *caudofemorales* insertion scar; cfbs, *M*. *caudofemoralis brevis* insertion scar; eg, extensor groove; fg, flexor groove; fmtes, *M*. *femorotibialis externus* scar; fmtis, *M*. *femorotibialis internus* scar; ft, fourth trochanter; GLd, *M. gastrocnemius pars lateralis* depression; ifes?, *M*. *iliofemoralis externus* insertion scar?; itcs?, *M*. *iliotrochantericus caudalis* insertion scar?; *la*, *linea aspera*; lc, lateral condyle; *lia*, *linea intermuscularis cranialis*; *lip*, *linea intermuscularis caudalis*; lt, lesser trochanter; TA?, *M*. *tibialis anterior*?, origin?; tfc, tibiofibular crest; ts?, trochanteric shelf? Scale bar = 100 mm.

The three femora of the two *Piatnitzkysaurus* skeletons lack well‐preserved shaft surfaces. Regardless, the left femur of PVL 4073 preserves the most distal parts of both *la* and *lip* on the posterior shaft of the femur, and *lia* on the distal femur, arising medially and becoming anteriorly positioned along the proximal shaft of the femur (Figure 6c,d). Furthermore, the right femur of PVL 4073 preserves the distal base of the *la* (Figure 6g,h). Although not entirely preserved, the presence of the *la*, *lia* and *lip* allows inference of the FMTE and FMTI origins without precise boundaries (Figure 6). The FMTE and FMTI in *Piatnitzkysaurus*, as well as in other theropods (e.g., *Staurikosaurus*—Grillo & Azevedo, 2011; *Coelophysis*—Bishop et al., 2021; allosauroids—Bates, Benson, & Falkingham, 2012; *Tyrannosaurus*—Carrano & Hutchinson, 2002; *Nothronychus*—Smith, 2021; and *Skorpiovenator*—Cerroni et al., 2022) seem to have had the same origins from the lateral and the anteromedial surfaces of the femoral shaft, respectively (level I). In *Condorraptor*, both femora are quite fragmentary, lacking the proximal portions. The right femur MPEF‐PV 1690 is better preserved, with a great portion of the femoral shaft (Figure 7); however, the three longitudinal ridges/lineae (*lia*, *lip* and *la*) are not completely preserved. It remains possible to reconstruct the FMTE and FMTI origins in positions similar to our *Piatnitzkysaurus* reconstruction (level I), but their proximal extent remains indeterminate. Rauhut (2005) noted the presence of the cdc in both *Condorraptor* femora (Figure 7e–h); this being a structure related to the distal divisions between the FMTE and FMTI origins (Hutchinson, 2001a). *Marshosaurus* has no preserved femur, preventing any inferences about these muscles.

The FMTE and FMTI heads converge into a main tendon and fascia inserting onto the tibial cnemial crest deep to IT1–3 and AMB (level I) (Figure 4), as noted above.

*M*. *iliofibularis* (*ILFB*): In extant Reptilia, the ILFB originates from the lateral surface of the ilium in the postacetabular blade, positioned posterior to the IFE (IF in Crocodylia), anterior to the FTE (FCLA and FCLP in Aves), and ventral to the IT. ILFB is a large, fusiform and superficial muscle of the thigh (Clifton et al., 2018; Dick & Clemente, 2016; Gregory & Camp, 1918; Hutchinson, 2001a, 2001b, 2002; Mckitrick, 1991; Picasso, 2010; Romer, 1923a, 1923b, 1942; Suzuki et al., 2011), more expanded in the ilium of dinosaurs (Hutchinson, 2002).

As previously noted by Bonaparte (1986), the lateral surface of the iliac blade in *Piatnitzkysaurus* has a large and deep depression. This lateral depression is subdivided by a swollen vertical ridge, positioned just above the acetabulum (Carrano et al., 2012; Lacerda et al., 2023). This ridge has been suggested as the anterior limit of the ILFB (Carrano & Hutchinson, 2002; Hutchinson, 2001b). Anterior to the vertical ridge and anterodorsal to the acetabulum, the lateral depression is large and deep; whereas the posterior depression is shallow and positioned just above the ischial peduncle (Figure 2a,b). Topographically, this posterior concavity is equivalent to the ILFB origin, as in other extinct theropods and extant archosaurs (Carrano & Hutchinson, 2002; Grillo & Azevedo, 2011; Hutchinson, 2001a; Otero et al., 2010; Picasso, 2010). The ventral limit of the ILFB origin is indicated by the brevis shelf, and its anterior limits seem to be related to the vertical iliac ridge (Hutchinson, 2001a), whereas the posterodorsal limits appear to have been demarcated by a semi‐circular scar just below the IT3 origin (level I). In *Condorraptor*, although the supraacetabular crest is fragmentary, the left ilium MPEF‐PV 1687 bears a small and shallow concavity dorsal to the ischiadic peduncle and posterior to the supraacetabular vertical ridge, on the postacetabular blade (Figure 2c,d), which may be the osteological correlate for the anterior limits of the ILFB origin. As noted by Carrano and Hutchinson (2002), the scars made by ILFB are difficult to discern; however, a well‐developed iliac ridge lies just above the acetabulum in most megalosauroids (Carrano et al., 2012; Lacerda et al., 2023) and abelisaurids (Cerroni et al., 2022), indicating the anterior edge of the ILFB origin and the posterior edge of the *M*. *iliofemoralis externus*. Ventrally, the concavity related to the ILFB origin is delimited by the brevis shelf. Although the anterior, posterior and ventral limits of the ILFB origin are discernible (level I), the dorsal limit of this muscle origin is unclear, because the supraacetabular rim is not preserved in the only known ilium of *Condorraptor*. The ilia of *Marshosaurus* seem to lack the supraacetabular vertical ridge, or at least taphonomic issues preclude scoring this character in this taxon (Carrano et al., 2012; Lacerda et al., 2023); however, the dorsal, ventral and posterior boundaries of the ILFB origin can be inferred in this species based on the presence of a concavity and its posterior, dorsal and ventral delimitations (level I) (Figure 2e,f).

The insertion of the ILFB in Reptilia is located on the fibular tubercle; a scarred or rounded and prominent structure on the proximal region of the fibular shaft; typically most prominent in archosaurs. Furthermore, a secondary tendon is present in extant taxa (in Aves, constrained by a loop termed *ansa m*. *iliofibularis*—Baumel & Witmer, 1993; also see Hutchinson, 2001a), which inserts onto *M*. *gastrocnemius externus*/*lateralis* near its origin (Dick & Clemente, 2016; Carrano & Hutchinson, 2002; Clifton et al., 2018; Hutchinson, 2001a, 2002; Otero et al., 2010; Picasso, 2010; Romer, 1923a).

**FIGURE 8:**
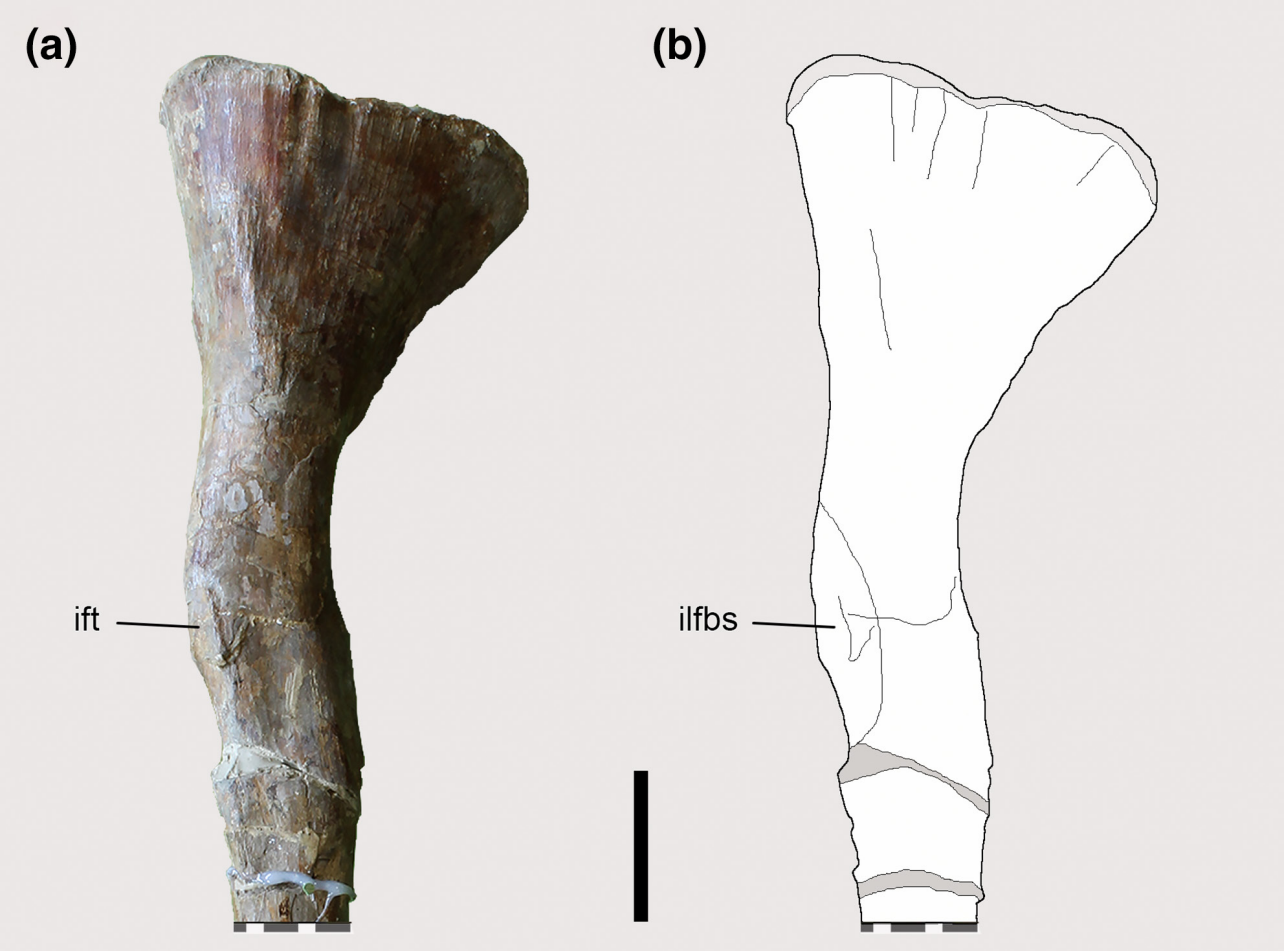
Osteological correlate observed on the left fibula of *Piatnitzkysaurus* (PVL 4073). (a, b) lateral view. Anatomical/muscular abbreviations: ift, iliofibularis (fibular) tubercle; ilfbs, *M*. *iliofibularis* insertion scar. Scale bar = 50 mm.

The right fibula of *Piatnitzkysaurus* PVL 4073 preserves the fibular tubercle (Lacerda et al., 2023), which also presents a small scar (Figure 8), as sometimes seen in other archosaurs. As in other theropods (e.g., *Tyrannosaurus*—Carrano & Hutchinson, 2002), there is no osteological evidence for a secondary tendon in early tetanurans based on *Piatnitzkysaurus*, although this structure is predicted to have been present (level I′). The fibula is not preserved in *Condorraptor* and *Marshosaurus*.

#### 5.1.2 Deep dorsal group

*M. iliofemoralis or M. iliofemoralis externus* (*IFE*) *and M. iliotrochantericus caudalis* (*ITC*): The *M*. *iliofemoralis* in Crocodylia is a single muscle, not divided, with an origin located just above the acetabular aperture and deep to IT2, on the lateral surface of the ilium (Gregory & Camp, 1918; Hutchinson, 2002; Otero et al., 2010; Romer, 1923a, 1923b). In Aves, the “*M*. *iliofemoralis”* is split into two muscles (i.e. *M*. *iliofemoralis externus*—IFE and *M*. *iliotrochantericus caudalis*—ITC; Clifton et al., 2018; Hudson et al., 1959; Hutchinson, 2001a, 2002; Picasso, 2010; Rowe, 1986) which are located above the acetabular aperture (IFE) and on the anteriormost surface of the preacetabular blade (ITC) (Hutchinson, 2002; Rowe, 1986; Suzuki et al., 2014). The subdivision of the *M*. *iliofemoralis* in extant Aves might be evidenced by two insertion areas on the proximal femur (Hutchinson, 2001a). Dinosauromorpha, in general, have a protuberance (lesser/anterior trochanter) on the proximolateral femur (e.g., Müller & Garcia, 2023), homologous with the ITC insertion area in Aves; Dinosauriformes also have a more posterodistal scarred ridge or lump (trochanteric shelf; Novas, 1996) that might correspond to the IFE insertion, suggesting that the *M*. *iliofemoralis* was subdivided in ancestral Dinosauriformes (Carrano & Hutchinson, 2002; Grillo & Azevedo, 2011; Hutchinson, 2001a; Hutchinson & Gatesy, 2000). As per below, *Piatnitzkysaurus* and *Condorraptor* show evidence of this subdivision, too.

Nonetheless, the area of origin of *M*. *iliofemoralis* does not present scars indicating these subdivisions between the IFE and ITC (Carrano & Hutchinson, 2002; Hutchinson, 2001a). We consider the semi‐circular concavity of the *Piatnitzkysaurus* preacetabular ilium (MACN‐Pv‐CH 895; Bonaparte, 1986) anterior to the iliac ridge as the origin of both of these muscular divisions (level I) (Figure 2a,b). The dorsal limits of both muscle origins are quite visible, indicated by striations located just ventral to the origins of the IT1–3 (Figures 2 and 3). The anterior limits of the ITC are undefined in this specimen due to the lack of the anteriormost and anteroventralmost preacetabular blade (Bonaparte, 1986). Following avian myology (e.g., Hutchinson, 2002; Picasso, 2010; Rowe, 1986), the ITC origin presumably would be anterior to the IFE head (level II'). Even though the dorsal rim of the iliac blade is not preserved in *Condorraptor*, a large, deep, almost circular concavity is anterodorsal to the acetabulum (Figure 2c,d); again suggesting the origins of the IFE and ITC (level I). Otherwise, due to the fragmentary nature of the specimen, it is not possible to delimit the boundaries of these muscle origins in this taxon; only to suggest relative general positions. Although the ITC and IFE origins in *Marshosaurus* must have been in a similar pattern, it is not possible to reconstruct this musculature because the anterior part of the ilium is not preserved and the figured ilium (Figure 2e,f) represents a plaster reconstruction of the preacetabular process.

**FIGURE 9:**
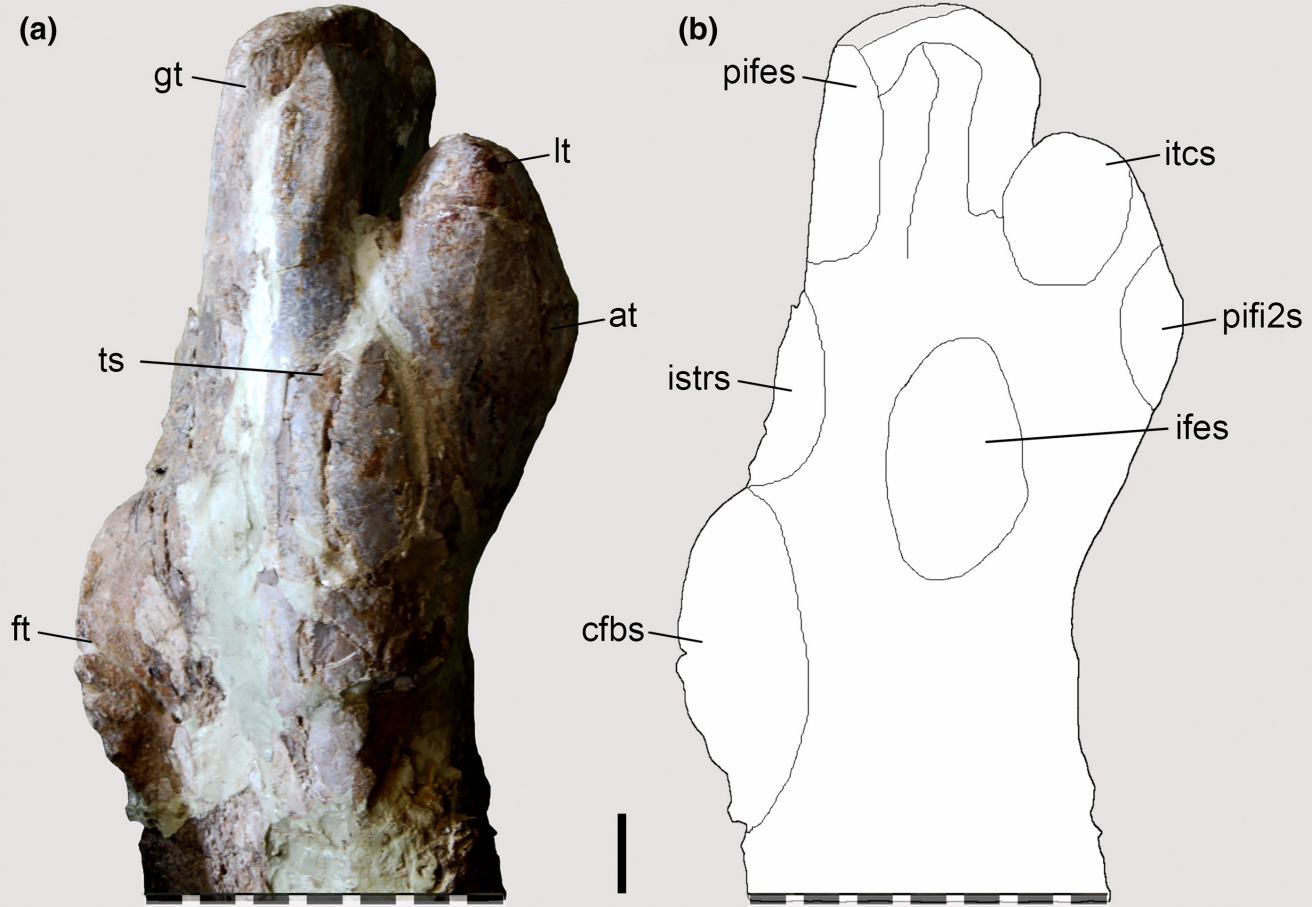
Osteological correlates observed on the femur of *Piatnitzkysaurus* (right femur, PVL 4073). (a, b) lateral view. Anatomical/muscular abbreviations: at, acessory trochanter; cfbs, *M*. *caudofemoralis brevis* insertion scar; ft, fourth trochanter; gt, greater trochanter; ifes, *M*. *iliofemoralis externus* insertion scar; istrs, *M*. *ischiotrochantericus* insertion scar; itcs, *M*. *iliotrochantericus caudalis* insertion scar; lt, lesser trochanter; pifes, *Mm*. *puboischiofemorales externi* insertion scar; pifi2, *M*. *puboischiofemoralis internus 2* insertion scars; ts, trochanteric shelf. Scale bar = 20 mm.

As commented by Bonaparte (1986), the femur of *Piatnitzkysaurus* has a well‐developed lesser trochanter in the shape of a proximodorsally positioned blade (Figures 6a–d and 9); as in other megalosauroids, it rises past the ventral margin of the femoral head (Carrano et al., 2012; Lacerda et al., 2023). A rough area on the trochanteric shelf is not discernible; however, this structure is quite elevated and distinct (Figure 9), being posterodistal to the lesser trochanter and anterodistal to the greater trochanter of the femur. It is thus possible to infer the subdivision of *M*. *iliofemoralis* in this taxon; IFE should have inserted onto the femoral trochanteric shelf (level II) and ITC onto the lesser/anterior trochanter (level II) (Figures 6 and 9). The left femur of *Condorraptor* MPEF‐PV 1690 has the base of the lesser trochanter anterolaterally located, also indicating a quite well‐developed lesser trochanter in *Condorraptor* (and perhaps a fragment of the trochanteric shelf) and IFE and ITC muscle subdivisions (level II) (Figure 7). The femur of *Marshosaurus* is not preserved.

*M. pubo‐ischio‐femoralis internus 1* (*PIFI1*): The PIFI1 in Crocodylia (or *M*. *iliofemoralis internus*—IFI or *M*. “*cuppedicus*” in Aves; Rowe, 1986) is considered to be homologous to the muscles PIFI1 and PIFI2 in Reptilia (Romer, 1923b; Rowe, 1986; Patak & Baldwin, 1999; Hutchinson, 2002; Suzuki et al., 2011) and represents a short, thick muscle. The PIFI1 origin in Crocodylia is located on the medioventral surface of the ilium, as well as on the proximal ischium (Hutchinson, 2001a, 2002; Otero et al., 2010; Romer, 1923a, 1923b). The IFI origin in Aves is on the lateral surface of the ilium, between the anterodorsal region of the pubic peduncle and the posteroventral extremity of the preacetabular blade (Hudson et al., 1959; Hutchinson, 2002; Picasso, 2010; Romer, 1923a; Rowe, 1986; Suzuki et al., 2014). In many extinct theropods, there is evidence of the muscle origin (in a state intermediate between the ancestral reptilian and derived avian condition) from a preacetabular “cuppedicus” fossa (Hutchinson, 2002 [or preacetabular notch—Carrano et al., 2012; Lacerda et al., 2023]) in that same region, suggesting a shift of the muscle origin from the medial to lateral pelvis (Carrano & Hutchinson, 2002; Hutchinson, 2002; Romer, 1923a; Rowe, 1986). This inference is complicated by the fact that homologs of the PIFI2 in Crocodylia also originate from a similar area in Aves, so there is some ambiguity about which PIFI1 or PIFI2 muscle(s) may have shifted into this fossa and when (Carrano & Hutchinson, 2002; Hutchinson, 2001b, 2002).

In *Piatnitzkysaurus*, even though the anterior margin of the preacetabular iliac blade is not entirely preserved on the specimen MACN‐Pv‐CH 895, the “cuppedicus” fossa is evident in the ventromedial surface of the iliac blade (Figure 2a,b), being dorsally delimited by the preacetabular ridge, suggesting the PIFI1 origin (level I). In *Condorraptor* and *Marshosaurus* (Madsen, 1976), despite the fragmentary nature of the ilium of MPEF‐PV 1687 (Figure 2c,d) and UMNH VP 6372 (Figure 2e,f), respectively, the same “cuppedicus” fossa is evident and inferred as the PIFI1 origin (level I).

The PIFI1/IFI insertion in extant archosaurs is located on the anteromedial surface of the femoral shaft. In Crocodylia, the insertion is on a keel that separates the site of insertion of PIFI2 laterally; and the origin of FMTI; medially, anteromedial to the fourth trochanter (Hutchinson, 2001b, 2002; Otero et al., 2010; Romer, 1923a). In Aves, IFI inserts onto a rounded mark on the proximomedial portion of the femur (Hudson et al., 1959; Hutchinson, 2001b, 2002; Suzuki et al., 2014).

The femoral surface in *Piatnitzkysaurus* is not well‐preserved, however, a rounded and small tubercle is positioned distal to the anterior trochanter in both femora PVL 4073, which corresponds to the PIFI1 insertion (level II) (Figure 6d,f). This bump is not discernible on the *Condorraptor* femora MPEF‐PV 1690–1691 (level I′).

*M. pubo‐ischio‐femoralis internus 2* (*PIFI2*) *or M. iliotrochantericus cranialis* (*ITCR*) *and M. iliotrochantericus medius* (*ITM*): The PIFI2 muscle in Crocodylia is considered to be the homologous to the PIFI3 in non‐archosaurian Reptilia instead of the homonymous muscle; however, it is uncertain whether, in the avian lineage, PIFI2 was completely lost (in this hypothesis IF split into four muscles: IFE, ITC, ITCR, and ITM) or whether PIFI2 split into ITCR and ITM in Aves (Carrano & Hutchinson, 2002; Hutchinson, 2002; Romer, 1923a; Rowe, 1986). Even with these uncertainties, the second hypothesis (PIFI2 = ITCR + ITM) is considered better supported, as it requires fewer transformations (Grillo & Azevedo, 2011; Rowe, 1986). Although with variations, most recent theropod reconstructions (e.g., Bishop et al., 2021; Grillo & Azevedo, 2011; Rhodes et al., 2021; Smith, 2023) have adopted the second hypothesis, which is also followed here. In Crocodylia, the PIFI2 is a triangular and broad “fan‐shaped” muscle that originates from the centra of the last 6–7 dorsal vertebrae and the ventral surfaces of their transverse processes (Otero et al., 2010; Romer, 1923a; Suzuki et al., 2011). In Aves, the homologous ITCR and ITM are small muscles that originate from the anteroventralmost part of the lateral portion of the preacetabular iliac blade (Rowe, 1986; Patak & Baldwin, 1998; Picasso, 2010). As above, this evident evolutionary shift of muscle origins is related to the expansion of the preacetabular blade and the origination of the preacetabular notch (Hutchinson, 2001b, 2002; Romer, 1923a).

**FIGURE 10:**
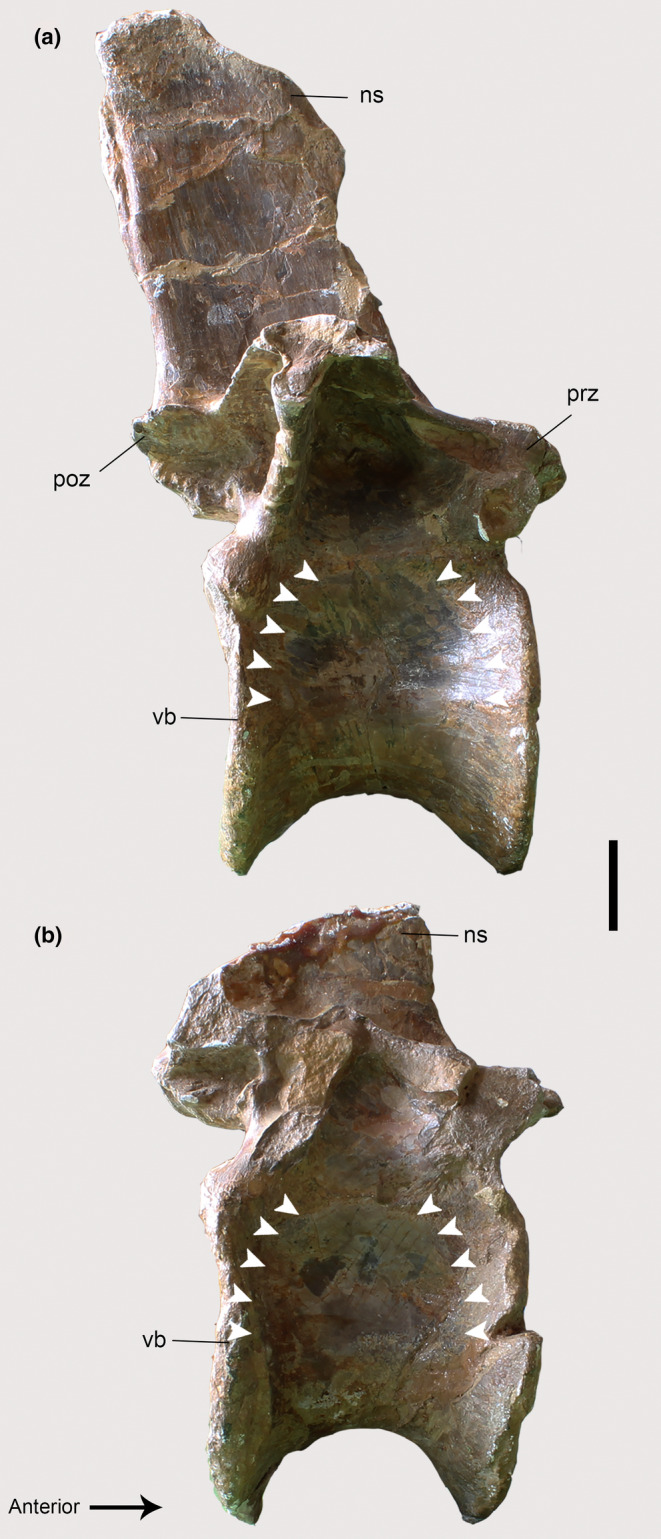
Osteological correlates observed on the vertebrae of *Piatnitzkysaurus* (PVL 4073) in lateral view. (a) 19th dorsal vertebra. (b) 20th dorsal vertebra. Anatomical/muscular abbreviations: ns, neural spine; poz, postzygapophysis; prz, prezygapophysis; vb, vertebral body. Arrows indicate the fossa. Scale bar = 20 mm.

The last dorsal vertebrae in *Piatnitzkysaurus* possess well‐developed vertebral centra (Bonaparte, 1986) lacking pleurocoels. A large and shallow concavity located bellow the parapophyseal region is well‐demarcated on some vertebrae (e.g., 19th and 20th; Figure 10) and could be part of the PIFI2 origin (level II) as in Crocodylia, which also potentially originated near the PIFI1 on the ilium (I′). Only two posteriormost presacral vertebra are preserved in *Condorraptor* (MPEF‐PV 1680 and 1700), with massive vertebral centra (Rauhut, 2005) similar to those of *Piatnitzkysaurus* and also possessing a wide and well‐demarcated shallow concavity that could have been part of the PIFI2 origin (level II). No vertebrae associated with *Marshosaurus* were studied, so no inference was made here.

The PIFI2 insertion in Crocodylia occurs via a tendon on the proximolateral femur near (anterolateral to) the fourth trochanter, on an anteromedial keel at two distinct points separated by the proximal FMTE origin (Hutchinson, 2001a; Otero et al., 2010; Romer, 1923a; Suzuki et al., 2011). In Aves, the homologous muscles insert onto the distal end of the trochanteric crest, marked by small scars (Patak & Baldwin, 1998; Hutchinson, 2001a, 2002). Avetheropoda (Allosauroidea + Coelurosauria; Paul, 1988) evolved a large accessory trochanter, as a “blade‐like” structure that, although small, is also present in some ceratosaurs as well as early Tetanurae (Brissón Egli et al., 2016; Carrano et al., 2012; Cerroni et al., 2022; Hutchinson, 2001a; Lacerda et al., 2023). The accessory trochanter is topologically equivalent to the PIFI2 insertion (Hutchinson, 2001a, 2002).

The right femur of *Piatnitzkysaurus* PVL 4073 preserves a well‐developed blade‐shaped lesser trochanter (Bonaparte, 1986) with a clear anterolateral and distal projection (the accessory trochanter) which is inferred as the insertion of the PIFI2 muscle (level I) (Figures 6a–f and 9). In *Condorraptor*, although the best‐preserved femur MPEF‐PV 1690 has the base of a prominent lesser trochanter (Rauhut, 2005), the most proximal part of it is not preserved and the accessory trochanter is not discernible, so the PIFI2 insertion cannot directly be reconstructed.

#### 5.1.3 Flexor cruris

*M. flexor tibialis internus 1* (*FTI1*): In Aves, the FTI1 muscle is absent (Hutchinson, 2002), whereas in Crocodylia it is a thin and long muscle originating from the distal portion of the ischium, on the posterodorsal surface (Gregory & Camp, 1918; Otero et al., 2010; Romer, 1923a, 1923b; Suzuki et al., 2011) as a relatively long and thin muscle. Other non‐archosaurs lack an obvious FTI1, so homologies are unclear (e.g., PIT in Romer, 1942 or FTI (D) in Dick & Clemente, 2016); originating from the posterior ischium (Dick & Clemente, 2016). Many theropod dinosaurs have a distal ischial tubercle on the posterolateral ischial shaft (e.g., Carrano & Hutchinson, 2002; Hutchinson, 2001b, 2002), which is topographically equivalent to the approximate FT1 origin in Crocodylia.

**FIGURE 11:**
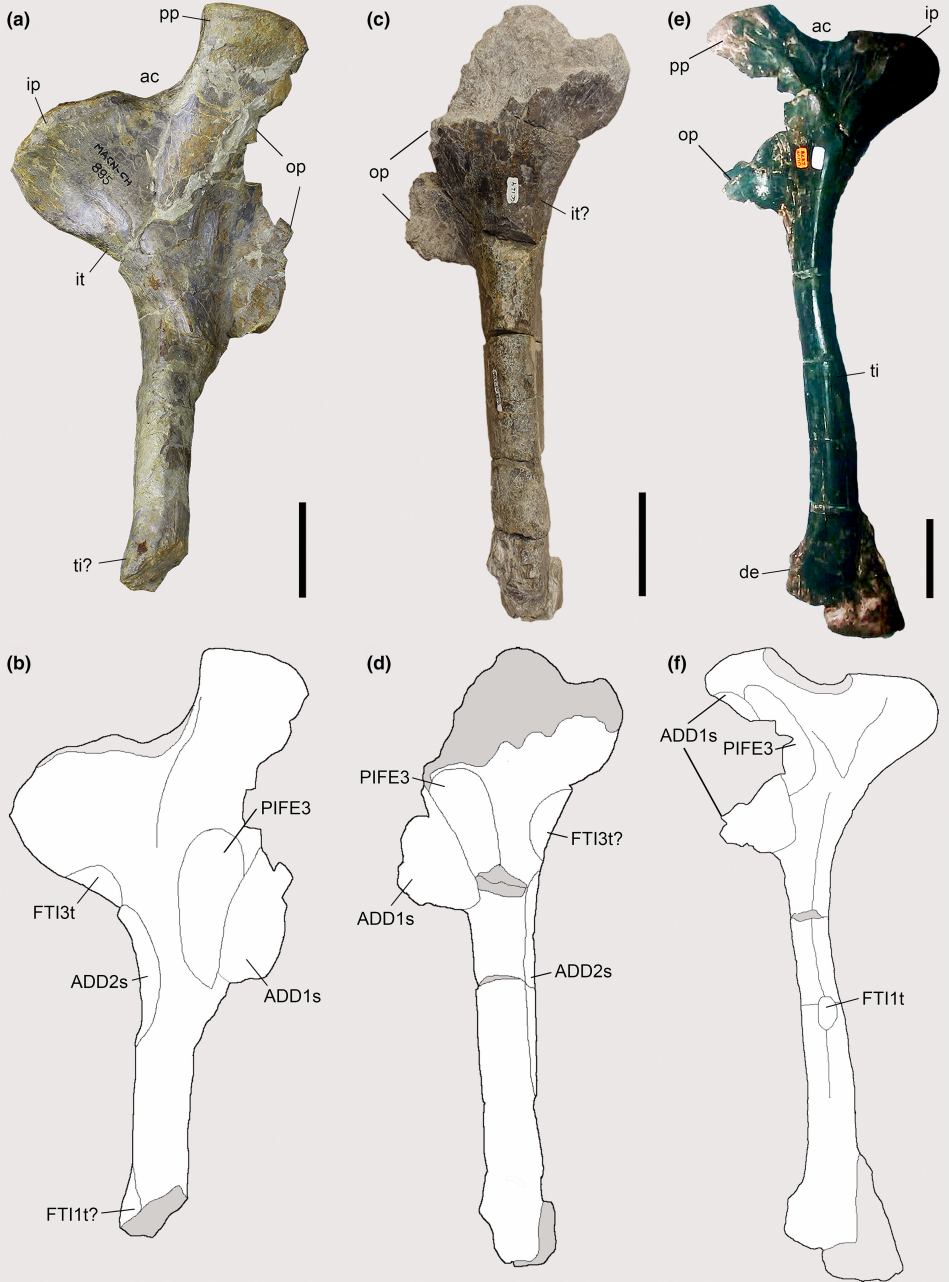
Osteological correlates observed on the ischia of Piatnitzkysauridae. (a, b) *Piatnitzkysaurus* (right ischium, MACN‐Pv‐CH 895). (c, d) *Condorraptor* (left ischium, MPEF‐PV 1696). (d–f) *Marshosaurus* (left ischium, UMNH VP 6387) in lateral view. Anatomical/muscular abbreviations: ac, acetabulum; ADD1s, *M*. *adductor femoris 1* scar; ADD2s, *M*. *adductor femoris 2* scars; de, distal expansion; FTI1t, *M*. *flexor tibialis internus 1* tubercle; FTI3t, *M*. *flexor tibialis internus 3* tubercle; ip, iliac peduncle; it, ischial tuberosity; op, obturator process; pp, pubic peduncle; PIFE3, *M*. *puboischiofemoralis externus 3*; ti, ischiadic tubercle. Scale bar = 50 mm.

Bonaparte (1986) speculated that the ischial tubercle might be the origin of the “*M*. *ischiofemoralis*” (or homologous *M*. *ischiotrochantericus*, ISTR). However, we interpret the ischial tubercle on the distal ischial shaft of the PVL 4073 left ischium as the origin for the FTI1 in *Piatnitzkysaurus*, as a level II inference (Figure 11a,b) (see below for rationale for the ISTR origin). The distalmost portion of the ischial shaft in the ischium of *Condorraptor* MPEF‐PV 1689 is not well‐preserved, with no sign of the ischial tubercle; thus, we made no inference of the FTI1 origin in this taxon. In the left ischium of *Marshosaurus* UMNH VP 6380, although not as discernible as in *Piatnitzkysaurus*, the ischial tubercle appears to be positioned on the medial shaft of the ischium (Figure 11d,e), similar in position to *Piatnitzkysaurus* and topographically equivalent to the FTI1 origin (level II).

In Crocodylia, the FTI1 insertion is onto the medial (Otero et al., 2010; Suzuki et al., 2011) or posterior portion of the proximal tibial metaphysis (Carrano & Hutchinson, 2002), whereas its possible homologue inserts onto the lateral surface of the tibia in other non‐archosaurs (Dick & Clemente, 2016).

**FIGURE 12:**
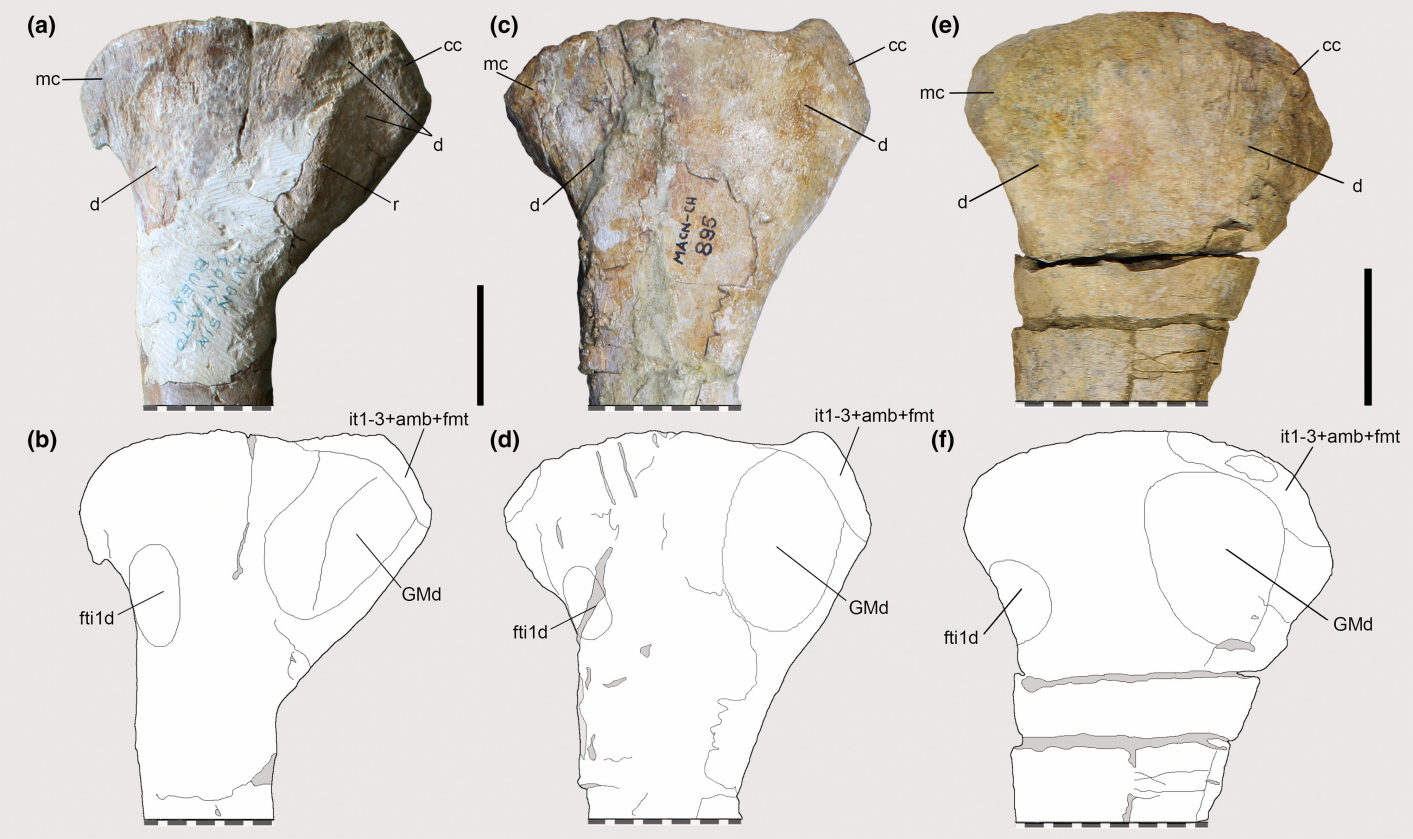
Osteological correlates observed on the tibiae of Piatnitzkysauridae (left tibiae, medial view). (a, b) *Piatnitzkysaurus* (PVL 4073). (c, d) *Piatnitzkysaurus* (MACN‐Pv‐CH 895). (e, f) *Condorraptor* (MPEF‐PV 1672). Anatomical/muscular abbreviations: cc, cnemial crest; d, depression; fti1d, *M*. *flexor tibialis internus 1* depression; GMd, *M*. *gastrocnemius pars medialis* depression; it1–3 + amb + fmt, insertion of the tendons of the *iliotibiales* + *ambiens* + *femorotibiales* muscles; mc, medial condyle; r, ridge. Scale bar = 50 mm.

The posteromedial surface of the proximal region of the tibia in *Piatnitzkysaurus* has a broad depression below the medial condyle (Figure 12a–d), mainly visible in the PVL 4073 specimen (Figure 12a). We interpret this depression as the FTI1 insertion (level II). In *Condorraptor*, a topologically similar depression is also noticeable (Figure 12e), and here considered the FTI1 insertion (level II).

*M. flexor tibialis internus 2* (*FTI2*): In Crocodylia, the FTI2 originates from the lateral ilium, on the postacetabular iliac process just ventral to the origin of FTE (see below); it inserts together with FTI1 and *M*. *puboischiotibialis* onto the posteromedial proximal tibia (Hutchinson, 2002; Otero et al., 2010; Romer, 1923a, 1923b). In Aves, the FTI2 appears to be absent (Hutchinson, 2002). Similar to other theropod dinosaurs such as *Staurikosaurus* (Grillo & Azevedo, 2011), *Coelophysis* (Bishop et al., 2021), and *Tyrannosaurus* (Carrano & Hutchinson, 2002), there are no scars suggesting the presence of FTI2 in *Piatnitzkysaurus* and *Marshosaurus*, so it is ambiguous if this muscle was present or not (Level II'); the postacetabular blade is not well‐preserved in *Condorraptor*, preventing infer anything about this muscle. The latter studies generally considered the FTI2 to more likely be a crocodylian autapomorphy or a trait lost at some early point in Avemetatarsalia–Dinosauromorpha (e.g., Allen et al., 2021; Hutchinson, 2002).

*M. flexor tibialis internus 3* (*FTI3*): In Crocodylia, the FTI3 (=*M*. *flexor cruris medialis*, FCM in Aves; Hutchinson, 2001b) has its origin on the lateral surface of the ischial tuberosity, on the proximolateral portion of the ischium (Otero et al., 2010; Romer, 1923a; Suzuki et al., 2011), which tends to be a scarred area in most non‐avian archosaurs (Hutchinson, 2001b). In Aves, the homologous muscle, FCM, originates from a similar position, although more distally positioned and shifted closer to the ilium via rotation of the ischia (Patak & Baldwin, 1998; Hutchinson, 2002; Picasso, 2010; Suzuki et al., 2014). In other non‐archosaurian Reptilia, the FTI has only two heads, that is, FTI1 and FTI2 (Gregory & Camp, 1918; Hutchinson, 2002; Romer, 1942; Russel & Bauer, 1988). In non‐avian theropods, the origin of the FTI3 is thought to have been from the prominent ischial tuberosity (Bonaparte et al., 1990; Carrano & Hutchinson, 2002; Grillo & Azevedo, 2011; Romer, 1923a, 1923b; Smith, 2021), which gradually shifted its relative position distally to merge with the ilium within stem‐birds (Hutchinson, 2001b).

On the ischium of *Piatnitzkysaurus* MACN‐PV‐CH 895, which is better preserved proximally, a prominent ischial tuberosity that is triangular in shape is present near the proximoposterior edge of the ischium, ventral to the iliac peduncle; we infer this location as the FTI3 origin (level II) (Figure 11a,b). The delimitation of the FTI3 origin in *Condorraptor* is less evident than in *Piatnitzkysaurus*, but is similarly positioned (level II) (Figure 11c,d). In *Marshosaurus* is not possible to determine the FTI3 origin due to a lack of osteological correlates (level II'), so the muscle origin was not reconstructed in any detail, but it should have been in the same location.

The FTI3 in extant archosaurs inserts onto the posterior surface of the proximal portion of the tibia together with the FTE and other FTI head(s), which may form a slightly roughened and rounded structure made by the “tibiocalcaneal tendon” (or ligament) (Otero et al., 2010; Romer, 1923a; Suzuki et al., 2011).

A region topologically related to the FTI3 insertion in *Piatnitzkysaurus* is positioned on the posteromedial surface of the proximal tibia, just below the medial and lateral condyles, and some scarring is proximally located here (level II). Again, in *Condorraptor* there is no scar (level II').

*M. flexor tibialis internus 4* (*FTI4*): The *M*. *flexor tibialis internus* division called FTI4 is only present in the Crocodylia clade (though it may have been lost in *Caiman*; Otero et al., 2010), being the division equivalent to the superficial portion of FTI2 of other non‐archosaurian Reptilia (Romer, 1942). It is a small and thin muscle that originates from the fascia around the posteroventral ilium and posterodorsal ischium (Romer, 1923a, 1923b; Suzuki et al., 2011). Since this muscle leaves no evident scars and is absent in Aves (Carrano & Hutchinson, 2002; Hutchinson, 2002), the presence in *Piatnitzkysaurus* and *Marshosaurus* is equivocal (level II') so we do not infer this muscle here. The condition is even more ambiguous in *Condorraptor*, as the posterior portion of the ilium is not well‐preserved. Following prior studies, we assume that the FTI4 is a crocodylian autapomorphy, absent in theropods.

*M. flexor tibialis externus* (*FTE*): The FTE muscle (=*M*. *flexor cruris lateralis pars pelvica*, FCLP in Aves) in extant archosaurs is a large muscle originating from the posterolateral surface of the ilium, just posterior to the origins of ILFB and IFE, and dorsal to *M*. *caudofemoralis brevis* on the postacetabular blade (Gregory & Camp, 1918; Hutchinson, 2002; Otero et al., 2010; Picasso, 2010; Romer, 1923a, 1923b; Suzuki et al., 2014). In other non‐archosaurian Reptilia, the FTE also originates from the posterior ilium and ilioischiadic ligament (e.g., Dick & Clemente, 2016; Romer, 1942).

On the posterolateral region of the *Piatnitzkysaurus* ilium MACN‐Pv‐CH 895, above the brevis shelf and below the IT3 origin, there are some linear scars topographically equivalent to the position of the FTE origin in extant archosaurs. We thus infer that the FTE origin was located here (level I), as in other dinosaurs (e.g., Bishop et al., 2021; Carrano & Hutchinson, 2002; Grillo & Azevedo, 2011; Langer, 2003; Russell, 1972; Smith, 2021). Yet as noted by Bonaparte (1986), the posterior edge of the postacetabular blade of the *Piatnitzkysaurus* ilium is not entirely preserved, thus, the posterior limits of the FTE origin remain unclear. In *Marshosaurus* left ilium UMNH VP 6372, it is also possible to infer the FTE origin due to some anterior delimitations located posterior to the ILFB (level I) (Figure 3b). Because the postacetabular blade of *Condorraptor* is not preserved, is not possible to directly infer the FTE origin.

As the FTE inserts very close to the FTI3, or shares a common tendon in extant archosaurs (e.g., Otero et al., 2010; Romer, 1923a, 1923b), this applies to *Piatnitzkysaurus* (level I), but more is equivocal in *Condorraptor* (level I′) and not possible to infer in *Marshosaurus*.

#### 5.1.4 Mm. Adductores Femores

*M. adductor femoris 1* (*ADD1*): In extant archosaurs, the ADD1 (=*M*. *puboischiofemoralis pars medialis*, PIFM in Aves; Hutchinson, 2001a, 2001b) has its origin from the anterolateroventral surface of the ischium by a fleshy attachment, located on the equivalent of the obturator process (ischial apron/anteroventralmost ischial shaft) (Hudson et al., 1959; Hutchinson, 2001a, 2001b, 2002; Mckitrick, 1991; Picasso, 2010; Romer, 1923a; Suzuki et al., 2011, 2014). However, no clear osteological correlate for this origin is evident on the ischial surface (Carrano & Hutchinson, 2002). Only one head of the *adductor femoris* is present in non‐archosaur Reptilia, originating from the puboischiadic ligament (Dick & Clemente, 2016; Romer, 1942).

The incomplete obturator process in *Piatnitzkysaurus* extends proximally, almost to the anterior line of the pubic peduncle (Bonaparte, 1986). Some scars are visible on the most anterodorsal portion of the ischial apron (mainly in the PVL 4073 specimen), which could be related to the puboischiadic membrane (Hutchinson, 2002). Based on relative positions, the ADD1 in *Piatnitzkysaurus* probably originated from the anteroventral obturator process of the ischium in the ventral portion of the ischial apron (level I′) (Figure 10a,b). In *Condorraptor* MPEF‐PV 1689, the obturator process is damaged, making it difficult to determine its anterior contact with the pubis. However, even in its broken state it obviously is a developed structure (Rauhut, 2005) (Figure 10c,d), and the same is noted in *Marshosaurus* UMNH VP 6380 (Madsen, 1976) (Figure 10d,e). A few scars are evident in the obturator process of the ischium; thus, this region probably was the site of origin of the ADD1 (level I′).

The ADD1 in extant archosaurs has a small, somewhat tendinous insertion located on the posterior shaft of the distalmost femur (Hutchinson, 2001a; Otero et al., 2010; Picasso, 2010; Romer, 1923a; Suzuki et al., 2014); medial to the ADD2; with both insertions located between the *la* and *lip* (Hutchinson, 2001a).

Although there is no osteological correlate for the ADD1 insertion discernible in *Piatnitzkysaurus*, some delimitations are on the *Condorraptor* left femur MPEF‐PV 1690, in a location topologically equivalent to that in other archosaur fossils (e.g., Bishop et al., 2021; Carrano & Hutchinson, 2002; Dilkes, 2000; Grillo & Azevedo, 2011; Hutchinson, 2001a, 2002; Russell, 1972; Smith, 2021, 2023) (level I′ for *Piatnitzkysaurus* and level I for *Condorraptor*; Figures 6 and 7); however, see comments below.

*M. adductor femoris 2* (*ADD2*): The ADD2 (=*M*. *puboischiofemoralis pars lateralis*, PIFL in Aves; Hutchinson, 2001a, 2001b) originates from a fleshy attachment on the posterior portion of the ischium on the edge distal to the FTI3 and the ischial tuberosity in Crocodylia, although in Aves this origin is more anteroventral (Hudson et al., 1959; Mckitrick, 1991; Patak & Baldwin, 1998; Hutchinson, 2001b; Otero et al., 2010; Picasso, 2010; Suzuki et al., 2011, 2014).

A small depression is evident on the posterodorsal rim of the right ischium of *Piatnitzkysaurus* MACN‐Pv‐CH 895, distally delimited by a bump. This position is topographically equivalent to the inferred ADD2 origin in other theropods (e.g., Bishop et al., 2021; Carrano & Hutchinson, 2002; Grillo & Azevedo, 2011; Smith, 2021). Although no roughened scars are discernible, this depression is interpreted as the origin of ADD2 (level II) (Figure 11a,b). The osteological correlate of the ADD2 origin on the ischium of *Condorraptor* is less evident, but can be delimited in a position similar to that of *Piatnitzkysaurus*, but extending further distally (level II) (Figure 11c,d). In *Marshosaurus*, the ADD2 boundaries were not observed, therefore this muscle's origin was not reconstructed.

The ADD2 insertion in extant archosaurs is located on the posterior shaft of the femur, lateral to the ADD1 (Hutchinson, 2001a; Otero et al., 2010; Picasso, 2010; Romer, 1923a, 1923b; Suzuki et al., 2014); as above.

**FIGURE 13:**
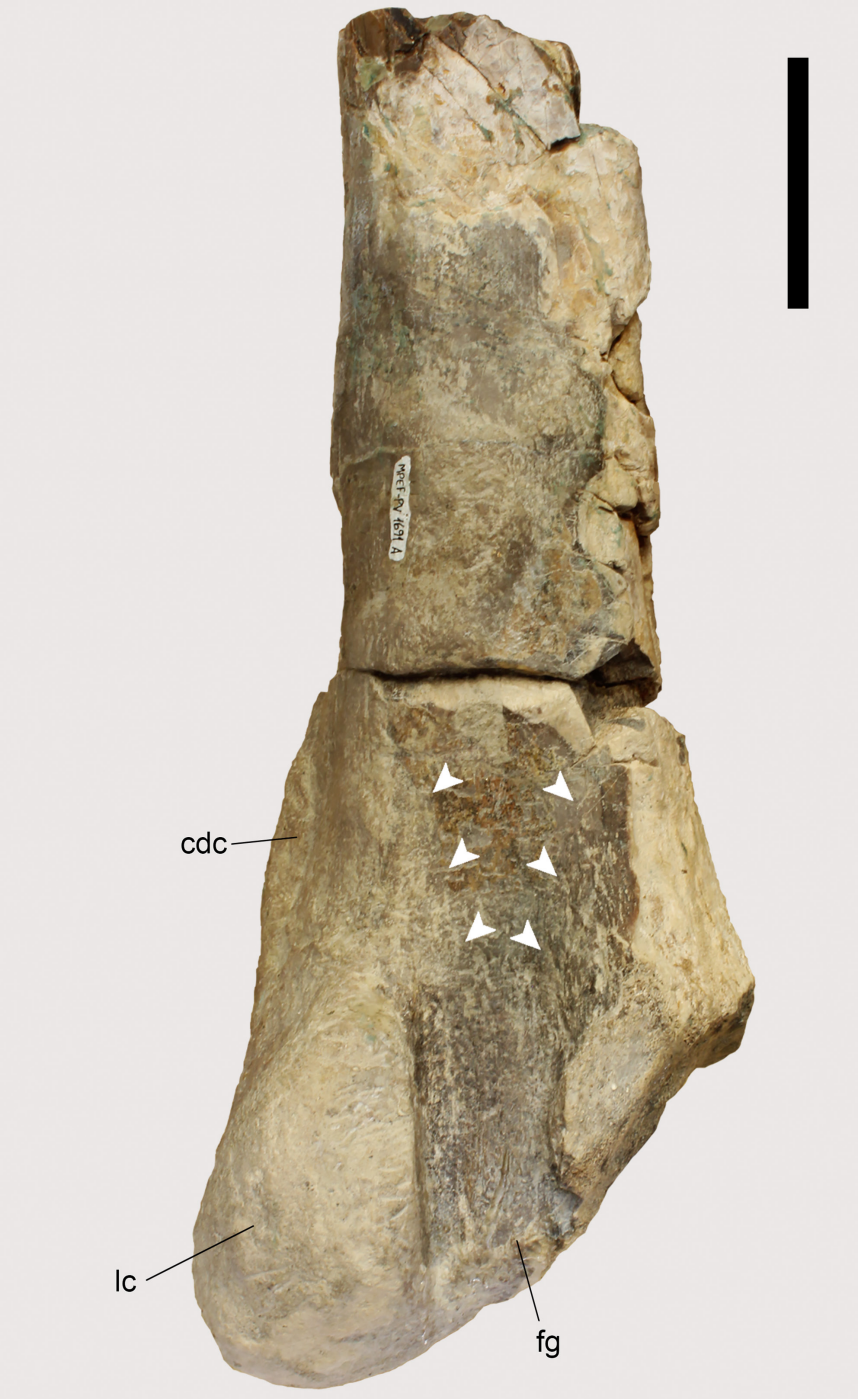
Possible *Mm*. *adductores femores* division on the *Condorraptor* distal right femur (MPEF‐PV 1691, posterior view). Anatomical abbreviations: cdc, craniomedial distal crest, fg, flexor groove; lc, lateral condyle. Arrows indicate these scars that may or may not pertain to ADD 1 + 2 (see text). Scale bar = 50 mm.

Again, we infer an ADD2 insertion in the same relative position as in other extinct archosaurs (e.g., Bishop et al., 2021; Carrano & Hutchinson, 2002; Dilkes, 2000; Grillo & Azevedo, 2011; Hutchinson, 2001a, 2002; Romer, 1923b; Russell, 1972; Smith, 2021). No scars are noted on the posterior shaft of the femoral diaphysis in *Piatnitzkysaurus* (level I′), but discernible marks can be noted in *Condorraptor* (level I) (Figures 6 and 7). As these scars in both studied species are not distinct, we conservatively reconstructed both insertions, i.e., ADD1 + 2, in a single region (Figures 6 and 7). However, based on the right femur (MPEF‐PV 1691) of *Condorraptor*, some scars might indicate where these muscles were inserted separately (Figure 13), although we are cautious to interpret it this way, as these potential ADD1 + 2 boundaries are more distally positioned than observed in other theropods (Carrano & Hutchinson, 2002).

#### 5.1.5 Mm. Puboischiofemorales Externi

*M. puboischiofemoralis externus 1* (*PIFE1*): In Crocodylia, the PIFE1 originates from the anteromedial surface of the pubic apron and epipubic cartilage by a fleshy attachment (Otero et al., 2010; Romer, 1923a, 1923b; Suzuki et al., 2011). The later structure corresponds to an anteromedially expanded surface of the pubic symphysis (Hutchinson, 2001b). In Aves, a pubic symphysis is absent; thus, the origin of PIFE1 homologue (i.e., the small *M*. *obturatorius lateralis*; Hutchinson, 2001b) is from the proximolateral surface of the pubis, close to the acetabulum (Patak & Baldwin, 1998; Hutchinson & Gatesy, 2000; Hutchinson, 2002; Picasso, 2010; Suzuki et al., 2014). However, in some Aves (presumably autapomorphically), the origin of this muscle has two parts, originating both from pubis and ischium surrounding the obturator foramen (e.g., Gangl et al., 2004). Only one head (or weak subdivision) of the PIFE is present in other non‐archosaurian Reptilia (Hutchinson, 2002; Romer, 1942).

Even though the distal regions of the pubes in *Piatnitzkysaurus* MACN‐Pv‐CH 895 are lacking due to the taphonomy of this specimen, an expanded pubic apron is on the anteromedial pubis, confirmed in the PVL 4073 specimen (Figure 5a–d), and similar to the condition in other non‐avian theropods (e.g., Bishop et al., 2021; Carrano & Hutchinson, 2002; Grillo & Azevedo, 2011; Hutchinson, 2001b; Rhodes et al., 2021). As in Crocodylia, in *Piatnitzkysaurus* this likely was the PIFE1 origin on the anterior surface of the pubes (level II) (Figure 5c,d). Similarly, the pubic aprons of *Condorraptor* and *Marshosaurus* are well‐developed but not entirely preserved, and consistent with the same PIFE1 origin (level II) (Figure 5g,h).

The PIFE1–3 in extant archosaurs have a common tendon of insertion that attaches to the proximolateral femur on the greater trochanter (Hutchinson, 2001a, 2002; Otero et al., 2010; Romer, 1923a).

The greater trochanter in *Piatnitzkysaurus* has a straight angle to the femoral long axis (Bonaparte, 1986) and we infer it to represent the PIFE1 insertion (level I) (Figures 6 and 9). This structure is not preserved in *Condorraptor*.

*M. puboischiofemoralis externus 2* (*PIFE2*): In Crocodylia, the PIFE2 (=*M*. *obturaturious medialis*, OM in Aves; Hutchinson, 2001b) is a fan‐shaped muscle, originating from the posterior surface of the pubic apron, on the posterolateral pubis (Otero et al., 2010; Romer, 1923a, 1923b; Suzuki et al., 2011). Contrastingly in Aves, the large homologous muscle, OM, is more posteriorly positioned (via pubic retroversion) and originates medially from the puboischiadic membrane (Patak & Baldwin, 1998; Hutchinson, 2001b, 2002; Suzuki et al., 2014).

Considering the well‐developed pubic apron in the piatnitzkysaurids studied here, a level II inference allows us to infer that these taxa had a PIFE2 origin from the posterior portion of the pubic apron (Figure 5). The insertion with PIFE1–3 is described above (Figures 6 and 9).

*M. puboischiofemoralis externus 3* (*PIFE3*): The PIFE3 in Crocodylia has a large fleshy origin from the anterolateral surface of the ischium, on the obturator process between the origins of ADD1 + 2, and anterodorsally delimited by the ischial ridge (Hutchinson, 2001b, 2002; Otero et al., 2010; Romer, 1923a, 1923b; Suzuki et al., 2011). In Aves, the obturator process of the ischium is lost, as well as the third head of PIFE (Hutchinson, 2001b, 2002).

The retention of the obturator process in *Piatnitzkysaurus*, *Condorraptor*, and *Marshosaurus* (Figure 11), as well as in other non‐avian theropods (e.g., Triassic *Coelophysis*—Bishop et al., 2021; and Cretaceous *Tyrannosaurus*—Carrano & Hutchinson, 2002) is indicative of the PIFE3 origin. However, variations in the size and shape of the theropod puboischiadic plate throughout evolution indicate some variation in the size of the musculature associated with this region (Lacerda et al., 2023). Although the PIFE3 origin's exact limits are undefined, it probably was located anteroventral to the ischial ridge on the posterior portion of the obturator process, similar to the position in Crocodylia and other theropod species, such as *Staurikosaurus* (Grillo & Azevedo, 2011) and *Coelophysis* (Bishop et al., 2021). In the right ischium of *Piatnitzkysaurus* MACN‐Pv‐CH 895, the probable origination site is more evident, being positioned between the ADD1 origin and the ischial ridge (level II) (Figure 11a,b). In the poorly preserved ischia of PVL 4073, as well as in *Condorraptor* and *Marshosaurus*, the PIFE3 boundaries are not possible to reliably estimate, but the PIFE3's general position is (level II) (Figure 11c–f). See above for details on the PIFE 1–3 insertion (Figures 6 and 9).

*M. ischiotrochantericus* (*ISTR*): The ISTR in non‐avian Reptilia including Crocodylia has a single head originating from the medial surface of the ischium (Gregory & Camp, 1918; Hutchinson, 2001b, 2002; Otero et al., 2010; Romer, 1923a; Suzuki et al., 2011). The ADD2 and FTI3 origins on the posterolateral margin of the ischium form the posterolateral boundary of the ISTR origin. In Aves, the homologous muscle (the large, fusiform *M*. *ischiofemoralis*, ISF; Hutchinson, 2001b) has shifted its origin to the lateral side of the ischium and ilioischiadic membrane (Hudson et al., 1959; Hutchinson, 2002; Picasso, 2010; Romer, 1923a, 1923b; Suzuki et al., 2014).

Among the three piatnitzkysaurids studied here, the *Piatnitzkysaurus* right ischium of the MACN‐Pv‐CH 895 specimen is the best preserved proximally; followed by the *Marshosaurus* left ischium UMNH VP 6380, which has both, iliac and pubic peduncles, but lacks the ventral part of the obturator process (Madsen, 1976); and the *Condorraptor* left ischium MPEF‐PV 1689, which although lacking most of the proximal articulation, preserves a partial, well‐developed obturator process (Rauhut, 2005). None of the ischia of the three taxa presents evidence of the apomorphic condition of lateral origin of the ISTR muscle; in both *Piatnitzkysaurus* and *Marshosaurus* (Madsen, 1976), the medial surface of the ischium/obturator process is covered by fine striations. In *Condorraptor*, such striations are not discernible (there might be small ventral marks on the obturator process, if not taphonomic artifacts), but *Condorraptor* probably had the same origin. Therefore, the ISTR origin was on the medial surface of the ischium, including the entire area of the obturator process (level II for *Piatnitzkysaurus* and *Marshosaurus*, and level II' for *Condorraptor*).

In extant archosaurs, the ISTR insertion is by a tendinous attachment to the posteroproximal portion of the lateral femur, distal to the greater trochanter and PIFE1–3 insertions (Clifton et al., 2018; Hutchinson, 2001a, 2002; Otero et al., 2010; Romer, 1923a; Suzuki et al., 2014). In non‐avian theropods (e.g., Bishop et al., 2021; Carrano & Hutchinson, 2002; Grillo & Azevedo, 2011; Smith, 2021, 2023), the insertion presumably occurred between the greater and the fourth trochanters, onto the posterior portion of the trochanteric shelf (Hutchinson, 2001a).

The best‐preserved femur of *Piatnitzkysaurus* (right femur PVL 4073) has a clear posteriorly projected structure proximal to the fourth trochanter and distal to the greater trochanter (Figure 9), similar in position and shape to other tetanurans (e.g., *Tyrannosaurus*—Carrano & Hutchinson, 2002). Although the bony surface is not well‐preserved, this projection is preceded anteroposteriorly by a groove considered here as the insertion of ISTR (level I) (Figures 6 and 9). Due to the fragmentary nature of the proximal femur of *Condorraptor* MPEF‐PV 1690 and the lack of femora for *Marshosaurus*, no inferences were made about the ISTR insertion for these taxa.

#### 5.1.6 Mm. Caudofemorales

*M. caudofemoralis brevis* (*CFB*). The CFB muscle is small in non‐avian Reptilia and originates from the medial and partially the lateral surfaces of the ilium, on its postacetabular blade from a shallow fossa, as well as from the posterior sacral ribs (Gatesy, 1990; Hutchinson, 2002; Otero et al., 2010; Romer, 1923a, 1923b). In Aves, the homologous (i.e., the large *M*. *caudofemoralis pars pelvica*, CFP; Hutchinson, 2001b) also has a single head, but it originates from the posteroventral surface of the lateral ilium (Clifton et al., 2018; Hutchinson, 2002; Mckitrick, 1991; Picasso, 2010). Accordingly, following Hutchinson (2001b, 2002), in non‐avian dinosaurs, the CFB origin mainly was from the brevis fossa of the posteroventral ilium, a structure that was gradually reduced across the lineage to Aves as the CFB origin shifted laterally (Hutchinson, 2001b).

**FIGURE 14:**
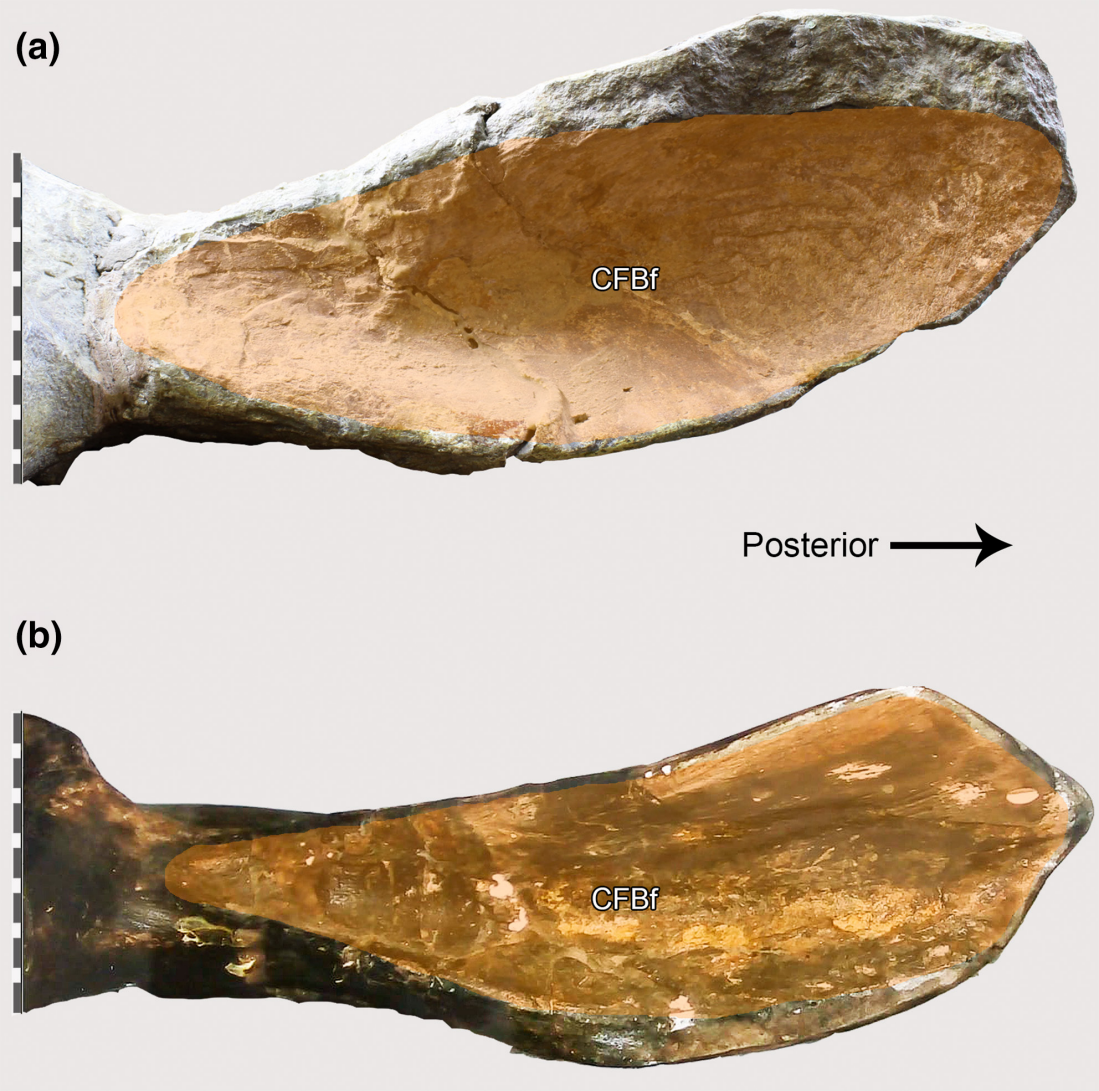
Ventral view of the brevis fossa in Piatnitzkysauridae. (a) *Piatnitzkysaurus* (PVL 4073). (b) *Marshosaurus* (UMNH VP 6372). Anatomical/muscular abbreviations: CFBf, *M*. *caudofemoralis brevis* fossa. Not to scale.

The posterior width of the brevis fossa varies in non‐avian theropods (Carrano et al., 2012). In some tetanurans, the brevis fossa is posteriorly wide in *Marshosaurus* (Figure 14) and some spinosaurids; whereas it is subequal in width in *Piatnitzkysaurus* and some megalosaurids (Lacerda et al., 2023). The *Piatnitzkysaurus* ilium MACN‐Pv‐CH 895 has a large and relatively deep brevis fossa, and presumably the CFB in this taxon originated entirely in the fossa (level II), although the posterior edge of the postacetabular blade in this specimen is incomplete (Figure 14a). The postacetabular process of the ilium in *Condorraptor* MPEF‐PV 1687 does not have the brevis fossa preserved, so although it almost certainly existed (level II'), we do not infer details of it here. Regarding *Marshosaurus*, Madsen (1976) commented that the brevis fossa of the UMNH VP 6373 ilium has a shallow concavity and holds the CFB muscle; based on our studied specimen UMNH VP 6372, this shallow and posteriorly enlarged fossa is the osteological correlate of the CFB muscle origin (level II) (Figure 14b).

The CFB muscle of extant archosaurs inserts by a tendon on the posterolateral surface of the proximal region of the femur, positioned between the *lip* and the fourth trochanter (Hutchinson, 2001b, 2002; Otero et al., 2010; Picasso, 2010; Suzuki et al., 2014).

Among the femora of *Piatnitzkysaurus*, the better‐preserved fourth trochanter is on the right femur of PVL 4073 specimen; however, the surface between the fourth trochanter and the *lip* is not well‐preserved and the insertion of CFB is not discernible based on scars, even though the well‐developed fourth trochanter allows us to infer the insertion of this muscle safely (level I) (Figures 6 and 9), based on that in extant and extinct archosaurs. The *Condorraptor* left femur MPEF‐PV 1690 preserves a well‐developed fourth trochanter. As noted by Rauhut (2005) it is a low but robust ridge, allowing us to infer the position and extent of the CFB insertion (level I) (Figure 7).

*M. caudofemoralis longus* (*CFL*): In non‐avian Reptilia, the CFL (=*M*. *caudofemoralis pars caudalis*, CFC in Aves; Gatesy, 1990; Hutchinson, 2001b) has a large fleshy origin from the caudal vertebrae, including the ventral surface of the transverse processes and haemal arches, beginning around the 12th caudal vertebra (Gatesy, 1990; Hutchinson, 2002; Otero et al., 2010; Wilhite, 2023). In most Aves (where present), the CFC origin is restricted to the last free caudal vertebra and the uropygium (Clifton et al., 2018; Hutchinson, 2001a, 2002; Suzuki et al., 2014).

Although the tail in *Piatnitzkysaurus* and *Condorraptor* is poorly known, and any vertebral elements from *Marshosaurus* lack formal description, it is possible to reconstruct the CFL origin in the South American piatnitzkysaurids. Two caudal vertebrae are preserved in *Piatnitzkysaurus* PVL 4073 specimen, probably the 2nd and 4th (Bonaparte, 1986); both feature robust centra and transverse processes related to the CFL origin (level I). Three caudal vertebrae are known for *Condorraptor*; the proximalmost, MPEF‐PV 1702, has a tall centrum with posterodorsal oval depression and a dorsolaterally, slightly posteriorly directed transverse process; the proximal mid‐caudal vertebra, MPEF‐PV 1682, has a more elongated centrum with a shallow depression and a prominent laterally/slightly dorsally and posteriorly directed transverse process; and the distal mid‐caudal vertebra, MPEF‐PV 1683, has a low and elongated centrum with no sign of a transverse process (Rauhut, 2005). These characteristics of the proximalmost and the proximal mid‐caudal vertebrae allow inferring part of the CFL origin (level I).

The CFL in non‐avian Reptilia inserts onto the proximal femur, in the pit and on the medial surface of the fourth trochanter; a secondary tendon continues downwards to the fibula, contributing to the *M*. *gastrocnemius externus* (=*lateralis* of Aves) origin (Gatesy, 1990; Hutchinson, 2001a, 2002; Otero et al., 2010; Romer, 1923a). Once birds reduced their tail, the CFC muscle reduced as well as the fourth trochanter which reduced to a roughed area (Gatesy, 1990). Dinosauromorphs and theropods have a large crest‐shaped fourth trochanter (e.g., Hutchinson, 2001a); this also being the condition in both *Piatnitzkysaurus* and *Condorraptor*; thus indicating the CFL insertion (level I) and exemplifying that it was a large muscle in early tetanurans (Figures 6 and 7). As proposed by Hutchinson (2001a) and Carrano and Hutchinson (2002), the secondary tendon of the CFL may have been lost in early theropods, as the fourth trochanter became less “pendant” (distally angled) than in many other archosaurs. Considering that the fourth trochanter of both studied taxa is well‐developed but not pendant, this secondary tendon would probably have been absent (level II').

#### 5.1.7 Digital extensor group

*M. tibialis anterior* (*TA*): The TA muscle (previously termed as *M*. *extensor digitorum longus* in non‐avian Reptilia; see Hattori & Tsuihiji, 2021) in Crocodylia (=*M*. *tibialis cranialis*, TC in Aves) originates from a narrow tendon proximal to the lateral femoral condyle, lateral to the extensor groove and distal to the large *M*. *femorotibialis* origins (Hattori & Tsuihiji, 2021; Hutchinson, 2002; Picasso, 2010; Suzuki et al., 2011). In Aves, the homologous muscle originates from the distal extremity of the lateral femoral condyle, from the *fovea tendinis m*. *tibialis cranialis* (Baumel & Witmer, 1993); a second TC head also originates from the lateral and cranial cnemial crests of the tibia, proximal to the *M. extensor digitorum longus* origin (Hattori & Tsuihiji, 2021; Hutchinson, 2002).

**FIGURE 15:**
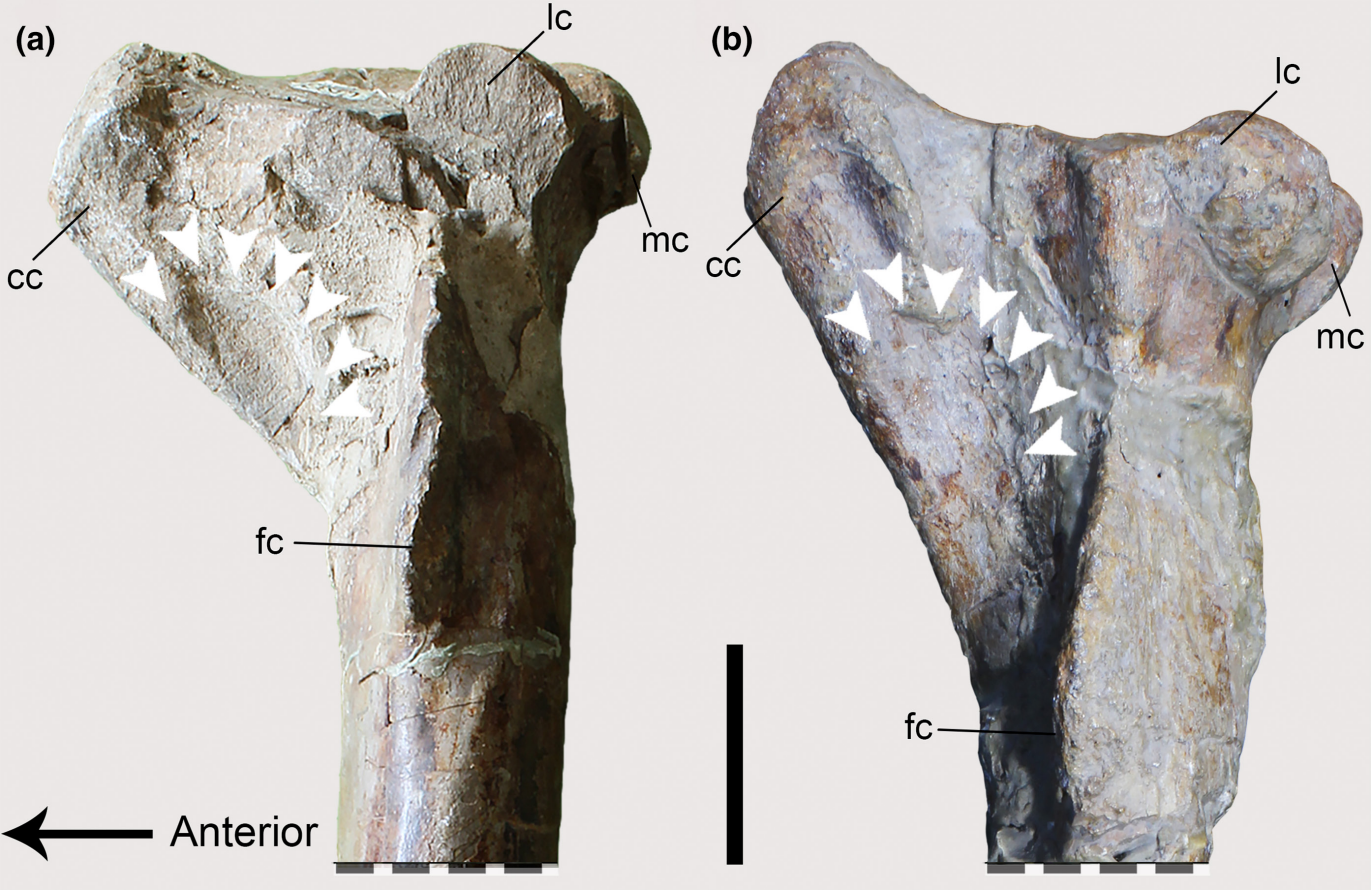
Proximal tibiae of *Piatnitzkysaurus* (left tibiae, lateral view). (a) PVL 4073 specimen. (b) MACN‐Pv‐CH 895 specimen. Anatomical/muscular abbreviations: cc, cnemial crest; fc, fibular crest; lc, lateral condyle, mc, medial condyle. Arrows indicate muscle scar. Scale bar = 50 mm.

Generally, reconstructions of the TA muscle origin in theropods consider both muscular heads, as aforementioned originating from the lateral condyle of the femur and the proximal tibia (e.g., Carrano & Hutchinson, 2002; Smith, 2021, 2023). In the femora of both *Piatnitzkysaurus* and *Condorraptor*, there is no evidence of the TA origin; the lateral condyles do not have the distally positioned *fovea tendinis m*. *tibialis cranialis* as in Aves (e.g., Baumel & Witmer, 1993; Hattori & Tsuihiji, 2021; Picasso, 2010) or proximal to the lateral condyle as in Crocodylia (e.g., Hattori & Tsuihiji, 2021; Suzuki et al., 2011). Therefore, the specific origin of this muscle head on the femur in both piatnitzkysaurids is ambiguous, but as the origin of this muscle is a conservative feature in Reptilia (Hattori & Tsuihiji, 2021), and probably present in theropods (Carrano & Hutchinson, 2002), we tentatively reconstruct this muscle on the anterior lateral condyle (level I′) (Figures 6 and 7). Considering the second TA head, the tibiae of both *Piatnitzkysaurus* specimens, MACN‐Pv‐CH 895 and PVL 4073, distal to the insertion of the *triceps femoris* on the cnemial crest, have an elliptical and well‐demarcated depression (Figure 15). This depression is topologically equivalent to the TA reconstruction in early theropods such as *Coelophysis* (Bishop et al., 2021) and later coelurosaurs such as *Tyrannosaurus* (Carrano & Hutchinson, 2002), *Nothronychus* (Smith, 2021), as well as Aves (e.g., Hattori & Tsuihiji, 2021); thus, it is considered here as the second head of TA origin (level I) (Figures 4a,b and 15). In the *Condorraptor* tibia MPEF‐PV 1672, this depression is not clearly noticeable (level I′) (Figure 4c,d).

The TA in non‐avian Reptilia inserts onto tubercles on the metatarsals, being distal in turtles and proximal in Lepidosauria and Crocodylia; in Aves this tendon splits and inserts onto the dorsal/anterior surface of the proximal tarsometatarsus on a tubercle (*tuberositas m*. *tibialis cranialis*) (Baumel & Witmer, 1993; Hattori & Tsuihiji, 2021; Hutchinson, 2002; Suzuki et al., 2011, 2014). These metatarsal tubercles or longitudinal crests (typically concentrated on metatarsal II but also III) are noted in other archosaurs and many dinosaur taxa (e.g., Carrano & Hutchinson, 2002; Hutchinson, 2002; Langer, 2003; Smith, 2021, 2023).

Similar to other dinosaurs (Carrano & Hutchinson, 2002; Dilkes, 2000; Langer, 2003; Smith, 2023), the anteroproximal metatarsal shafts in *Piatnitzkysaurus* specimen MACN‐Pv‐CH 895 have a longitudinal crest and a proximal excavation. We thus infer the proximal parts of metatarsals II–IV (mainly metatarsal II) as the TA insertions (level I). Only the left metatarsal IV (MPEF‐PV 1692) is preserved in *Condorraptor*; although it does not have the evident ridge present in *Piatnitzkysaurus*, the proximal portion preserves an excavation, considered here a TA insertion (level I).

*M. extensor digitorum longus* (*EDL*): The EDL (previously termed as *M*. *tibialis cranialis* in non‐avian Reptilia; see Hattori & Tsuihiji, 2021) in extant Reptilia originates from the proximal shaft of the tibia; in Crocodylia from a rugose surface in the proximalmost portion and in Aves from a broad surface located between the cranial and the lateral cnemial crests and distal to the insertion of the *triceps femoris* tendon (Hattori & Tsuihiji, 2021; Hutchinson, 2002; Suzuki et al., 2011, 2014).

In the tibiae of *Piatnitzkysaurus* PVL 4073 and *Condorraptor* MPEF‐PV 1672, a clear demarcation is visible on the anterolateral shaft, located in the sulcus intercnemialis. The proximal limits are not well‐defined and may reach the cnemial crest (as in Aves—Suzuki et al., 2014; Hattori & Tsuihiji, 2021), but the anterior limits are bordered by a muscular line, and the posterior limits proximally by the fibular crest and distally by a posterior muscular line. As noted for Aves (e.g., Hattori & Tsuihiji, 2021), the distal part of the EDL origin is tapered (level I) (Figure 4).

The EDL insertion in non‐avian Reptilia is limited to a bulge(s) on the dorsal surface of metatarsals I–II (Hattori & Tsuihiji, 2021; Hutchinson, 2002), whereas in Aves this insertion is on the proximal processes of the distal pedal phalanges, in the hyperextensor fossae (Hattori & Tsuihiji, 2021; Hutchinson, 2002; Picasso, 2010; Suzuki et al., 2014). The EDL insertion in early dinosaurs is inferred as a distally positioned when compared to non‐avian Reptilia, due to the presence of large extensor fossae and rugosities on the dorsal surfaces of the pedal phalanges (Bishop et al., 2021; Carrano & Hutchinson, 2002; Hutchinson, 2002; Smith, 2021). Although the condition in piatnitzkysaurids is probably the same as in other dinosaurs, this insertion has not been reconstructed as the specimens have no preserved phalanges (with the exception of an isolated ungual of *Condorraptor*).

*M. extensor digitorum brevis* (*EDB*): The EDB in Reptilia has its origin on the astragalus (or distal tarsal IV in Testudines) and ankle elements (with some variability existing in crocodylians and other taxa), inserting onto the dorsal surface of the pedal phalanges; however, in Aves, this muscle is absent (Dilkes, 2000; Hattori & Tsuihiji, 2021; Hutchinson, 2002). The EDB is conjectured to have fused with the EDL in dinosaurs (Carrano & Hutchinson, 2002; Dilkes, 2000). We did not reconstruct this muscle following this hypothesis (a level II' reconstruction) as such elements are not preserved in piatnitzkysaurids.

*M. extensor hallucis longus* (*EHL*): The EHL (also termed *M*. *flexor perforatus digiti II*; homologue to the *M*. *extensor hallucis brevis* of crocodilians—Hattori & Tsuihiji, 2021) in non‐archosaurian Reptilia is conservative in position. being a small and short muscle originating from the distal shaft of the fibula, inserted onto the hallucal phalanges (Hattori & Tsuihiji, 2021; Hutchinson, 2002). In Aves, related to loss of the distal fibula, the EHL muscle origin has moved distally to the anteromedial portion of the proximal tarsometatarsus (Hutchinson, 2002; Moreno, 1990; Patak & Baldwin, 1998), or the EHL is absent in species that have lost the hallux (e.g., Suzuki et al., 2014).

In non‐avian theropods (e.g., Bishop et al., 2021; Carrano & Hutchinson, 2002; Smith, 2023), including *Piatnitzkysaurus* specimen PVL 4073, the fibula is not distally reduced as in Aves and some other theropods (e.g., Smith, 2021) and thus represented the EHL origin, distal to *Mm. fibulares longus et brevis* (FL, FB) (level I). *Condorraptor* and *Marshosaurus* do not have a fibula preserved.

The EHL muscle insertion is onto the anterior portion of the hallucal phalanges in Reptilia; whereas in Aves it becomes more posterior due to changes in hallux position (Hutchinson, 2002). Reconstructions of this muscle in non‐avian theropods consider this insertion as onto the anterior side of the hallucal ungual (Bishop et al., 2021; Carrano & Hutchinson, 2002; Smith, 2023). The lack of the well‐preserved pes in piatnitzkysaurids precludes any inferences about this muscular insertion.

#### 5.1.8 Digital flexor group

*M. gastrocnemius pars lateralis* (*GL*): In Lepidosauria and extant archosaurs, the GL (variably named; =*M*. *gastrocnemius externus*, GE in Crocodylia) is a large and fusiform muscle with a fleshy origin from the posterodistal surface of the femur, distal to ADD2 (Hattori & Tsuihiji, 2021; Hutchinson, 2002; Mckitrick, 1991; Otero et al., 2010; Patak & Baldwin, 1998; Picasso, 2010; Romer, 1923a). In Crocodylia and Aves, this muscle originates from a lateroventral distinct depression on the posterior portion of the distal femur. In Aves, it is delimited by a rough depression and has deep and superficial layers, sometimes with an extra lateral muscle head (e.g., Hattori & Tsuihiji, 2021; Suzuki et al., 2011).

Both femora of *Piatnitzkysaurus* PVL 4073 preserve a depression on the posterolateralmost part of the distal femoral shaft; the right femur has some degree of rugosity on the lateral base of the tibiofibular crest. This posterolateral depression is topologically located in a position similar to extant Reptilia; thus interpreted here as the GL origin (level I) (Figure 6g,h). A depression similar in position and shape is present on the *Condorraptor* left femur MPEF‐PV 1690, allowing reconstruction of the GL origin (level I) (Figure 7g,h).

In extant archosaurs, the GL and *M*. *gastrocnemius medialis* (GM) muscles or their homologues insert via a shared tendon (and aponeurosis/plantar fascia) to metatarsal V (plantar surface of pes) in Crocodylia, and the medial and plantar margins of the hypotarsus of Aves (Hattori & Tsuihiji, 2021; Hutchinson, 2002; Mckitrick, 1991; Otero et al., 2010; Picasso, 2010; Romer, 1923a). It is thought that the plantar aponeurosis was reduced in dinosaurs (see Hutchinson, 2002), yet with the GM + GL maintaining robust scars on the posterior metatarsal shafts (Bishop et al., 2021; Carrano & Hutchinson, 2002; Dilkes, 2000; Hutchinson, 2002; Smith, 2021).

**FIGURE 16:**
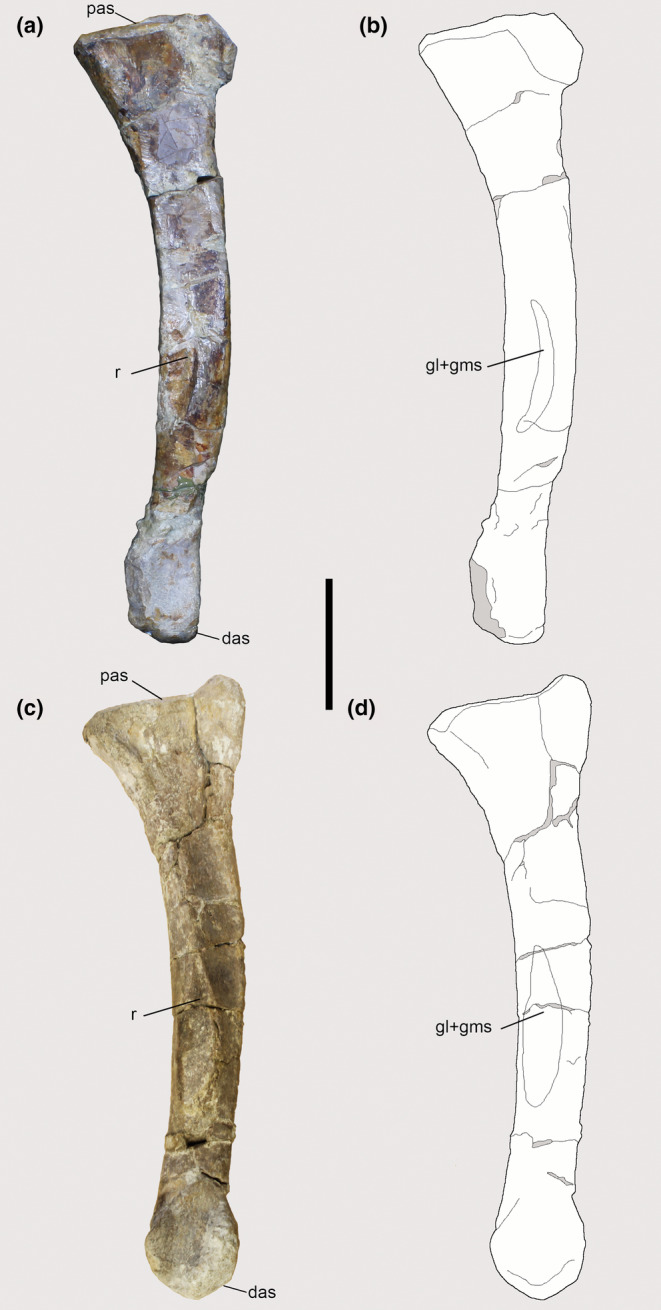
Metatarsal IV of Piatnitzkysauridae (left, posterior view). (a, b) *Piatnitzkysaurus* (MACN‐Pv‐CH 895). (c, d) *Condorraptor* (MPEF‐PV 1692). Anatomical/muscular abbreviations: das, distal articular surface; gl + gms, *Mm*. *gastrocnemii* insertion scar; pas, proximal articular surface; r, ridge. Scale bar = 50 mm.

The left metatarsals II–IV in the *Piatnitzkysaurus* MACN‐Pv‐CH 895 specimen are well‐preserved. The metatarsal of *Piatnitzkysaurus* has a ridge on the posterior/plantar surface related to the insertion of GL + GM (level II) (Figure 16a,b), although not so prominent as in other theropods (e.g., *Majungasaurus*—Carrano, 2007; *Skorpiovenator*—Cerroni et al., 2022; *Tyrannosaurus*—Carrano & Hutchinson, 2002). The left metatarsal IV (MPEF‐PV 1692) of *Condorraptor*, as previously noted by Rauhut (2005) has a posterolateral semilunate ridge on the posterior/plantar surface (Figure 16c,d), which is the insertion site of the GL + GM heads (level I), presumably also inserting onto metatarsals II and III; not preserved (level I′).

*M. gastrocnemius pars medialis* (*GM*): In Reptilia, the GM muscle (=*M. gastrocnemius internus*, GI in Crocodylia) originates from the medial surface of the proximal tibia, occupying a large area on the cnemial crest in Aves (Hattori & Tsuihiji, 2021; Hutchinson, 2002; Otero et al., 2010; Patak & Baldwin, 1998; Picasso, 2010; Romer, 1942; Suzuki et al., 2011, 2014). Although *Mm*. *gastrocnemii* is composed of the lateral and medial head ancestrally in Reptilia, lepidosaurs, and Aves evolved a third head independently (*M*. *gastrocnemius pars intermedia* in Aves), the third head in lepidosaurs probably deriving from a subdivision of the lateral head and in Aves deriving from a subdivision of the medial head. At least in the lineage of Aves, the timing of the derivation of this extra head is difficult to determine (Hutchinson, 2002).

A large depression is on the medial surface of the proximal tibia on the tibiae of both *Piatnitzkysaurus* specimens, MACN‐Pv‐CH 895 and PVL 4073, covering almost the entire cnemial crest (except the anteroproximalmost part where the *triceps femoris* tendon should have attached) (Figure 12a–d). Although this broad depression exists in both specimens, in the PVL 4073 tibia, a ridge subdivides this depression into two subconcavities (Figure 12a,b). It is not clear whether these subdivisions signify an “extra head” of the GM (as reported in Crocodylia, which originates from the *triceps femoris* tendon—Suzuki et al., 2011). Regardless, the origin of the GM muscle seems to have been in this position. The GM origin reconstructed here in *Piatnitzkysaurus* is the entire medial depression on the cnemial crest (level I), resembling the large area of GM origin in Aves (Figure 12a–d). Likewise, the medial side of the cnemial crest in the *Condorraptor* tibia MPEF‐PV 1672 also has a broad and shallow depression, positioned distally in relation to the *triceps femoris* tendon, representing the GM origin (level I) (Figure 12e,f). The insertion site of GL + GM was described above (Figure 16c,d).

*Mm. fibulares longus et brevis* (*FL, FB*): The FL and FB origins (also termed *M*. *peroneus longus et brevis* and *Mm. peronei anterior et posterior*) in Testudines, Lepidosauria and extant archosaurs are from the fibula, in some cases with contribution from the tibia (Dick & Clemente, 2016; Dilkes, 2000; Hattori & Tsuihiji, 2021; Hutchinson, 2002). Generally, the FL origin is on the lateral fibula, distal to the ILFB insertion; whereas the FB is more distally and anterolaterally positioned on the fibula (Hattori & Tsuihiji, 2021; Hutchinson, 2002; Suzuki et al., 2011, 2014). With the distal region of the fibula lost in early Aves, the origin of *Mm*. *fibulares* became superficial on the lateral sides of the proximal tibiotarsus and fibula (Hutchinson, 2002; Patak & Baldwin, 1998; Picasso, 2010).

Similar to other dinosauriform reconstructions (e.g., Bishop et al., 2021; Carrano & Hutchinson, 2002; Dilkes, 2000; Piechowski & Tałanda, 2020; Smith, 2021), the presence of a distally unreduced fibula suggests that the FL and FB origins in *Piatnitzkysaurus*, were mainly from the middle to distal fibular shaft. Although there is no clear demarcation or muscle scarring, we reconstructed these muscles (level I′) based on the PVL 4073 fibula.

In general, ancestral Reptilia have the FL and FB insertions on the proximolateral tarsals, metatarsal V, and 5th digit aponeurosis; and near the proximal end of metatarsal V, respectively (Hattori & Tsuihiji, 2021; Hutchinson, 2002). Some modifications occurred in the avian lineage, especially the reduction/loss of the plantar aponeurosis and the 5th digit, concentrating these muscular insertions onto the lateroproximal side of the tarsometatarsus in Aves (Hattori & Tsuihiji, 2021; Hutchinson, 2002). Reconstructions of these insertions in Dinosauriformes usually are onto the tarsal/metatarsal elements, particularly metatarsal V (Bishop et al., 2021; Carrano & Hutchinson, 2002; Dilkes, 2000; Piechowski & Tałanda, 2020; Smith, 2021). The lack of preserved tarsals and metatarsal V in piatnitzkysaurids prevents the reconstruction of the FL and FB insertions in detail, but presumably, they were the same as in other non‐avian Dinosauriformes.

#### 5.1.9 Summary of muscle reconstructions

**FIGURE 17:**
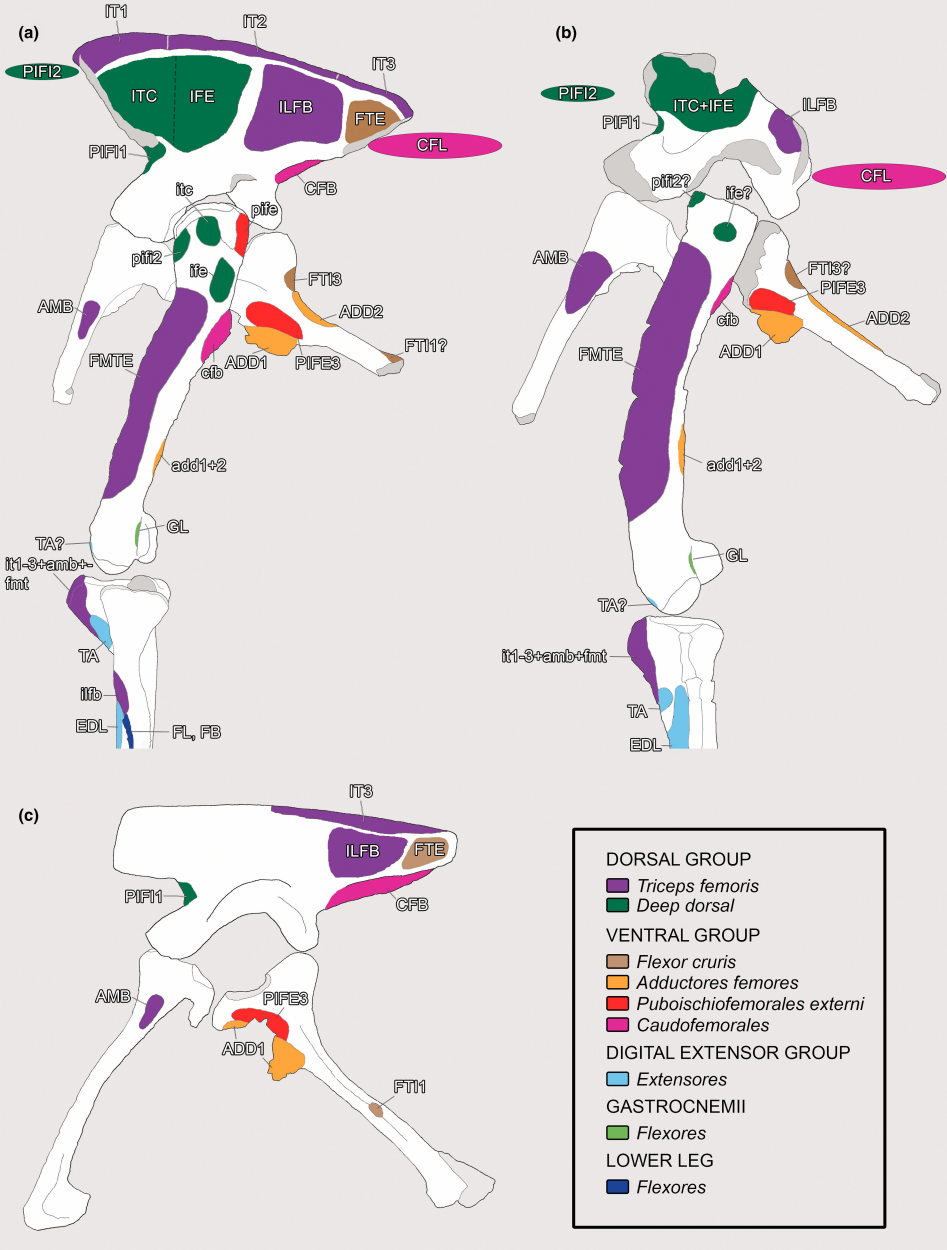
Pelvic and hindlimb ‘muscle map’ inferred for Piatnitzkysauridae (left lateral view). (a) *Piatnitzkysaurus floresi*. (b) *Condorraptor currumili*. (c) *Marshosaurus bicentesimus*. Note that some muscles are not shown here, and some bones have been mirrored to illustrate the reconstructions of the three piatnitzkysaurid species. Muscle abbreviations are provided in Table 1; see text for inference levels and other comparisons. CFL and PIFI2 origins are much smaller than expected; simply shown for relative positions. Medial muscle origins (e.g., FMTI) and insertions (e.g., PIFI1) are not shown. Mm. gastrocnemii are labelled as “Flexores” due to their action around the knee; lower leg (FB, FL, etc.) as “Flexores” for ankle dorsiflexion. Not to scale.

The muscle reconstructions inferred for *Piatnitzkysaurus*, *Condorraptor*, and *Marshosaurus* are summarized in Tables 2, 3, 4, respectively. Figure 17 presents a “muscle map” reconstruction for each of the studied species. Overall, we infer 29 muscles' origins for *Piatnitzkysaurus*, which is the best‐preserved specimen; and among these 29 muscles, it was possible to infer the insertions of 25 (Figure 17a). In *Condorraptor*, 21 muscles were reconstructed; among these, 12 were inferred for both origin, and insertion (Figure 17b). *Marshosaurus* is the specimen with the fewest pelvic elements preserved, rendering it possible to infer only 12 muscles (Figure 17c), and only the origins are inferred here because no stylopodium and zeugopodium are known for this taxon.

**TABLE 2:** Pelvic and hindlimb musculature inferred for *Piatnitzkysaurus*, and required inference level based on the EPB. Refer to Table 1 or the main text results for muscle abbreviations.

Muscle	Origin	Insertion
IT1	Anterodorsal rim of the lateral ilium (I), in a rough and dorsoventrally delimited area	Tibial cnemial crest (I)
IT2	Dorsal rim of the ilium (I); anterior limits over the horizontal axis of the pubic peduncle, posterior limit over the horizontal axis of the posterior facet of the ischial peduncle	Tibial cnemial crest (I)
IT3	Posterodorsal rim of the ilium (I); posterior to the IT2, in the posterodorsal end of the postacetabular ilium	Tibial cnemial crest (I)
AMB	Pubic tubercle (I), on the lateral shaft of the pubis	Tibial cnemial crest (I)
FMTE	Lateral surface of the femoral shaft, delimited by the *lia* and *lip* (I)	Tibial cnemial crest (I)
FMTI	Anteromedial surface of the femoral shaft, delimited by *lia* and *la* (I)	Tibial cnemial crest (I)
ILFB	Shallow depression on the postacetabular surface of the ilium, ventral to IT3 (I)	Fibular tubercle (I)
IFE	Elliptical concavity on the dorsolateral surface of the ilium (I); posterior to ITC and ventral to IT2 (II)	Femoral trochanteric shelf (II)
ITC	Elliptical concavity on the lateral surface of the ilium (I), anterior to IFE (II)	Lesser trochanter (anterior) of the femur (II)
PIFI1	Preacetabular ventrolateral ‘cuppedicus’ fossa (I)	Anteromedial surface of the femur, distal to the lesser trochanter (I)
PIFI2	Centra of vertebrae anterior to ilium (I), and potentially near PIFI1 on ilium (I′)	Anterolateral surface of the femur, distal to the lesser trochanter (‘accessory trochanter’) (I)
FTI1	Distal ischial tubercle (II)	Proximal posteromedial surface of the tibia in a broad depression (II)
FTI2	Equivocal (II'); not reconstructed (possible autapomorphy of Crocodylia)	Equivocal (II')
FTI3	Proximal ischial tuberosity (II)	Posteromedial surface of the proximal tibia (I)
FTI4	Equivocal (II'); not reconstructed (possible autapomorphy of Crocodylia)	Equivocal (II')
FTE	Postacetabular blade; posterior to the ILFB (I)	Posteromedial surface of the proximal tibia (I)
ADD1	Obturator process of the ischium (ischial apron) (I′)	Posterior shaft of the femoral diaphysis (I′)
ADD2	Depression on the posterodorsal ischial shaft, slightly distal to the ischial tuberosity (II)	Posterior shaft of the femoral diaphysis (I′)
PIFE1	Anterior surface of the pubic apron (II)	Femoral greater trochanter (I)
PIFE2	Posterior surface of the pubic apron (II)	Femoral greater trochanter (I)
PIFE3	Obturator process of the ischium; between ADD1 and ADD2 (II)	Femoral greater trochanter (I)
ISTR	Medial surface of ischium/obturator process (II)	Posterolateral side of the proximal femur, between the greater and fourth trochanter (I)
CFB	Iliac brevis fossa (II)	Lateral surface of the fourth trochanter (I)
CFL	Centra and haemal arches of the caudal vertebrae (I), continuing distally until the transverse processes are strongly reduced/absent (I′)	Pit and crest of the medial to posterior surface of the fourth trochanter (I)
EDL	Anterolateral proximal shaft of the tibia (I)	?
EHL	Anterolateral surface of distal fibula (I)	?
GL	Depression on the posterolateral surface of the distal femoral shaft (I)	Posterior/plantar surfaces of metatarsals II–IV (I)
GM	Depression on the anteromedial proximal tibia (I)	Posterior/plantar surfaces of metatarsals II–IV (I)
TA	Anterolateral proximal side of femoral condyle (I′) and/or depression distal to the cnemial crest of the tibia (I)	Anteroproximal metatarsals II–IV (I)
FL	Anterolateral fibula (I′)	?
FB	Anterolateral fibula, distal to FL (I′)	?

#### 5.1.10 Ambiguous reconstructions and unknown character states in Piatnitzkysaurus

Table 5 summarizes the hypothesized character states for the most recent common ancestor of Tetanurae (i.e., Orionides + Coelurosauria; Carrano et al., 2012; Gauthier, 1986), based on our maximum parsimony state reconstructions. The following characters have been mapped as unknown states (?) in *Piatnitzkysaurus*: 8–9, 11, 14, 24, 27, 30–34, 37–40, 42–46, 49, 64, 69–70, 72–79, 81–82, 85–100. This uncertainty was due to the lack of osteological correlates in this taxon that could clarify myological issues previously discussed in Hutchinson (2002) and Bishop et al. (2021).

On the contrary, the following muscles were inferred as absent in this taxon: (1) *M*. *puboischiotibialis* (PIT), which is present in non‐avian Reptilia, arising from a muscle scar on the anterolateral ilium (Hutchinson, 2002; Otero et al., 2010; Suzuki et al., 2011) that is absent in Avialae (Hutchinson, 2002); and (2) *M*. *pubotibialis* (PUT), which originates from the pubis, near the pubic tubercle and puboischiadic ligament, in some Reptilia (Hutchinson, 2002; Romer, 1942) but was lost in Archosauria (Bishop et al., 2021; Dilkes, 2000; Hutchinson, 2002; Romer, 1923a). Other muscles such as FTI2 and 4, GIM, and EDB (probably fused with EDL) are equivocal and were not reconstructed; thus, their presence or absence was not inferred (see Table 2 for *Piatnitzkysaurus* reconstruction; Hutchinson, 2002 and Bishop et al., 2021 for further discussions).

### 5.2 Myological comparisons among theropods

Here we have associated morphological structures with the myology of the locomotor apparatus in piatnitzkysaurids, combining our work with previous descriptions (Bonaparte, 1979, 1986; Madsen, 1976; Rauhut, 2005). This is the first detailed myological study of the pelvic appendages in early Tetanurae, as far as we know. While the reconstructions we found are similar to others performed for different theropods, we present some key comparisons here.

**FIGURE 18:**
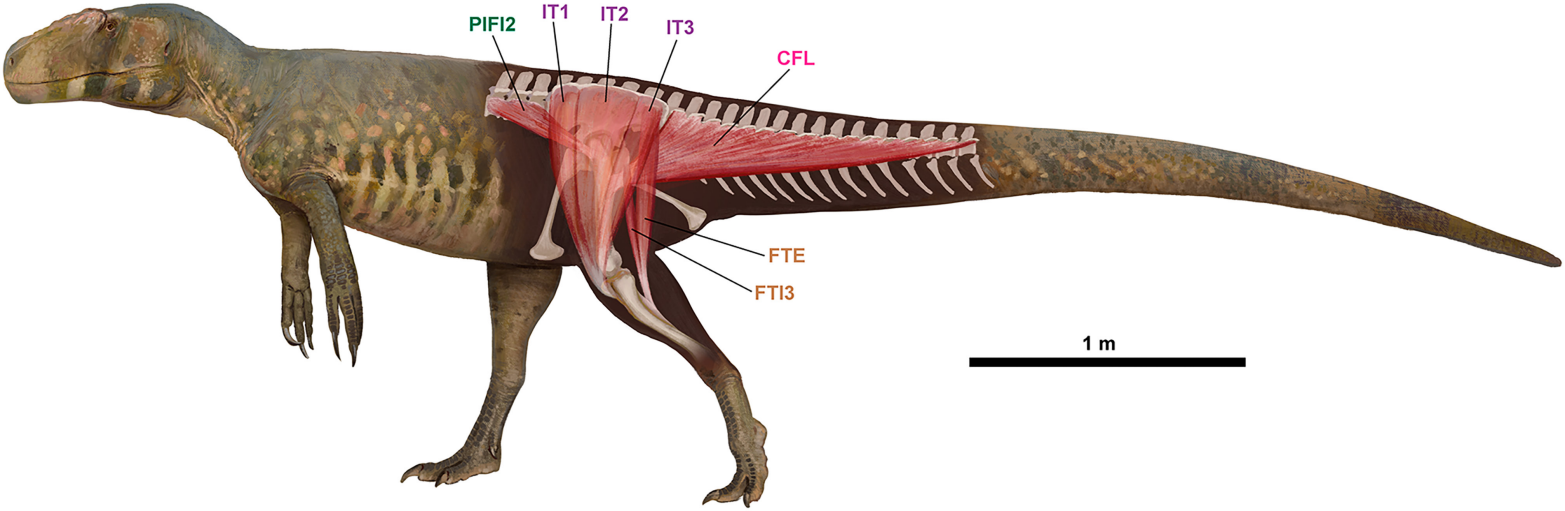
Restoration of pelvic and hindlimb muscles in *Piatnitzkysaurus floresi* (left lateral view). Artwork by Júlia d'Oliveira.

Earlier reconstructions of theropod myology generally considered the superficial IT muscle as a single component of the thigh (e.g., Romer, 1923a; Russell, 1972; Tarsitano, 1983), whereas more recent reconstructions have considered three heads of this muscle (e.g., Carrano & Hutchinson, 2002; Grillo & Azevedo, 2011; Rhodes et al., 2021), which applies to our reconstruction based on *Piatnitzkysaurus* (Figures 17 and 18).

The division of the IF of non‐avian Reptilia into the ITC + IFE of Aves is ubiquitous in recent pelvic musculature reconstructions of theropods (e.g., Bates, Benson, & Falkingham, 2012; Bishop et al., 2021; Carrano & Hutchinson, 2002; Hutchinson & Gatesy, 2000), based mainly on inferred insertions of these muscles, and we infer the same division in piatnitzkysaurids (mainly *Piatnitzkysaurus*; Figures 2 and 17). However, similar to *Staurikosaurus* (Grillo & Azevedo, 2011), it is not possible to distinguish the origins of these two muscles, except that, based on the EPB (Figure 1a), that the ITC was anteriorly positioned and IFE immediately posterior (see Hutchinson, 2002). This reconstruction, like others, differs from the *Falcarius* reconstruction figured in Smith (2023), in which the IFE was positioned posteriorly on the ilium (between ILFB and FTE); a condition not known in Aves.

The insertion of ITC in *Piatnitzkysaurus*, similar to Ceratosauria, Allosauroidea, Tyrannosauroidea, and Ornithomimosauria, occurred onto a large “blade‐like” lesser trochanter of the femur (Figures 6 and 9), differing from other dinosauriformes and early theropods (e.g., Herrerasauridae and *Coelophysis*), in which the ITC insertion was onto a small “knob‐like” lesser trochanter (Bishop et al., 2021; Carrano & Hutchinson, 2002; Grillo & Azevedo, 2011; Hutchinson, 2002; Lacerda et al., 2023). Later‐diverging theropods (e.g., Oviraptorosauria) had this insertion on a more robust and proximally positioned lesser trochanter, whereas Aves have only a scar on the trochanteric crest (Bishop et al., 2021; Hutchinson, 2002), thus indicating a “transitional” position in *Piatnitzkysaurus* between early‐diverging theropods and later‐diverging coelurosaurians.

In early theropods such as *Staurikosaurus* (Grillo & Azevedo, 2011), *Coelophysis* (Bishop et al., 2021), the allosauroids *Allosaurus* and *Acrocanthosaurus* (Bates, Benson, & Falkingham, 2012), and later coelurosaurians such as *Tyrannosaurus* (Carrano & Hutchinson, 2002), *Nothronychus* (Smith, 2021), and *Falcarius* (Smith, 2023), the AMB origin usually is reconstructed more anteroproximally on the pubis. However, in our reconstructions, based on the position of the pubic tubercle, the AMB origin in piatnitzkysaurids appears to have been more laterodistal (Figures 5 and 17). Similarly, a slightly more distal AMB is suggested for *Albertosaurus* (Rhodes et al., 2021) and even more distally in the dinosauriform *Silesaurus* (Piechowski & Tałanda, 2020).

Based on a well‐developed accessory trochanter (Figure 9), our inferred insertion of PIFI2 on the proximal part of the femur in *Piatnitzkysaurus* is slightly more anteriorly positioned (Figures 6, 9 and 17) than in *Tyrannosaurus* (Carrano & Hutchinson, 2002) and *Falcarius* (Smith, 2023), being more similar to the reconstructed insertion in early theropods (e.g., Bishop et al., 2021) and allosauroids (Bates, Benson, & Falkingham, 2012).

In early theropods such as *Staurikosaurus* (Grillo & Azevedo, 2011) and allosauroids (Bates, Benson, & Falkingham, 2012), the inferred ADD2 origin is restricted to the most posterior part of the ischial shaft, whereas in *Coelophysis* (Bishop et al., 2021), this muscle was reconstructed in a slightly more distal position. In our reconstruction of piatnitzkysaurids (mainly in *Condorraptor*; Figures 11 and 17), the ADD2 origin extends more distally, similar to Crocodylia (e.g., Suzuki et al., 2011) and reconstructions of some other theropods (e.g., Carrano & Hutchinson, 2002; Rhodes et al., 2021). Some of these differences might relate to subjective interpretations of the ADD2 scar location, but as Hutchinson (2001b) showed, this scar is fairly conservative and conspicuous in archosaurs.

As in Crocodylia (e.g., Otero et al., 2010; Romer, 1923b; Suzuki et al., 2011) as well as *Staurikosaurus* (Grillo & Azevedo, 2011) and *Coelophysis* (Bishop et al., 2021), we reconstructed the PIFE3 origin on the lateral aspect of the obturator process of the ischium, between the ADD1 + 2 (Figures 11 and 17). This origin extends more posteriorly in Crocodylia, but the anterior region lies between ADD1 + 2. In a *Tyrannosaurus* reconstruction (Carrano & Hutchinson, 2002), the PIFE3 originates slightly distal to ADD1, and is even more distally positioned in some maniraptoran reconstructions (Rhodes et al., 2021). These differences likely relate not only to subjective interpretations, but also to relative position of the ischial obturator process (Hutchinson, 2001b).

Our reconstruction of the ISTR origin differs from other myological studies'. In general, reconstructions in theropods consider the ISTR origin as dorsal and occupying a small medial part of the ischium (e.g., Carrano & Hutchinson, 2002; Grillo & Azevedo, 2011; Smith, 2021). However, here we consider the condition in Crocodylia and other non‐avian Reptilia (e.g., Romer, 1923a; Suzuki et al., 2011) to be more plausible, reconstructing the ISTR originating entirely on the medial surface of the ischium, probably occupying the entire area of the obturator process (similar to that hypothesized for *Coelophysis*; Bishop et al., 2021).

In theropod reconstructions, in general, the GM origin is on the medial portion of the proximal tibia. However, it variably is reconstructed somewhat distally, either more anteromedially (e.g., *Coelophysis*; Bishop et al., 2021), posteromedially (e.g., *Tyrannosaurus*; Carrano & Hutchinson, 2002), or anteriorly (e.g., *Falcarius*; Smith, 2023). Our reconstruction of the GM origin, based on the medial concavities on the cnemial crest (Figure 12), more closely resembles the condition in Aves, of a more anterior and proximal origin occupying the entire medial side of the cnemial crest, immediately distal to the *triceps femoris* tendon (e.g., Suzuki et al., 2014). This finding is cause to reinvestigate the GM origin in other theropods (e.g., Carrano & Hutchinson, 2002).

Overall, as in other recent studies of earlier (e.g., Grillo & Azevedo, 2011) and later (e.g., Carrano & Hutchinson, 2002) theropods, we infer that the myology of the pelvic appendage more closely resembled that of Aves than Crocodylia, thus characterizing the evolution of locomotor musculature in the avian lineage (e.g., Hutchinson, 2001a, 2001b, 2002). There seems to have been much conservatism across non‐avian Theropoda until Maniraptora. For example, many theropods (and some other Dinosauriformes) are inferred to have had a division of IF into IFE and ITC, an origin of PIFI1 (and possibly some of PIFI2) from the “cuppedicus fossa,” a CFB origin largely from the “brevis fossa,” a fused EDL and EDB, and the putative absence of some apomorphic muscles of Crocodylia such as FTI4 and a second AMB head; or loss of plesiomorphic muscles such as FTI2 and PIT (e.g., Hutchinson, 2002). Some features inferred for piatnitzkysaurids, such as the tibial *triceps femoris* insertion, three IT heads and two FMT heads, ILFB insertion onto the fibular tubercle, two ADD heads, and insertions of the PIFI1 + 2, FTI3, FTE, ADD1 + 2, PIFEs, ISTR, CFB and CFL are plesiomorphic muscular conditions (for Archosauria, Reptilia or earlier) that are relatively conservative even through evolution to Aves. Others evolved later on the avian stem, such as loss of the FTI1 and PIFE3, and shifts of the PIFI1 + 2, CFB, and ISTR origins from more medial to lateral.

Lower limb muscle origins and insertions have more complex, and sometimes more ambiguous, evolutionary patterns, evident in piatnitzkysaurids. However, the insertions of *Mm*. *gastrocnemii* and *M*. *extensor digitorum longus* (although not reconstructed here, but piatnitzkysaurids probably shared a similar insertion to other theropods) moved distally, relative to crocodylians. This presumed change is related to the evolutionary transformations on the lineage to Aves/Neornithes (and bipedal locomotion).

### 5.3 Myological comparisons among piatnitzkysaurids

As a consequence of osteological similarities, the myology of the pelvic girdle in these taxa presents several similarities (Figure 17). However, we highlight five differences here:

1. Area of CFB origin in *Piatnitzkysaurus* and *Marshosaurus*—the posteriorly wider brevis fossa in *Marshosaurus* (Carrano et al., 2012; Lacerda et al., 2023; Figures 12, 14, 17), may signal a larger CFB in *Marshosaurus* (likely greater force‐generating potential; e.g., Cuff, Demuth, et al., 2023; Cuff, Wiseman, et al., 2023), and perhaps different CFB moment arms about the hip joint (e.g., Allen et al., 2021).
2. Position and area of AMB origin among the three piatnitzkysaurids—the pubic tubercle is well‐developed in piatnitzkysaurids; even more robust in *Condorraptor* (Rauhut, 2005). Thus, in our muscle reconstructions, the AMB origin appears to have occupied a larger area in *Condorraptor* (and perhaps greater force potential), followed by *Piatnitzkysaurus*, with the origin also more distally positioned in both (Figures 5 and 17). Consequently, the AMB moment arms about the hip joint (e.g., Allen et al., 2021) should have varied among piatnitzkysaurids.
3. Extent of ADD1 origin in *Piatnitzkysaurus* and *Condorraptor*—the shallow depression present on the ischium of both taxa (not observed in *Marshosaurus*), extends more distally in *Condorraptor* (Figures 11 and 17), and again these differences could change the maximal forces generated and moment arms about the hip joint (e.g., Allen et al., 2021).
4. *Triceps femoris* tendon in *Piatnitzkysaurus* and *Condorraptor*—the cnemial crest in *Condorraptor* is only moderately developed (Figures 4b,c and 12e,f), differing from *Piatnitzkysaurus*, which has a well‐developed and nearly rectangular crest (Figures 4a,b, 12a–d and 15). Although the cnemial crest is moderately developed, it is an autapomophic feature in *Condorraptor* (Rauhut, 2005), and it implies a reduction of the *triceps femoris* insertion (Figure 17).
5. TA origin in *Piatnitzkysaurus* and *Condorraptor*—the second TA head (tibial) appears to have been more robust in *Piatnitzkysaurus* (Figures 5a–d and 15); perhaps indicating greater force‐generating capacity. However, considering that the *Condorraptor* specimen is a sub‐adult individual (Rauhut, 2005), this difference might be an ontogenetic feature.

### 5.4 Taphonomic limitations

The degree of preservation of the pelvic appendage varies in each of the three species studied here. *Piatnitzkysaurus* represents the best‐preserved specimen. This better preservation led to the larger number of successful inferences noted above (Table 2; Figures 2, 3, 4, 5, 6, 8, 9, 10, 11, 12, 14, 15, 16, 17). Furthermore, two individuals of *Piatnitzkysaurus* are known (Bonaparte, 1979, 1986), allowing comparisons between individuals (e.g., Figures 12 and 15), thus increasing the reliability of our muscular reconstructions for this taxon. However, the absence of most of the pedal bones prevents the analysis of many lower limb muscles. An illustration of *Piatnitzkysaurus* in Figure 18 summarizes the most superficial thigh muscles.

In taphonomic terms, *Condorraptor* has the second best‐preserved piatnitzkysaurid pelvic appendages, although only one skeleton is known, probably from the same individual (Rauhut, 2005). The lack of preservation of many distal hindlimb elements such as the fibula and pes, as well as the fragmentary state of the femora of this taxon (compare Figures 6 and 7) caused the weaker inferences (Table 3; Figures 2, 4, 5, 7, 11, 12, 13, 16, 17), especially for muscle insertions.

**TABLE 3:** Pelvic and hindlimb musculature inferred as present in *Condorraptor* and required inference level based on the EPB. Refer to Table 1 or the main text results for muscle abbreviations.

Muscle	Origin	Insertion
IT2	Dorsal rim of the ilium (I); anterior limits over the horizontal axis of the pubic peduncle	Tibial cnemial crest (I)
AMB	Pubic tubercle (I), on the lateral shaft of the pubis	Tibial cnemial crest (I)
FMTE	Lateral surface of the femoral shaft, delimited by the *lia* and *lip* (I)	Tibial cnemial crest (I)
FMTI	Anteromedial surface of the femoral shaft, delimited by *lia* and *la* (I)	Tibial cnemial crest (I)
ILFB	Shallow fragmentary depression on the postacetabular surface of the ilium (I)	?
IFE	Fragmentary concavity on the dorsolateral surface of the ilium (I); posterior to ITC and ventral to IT2 (II)	Femoral trochanteric shelf? (II)
ITC	Fragmentary concavity on the lateral surface of the ilium (I), anterior to IFE (II)	Lesser trochanter (anterior) of the femur? (II)
PIFI1	Preacetabular ventrolateral ‘cuppedicus’ fossa (I)	?
PIFI2	Centra of vertebrae anterior to ilium (I), and potentially near PIFI1 on ilium (I′)	?
FTI3	Proximal ischial tuberosity? (II)	?
ADD1	Obturator process of the ischium (ischial apron) (I′)	Posterior shaft of the femoral diaphysis (I′)
ADD2	Depression on the posterodorsal ischial shaft, slightly distal to the ischial tuberosity (II)	Posterior shaft of the femoral diaphysis (I′)
PIFE1	Anterior surface of the pubic apron (II)	?
PIFE2	Posterior surface of the pubic apron (II)	?
PIFE3	Obturator process of the ischium; between ADD1 and FTI3? + ADD2 (II)	?
ISTR	Medial surface of ischium/obturator process (II')	?
CFL	Centra and haemal arches of the caudal vertebrae (I), continuing distally until the transverse processes are strongly reduced/absent (I′)	Pit and crest of the medial to posterior surface of the fourth trochanter (I)
EDL	Anterolateral proximal shaft of the tibia (I)	?
GL	Depression on the posterolateral surface of the distal femoral shaft (I)	Posterior/plantar surface of metatarsals II–IV (I)
GM	Depression on the anteromedial proximal tibia (I)	Posterior/plantar surface of metatarsals II–IV (I)
TA	Anterolateral proximal side of femoral condyle (II') and/or depression distal to the cnemial crest of the tibia (II)	Anteroproximal metatarsals II–IV (I)

Although *Marshosaurus* is a better‐known taxon in terms of the number of bones, which includes the cranium (Carrano et al., 2012; Madsen, 1976), it has fewer preserved pelvic appendage elements than other piatnitzkysaurids, which prevents a more robust evaluation of the musculature in this species; only the muscle origins (Table 4; Figures 2, 3, 5, 11, 14, 17). Description of new material (e.g., Chure et al., 1997) should provide additional clues on the locomotor musculature.

**TABLE 4:** Pelvic and hindlimb musculature inferred as present in *Marshosaurus* and required inference level based on the EPB. Refer to Table 1 or the main text results for muscle abbreviations.

Muscle	Origin	Insertion
IT3	Posterodorsal rim of the ilium (I); posterior to the IT2, in the posterodorsal end of the postacetabular ilium	?
AMB	Pubic tubercle (I), on the lateral shaft of the pubis	?
ILFB	Shallow depression on the postacetabular surface of the ilium, ventral to IT3 (I)	?
PIFI1	Preacetabular ventrolateral ‘cuppedicus’ fossa (I)	?
FTI1	Distal ischial tubercle (II)	?
FTI2	Equivocal (II'); not reconstructed (possible autapomorphy of Crocodylia)	Equivocal (II')
FTI4	Equivocal (II'); not reconstructed (possible autapomorphy of Crocodylia)	Equivocal (II')
FTE	Postacetabular blade; posterior to the ILFB (I)	?
ADD1	Obturator process of the ischium (ischial apron) (I′)	?
PIFE1	Anterior surface of the pubic apron (II)	?
PIFE2	Posterior surface of the pubic apron (II)	?
PIFE3	Obturator process of the ischium; between ADD1 and ADD2 (II)	?
ISTR	Medial surface of ischium/obturator process (II)	?
CFB	Iliac brevis fossa (II)	?

**TABLE 5:** Reconstruction of character states for the Tetanurae node after scoring *Piatnitzkysaurus floresi*. States 01 and 012 represent ambiguous reconstructions.

Character	1	2	3	4	5	6	7	8	9	10
State	1	0	1	0	1	1	1	0	0	0
Character	11	12	13	14	15	16	17	18	19	20
State	1	0	1	0	1	0	1	2	4	1
Character	21	22	23	24	25	26	27	28	29	30
State	1	1	2	0	2	2	01	2	1	012
Character	31	32	33	34	35	36	37	38	39	40
State	012	2	01	23	0	0	1	2	2	01
Character	41	42	43	44	45	46	47	48	49	50
State	1	01	01	1	1	1	1	1	1	1
Character	51	52	53	54	55	56	57	58	59	60
State	1	1	1	0	2	0	0	2	2	1
Character	61	62	63	64	65	66	67	68	69	70
State	1	1	0	01	0	2	1	3	01	01
Character	71	72	73	74	75	76	77	78	79	80
State	1	01	2	3	0	0	1	01	1	0
Character	81	82	83	84	85	86	87	88	89	90
State	01	01	1	0	0	01	01	01	1	1
Character	91	92	93	94	95	96	97	98	99	100
State	1	01	1	2	2	2	2	3	1	01

## 6 CONCLUSION

Here, we reconstructed the hindlimb musculature of the Jurassic Piatnitzkysauridae clade. We find a great anatomical similarity within Piatnitzkysauridae with minor differences, such as the origin of *M*. *ambiens* and size of *M*. *caudofemoralis brevis*. The similarities with Aves were the division of the *M*. *iliofemoralis externus* and *M*. *iliotrochantericus caudalis*, as well as the broad depression of the *M*. *gastrocnemius pars medialis* origin on the cnemial crest of the tibia. Our results shed some light on palaeontological issues regarding megalosauroids and contribute to knowledge about the evolution of locomotor muscles in Theropoda.

## AUTHOR CONTRIBUTIONS

Mauro B. S. Lacerda: conceptualization (equal), investigation, methodology, visualization, formal analysis, writing—original draft, and writing—review and editing. Jonathas S. Bittencourt: conceptualization (equal), writing—original draft, and validation (equal). John R. Hutchinson: conceptualization (equal), investigation, writing—original draft, writing—review and editing, validation (equal), and supervision.

## Data Availability

Data for this study are available in the Figshare Repository: https://figshare.com/s/4cded3bafca1932a7c0b.

## APPENDIX A

### A.1

List of characters scored and mapped onto a simplified Reptilia phylogeny. For the original and updated version of the taxon‐character matrix refer to Hutchinson (2001a, 2001b, 2002) and Bishop et al. (2021). Brackets [] indicate osteological correlates of character states.

1. *M*. *iliotibialis* (IT), number of heads: one or two, weakly subdivided (0); three [three separate regions of muscle scarring] (1).
2. IT, origin: dorsolateral ilium, superficial to other iliac muscles [rugose dorsal rim of ilium] (0).
3. IT, insertion (common “triceps” extensor tendon): tibial tuberosity (0); cnemial crest (1); cranial and cnemial crests (2). Ordered.
4. Patella, ossified sesamoid in extensor tendon: absent (0); present [patellar sulcus presents on femur as proximal extension of intercondylar sulcus] (1).
5. *M*. *femorotibialis* (FMT), number of heads: one, weak subdivision (0); two [anterior and posterior intermuscular lines] (1); three [medial intermuscular line] (2). Ordered.
6. FMT, origin: proximal half of femoral shaft (0); bulk of femoral shaft [strong intermuscular lines] (1).
7. FMT, distal subdivision of lateral head: absent (0); present [anteromedial muscle scar on distal femoral shaft] (1).
8. FMT, insertion: with IT + AMB extensor tendon (0).
9. *M*. *ambiens* (AMB), number of heads: one (0); two (1).
10. AMB, origin(s): pubic tubercle; proximally adjacent to *M*. *pubotibialis*, PUT (if present) (0); anterior preacetabular cartilage and medial proximal pubis (1).
11. AMB, insertion: with IT + FMT extensor tendon (0); additional secondary tendon perforating extensor tendon, to origins of digital flexors (and GL) (1).
12. *M*. *iliofibularis* (ILFB), origin: dorsal postacetabular ilium, between IF and FTE [scarred region] (0).
13. ILFB, insertion: anterolateral proximal fibula [rugosity] (0); anterolateral proximal fibula [tubercle] (1); lateral proximal fibula [tubercle] (2); posterior proximal fibula [tubercle] (3). Ordered.
14. ILFB, secondary tendon: absent (0); present to GL aponeurosis (1).
15. ILFB, insertion induces fibular crest on tibia: absent (0); present (1).
16. ILFB, ansa: absent (0); present [tubercles on lateral femoral condyle near GL origin] (1).
17. *M*. *iliofemoralis* (IF), number of heads: one, IF (0); two, IFE and ITC [separate insertions evident] (1).
18. IF, origin: small, above acetabulum (0); large, expanded preacetabularly with ilium (1); divided into IFE and ITC portions (2).
19. IF (or IFE), insertion: posterolateral [internal trochanter] (0); anterolateral [greater trochanter] (1); posterolateral femoral shaft between FMT origins [flat shelf; proximal knob] (2); IFE on prominent trochanteric shelf or lateral ridge; ITC separate (3); IFE on reduced scar‐like trochanteric shelf; ITC separate (4).
20. IF (or ITC + IFE), insertion type: fleshy [flat surface or internal trochanter] (0); tendinous [bladelike trochanters or fibrous scars] (1).
21. IFE, origin: absent; not divided from ITC (0); above acetabulum on lateral surface of ilium (1); reduced to dorsolateral tubercle [processus supratrochantericus] (2).
22. ITC, origin: absent; not divided from IFE (0); anterior to IFE on lateral iliac surface (1); expanded into preacetabular concavity (2). Ordered.
23. ITC, insertion: absent; not divided from IFE (0); small distal knob‐like lesser trochanter (1); large blade‐like lesser trochanter (2); robust lesser trochanter; proximally positioned and closely appressed to greater trochanter (3); scar on cranial rim of proximal trochanteric crest [greater and lesser trochanters fused] (4). Ordered.
24. *M*. *puboischiofemoralis internus* (PIFI) 1 + 2, number of heads: one; PIFI1 + 2 (PIFI1 of Crocodylia or *M*. *iliofemoralis internus*, IFI of Neornithes) (0); two; PIFI1 and PIFI2 (weakly subdivided in Squamata) (1).
25. PIFI1 + 2, origin: anteromedial puboischiadic plate and epipubic cartilage (0); medial ilium and proximal ischium [puboischiadic plate reduced] (1); ventrolateral ilium [preacetabular “cuppedicus” fossa] (2); lateral ilium [preacetabular fossa reduced] (3); lateral pubic peduncle of ilium [preacetabular fossa “lost”] (4).
26. PIFI1 + 2, insertion: anteromedial femoral shaft [scar] (0); anteromedial proximal femur; with PIFI3 [lesser trochanter] (1); medial proximal femoral shaft [scar] (2).
27. PIFI3, number of heads: one; PIFI2 or PIFI3 (Crocodylia) [one insertion scar] (0); two; *Mm*. *iliotrochanterici cranialis* (ITCR) *et medius* (ITM) of Neornithes [two distinct scars] (1).
28. PIFI3 (PIFI2 of Crocodylia or ITCR+ITM of Neornithes), origin: anteromedial pubes and part of medial ilium [puboischiadic plate] (0); lumbar vertebrae [puboischiadic plate reduced] (1); ilium [no lumbar vertebrae; preacetabular “cuppedicus” fossa] (2); lateral preacetabular ilium [preacetabular fossa lost] (3).
29. PIFI3 (PIFI2 or ITCR+ITM), insertion: anterolateral proximal femur [scars] (0); accessory trochanter (1); anterior and lateral trochanteric crest [two scars] (2); lesser trochanter, with PIFI1 + 2 (3).
30. *M*. *puboischiotibialis* (PIT), number of heads; most superficial of *flexor cruris*: one, PIT1–3; weakly subdivided (0); two, PIT1 + 2 and PIT3 (PIT and FTI2 of Crocodylia) (1); none; absent (2).
31. PIT1 + 2, origin: anteroventral puboischiadic ligament and pubic tubercle; near PUT (0); anteroproximal ischium (PIT of Crocodylia) [reduced ligament; scar; PUT lost] (1); none; absent (2).
32. PIT3, origin: posterior end of puboischiadic ligament and pelvic symphyses (0); anterolateral ilium (FTI2 of Crocodylia); posteroventral to FTE, dorsal to CFB [ligament reduced] (1); none; absent or not separate from PIT1 + 2 (same origin) (2).
33. PIT1–3, insertion: medial proximal tibia; with or proximal to FTI1 (0); none; absent (1).
34. *M*. *flexor tibialis internus* (FTI), number of heads: two; FTI1 and FTI2 (0); three; FTI1 and FTI2a/b (FTI2a/b = superficial/deep = FTI4/3 of Crocodylia) (1); one; *M*. *flexor cruris medialis* (FCM of Neornithes; equivalent to FTI2b of FTI3 of Crocodylia); FTI1 and FTI4 absent (2).
35. FTI1, origin; posterodorsal to FTI2 and ISTR, near *M*. *ischiocaudalis* origin: ilioischiadic ligament or fascia on posterolateral side of distal ischium (0); last sacral and proximal caudal vertebrae, fascia (1); none; absent (2).
36. FTI1, insertion: posteromedial proximal tibia; with or distal to PIT (0); Unites distally with FTI2, to GM origin at posteromedial proximal tibia (1); none; absent (2).
37. FTI2, origin: anterior end of ilioischiadic ligament/fascia, near or on ischial tuberosity and distal ischium (0); scar on ischial tuberosity, and ilioischiadic fascia (FTI3 + 4 of Crocodylia) (1); proximal dorsal process of ischium, and ilioischiadic fascia [ischial tuberosity expanded as process] (2); posterolateral distal ischium, and ilioischiadic membrane (FCM of Neornithes) [proximal dorsal process lost] (3).
38. FTI2; insertion of tendon of superficial part (FTI2a or FTI4): splits distally into two tendons, inserting on medial and lateral proximal tibia around GM origin (0); unites distally with FTE and deeper part (FTI2b; FTI3 of Crocodylia) (1); none; muscle absent or not separate (2).
39. FTI; insertion of deep part (FTI2b or FTI3 or FCM): posterior proximal tibia; distal to PUT (0); not distinct from superficial part (FTI2a), unites distally with FTI1 (1); unites distally with FTE (= *M*. *flexor cruris lateralis pars pelvica*; FCLP of Neornithes) tendon (and FTI4, if present) (2).
40. FTI2 (FTI3 + 4 of Crocodylia); secondary tendon to GL: absent (0); present (1).
41. *M*. *flexor tibialis externus* (FTE) (= FCLP); origin, most posterodorsal of *flexor cruris*: ilioischiadic ligament/fascia around posteroventral ilium and posterodorsal ischium (0); posterolateral surface of ilium [muscle scar] (1).
42. FTE; insertion, shared tendon inserts between heads of *Mm*. *gastrocnemii*: tendon splits distally; medial tendon to posteromedial proximal tibia near GM; lateral tendon to posterolateral proximal tibia near GL and digital flexors, contributing to plantar aponeurosis (0); medial proximal tibia (FCLP of Neornithes), with FCM, between GM + GIM (1).
43. FCL *pars accessoria* head (FCLA): absent (0); present, originating from FCLP raphe and inserting in popliteal fossa of femur (1).
44. *M*. *pubotibialis* (PUT): present (0); absent (1).
45. PUT, origin; most anterior of *flexor cruris* and distally adjacent to AMB: pubes, proximal to or onto pubic tubercle and puboischiadic ligament (0); none; absent (1).
46. PUT, insertion: posterolateral proximal tibia; between GM and GL; proximal to other *flexor cruris* parts (0); none; absent (1).
47. *M*. *adductor femoris* (ADD), number of heads: one (0); two; ADD1 and ADD 2 of Crocodylia, or *Mm*. *puboischiofemorales medialis* (PIFM) *et lateralis* (PIFL) of Neornithes [two insertion scars] (1).
48. ADD, origin: puboischiadic ligament, anterior and deep to *flexor cruris* (0); anterior and posterior edges of ischium, separated by PIFE3 [scars; PIFE3 present] (1); anterolateral edge of ischium, puboischiadic membrane, and posterolateral pubis [PIFE3 absent] (2).
49. ADD, insertion: posterior distal femoral shaft, one scar on adductor ridge (0); posterior distal femoral shaft, medial and lateral scars (1); adductor ridge or crista supra condylaris medialis connecting to medial condyle (2).
50. *M*. *puboischiofemoralis externus* (PIFE), number of heads: one; PIFE1–3; weakly subdivided [nearly continuous puboischiadic plate] (0); three; PIFE1–3 [expanded and separated pubic and ischial aprons; obturator process] (1); two; *Mm*. *obturatorii lateralis* (OL) *et medialis* (OM) of Neornithes, equivalent to PIFE1 and PIFE2; PIFE3 lost [obturator process lost] (2).
51. PIFE1, origin: thyroid fenestra and puboischiadic plate (0); anteromedial surface of pubic apron and epipubic cartilage (1); proximal lateral pubis; OL of Neornithes [pubic apron lost] (2).
52. PIFE2, origin: anterior to PIFE1; not separated (0); posterior surface of pubic apron (1); medial puboischiadic membrane; OM of Neornithes [pubic apron lost] (2).
53. PIFE3, origin: posterior to PIFE1 + 2; not separated (0); lateral ischium, remnant of puboischiadic plate [or obturator process] (1); absent [obturator process lost] (2).
54. PIFE1 + 2, pubic retroversion: pubic shaft oriented anteriorly (0); pubic shaft near vertical (1); pubic shaft oriented posteriorly (2); ilia, pubes, and ischial lie nearly parallel (3). Ordered.
55. PIFE insertion: anterolateral internal trochanter, posterior ridge, and intertrochanteric fossa (0); tip of trochanter minor and intertrochanteric fossa (1); posterolateral proximal femur [greater trochanter] (2); lateral proximal femur [greater trochanter rotated laterally] (3); groove and pit on posterolateral side of trochanteric crest [greater and lesser trochanters fused] (4).
56. PIFE2 (OM of Neornithes), obturator tuberosity of ischium for tendon: absent (0); present (1).
57. *M*. *ischiotrochantericus* (ISTR) (*M*. *ischiofemoralis*; ISF of Neornithes), origin: medial surface of posterior ischium [ischial symphysis] (0); lateral surface of posterior ischium and ilioischiadic membrane [ischial symphysis lost] (1).
58. ISTR, insertion: posterolateral proximal femur [scar] (0); groove proximal to trochanteric shelf (1); lateral proximal femur near trochanteric shelf [reduced trochanteric shelf] (2); proximal to large posterior trochanter (3); posterolateral trochanteric crest [scar; reduced posterior trochanter] (4).
59. *M*. *caudofemoralis brevis* (CFB) (*M*. *caudofemoralis pars pelvica*; CFP of Neornithes), origin: proximal caudal vertebrae and fascia (0); proximal caudals, lasta sacrals, and medioventral ilium [small shelf] (1); posteroventral ilium [brevis fossa] (2); ventrolateral ilium [brevis fossa reduced onto lateral ilium] (3); posterolateral ilium [brevis fossa lost] (4).
60. CFB, insertion: weakly differentiated from CFL; near internal trochanter (0); proximal and lateral to CFL, if separate [fourth trochanter or scar present] (1); posterolateral proximal femur [greater trochanter] (2).
61. *M*. *caudofemoralis longus* (CFL) (*M*. *caudofemoralis pars caudalis*; CFC of Neornithes), origin: ventral centra and transverse processes of caudal vertebrae [no transition zone in caudals] (0); restricted to proximal half of tail [“transition zone”] (1); restricted to proximal caudals [tail shortened to 15–30 vertebrae] (2); anteroventral pygostyle (3); none; absent (4). Ordered.
62. CFL, insertion: posterior femoral shaft and internal trochanter (0); prominent fourth trochanter and medial pit (1); small fourth trochanter (2); fourth trochanter reduced to a scar (3); none; absent (4).
63. CFL, secondary tendon to lateral knee region (and GL): absent [loss of pendant trochanter or CFL absent] (0); from distal to CFL (1); from crest‐like fourth trochanter (2); from tip of pendant fourth trochanter (3).
64. *Mm*. *gastrocnemii*, number of heads: two; *Mm*. *gastrocnemii lateralis* (GL) *et medialis* (GM; “*femorotibial gastrocnemius*” of Lepidosauria) (0); three; GM divided into GM and *M*. *gastrocnemius pars intermedia* (GIM; Neornithes) or GL divided into superficial and deep heads of “*femoral gastrocnemius*” (Lepidosauria) (1).
65. GL, origin: posterolateral distal femur near lateral condyle [tubercle or scar] (0).
66. GL, insertion: plantar aponeurosis to metatarsal V and tarsal, then to digits 2–4 (0); plantar aponeurosis to metatarsal V, process on distal tarsal 4, and calcaneal tuber, then to digits 2–4 [calcaneal tuber present] (1); reduced plantar aponeurosis to posterior surface of metatarsals II–V [scars; calcaneal tuber and distal tarsal 4 process lost] (2); forms lateral part of “Achilles tendon” onto small flat hypotarsus, then vestigial plantar aponeurosis to posterior surface of tarsometatarsus (3); forms lateral part of “Achilles tendon” onto large grooved hypotarsus, then vestigial plantar aponeurosis to posterior surface of tarsometatarsus (4).
67. GM, origin; medial to TA: medial proximal tibia (0); medial side of lateral cnemial crest (1).
68. GM, insertion: plantar aponeurosis to metatarsal V, calcaneum, then to digit 5 (0); plantar aponeurosis to metatarsal V, calcaneal tuber, then to digit 5 [calcaneal tuber present] (1); plantar aponeurosis to metatarsal V and calcaneal tuber [digit 5 phalanges lost] (2); plantar aponeurosis to metatarsal V [calcaneal tuber lost] (3); forms medial part of “Achilles tendon” onto small flat hypotarsus, then vestigial plantar aponeurosis to posterior surface of tarsometatarsus [metatarsal V lost] (4); forms medial part of “Achilles tendon” onto large grooved hypotarsus, then vestigial plantar aponeurosis to caudal surface of tarsometatarsus (5).
69. GIM, origin: absent; not divided (0); posterior side of distal femur, near medial femoral condyle; at distal end of PIFM, PIFL, and FCLA insertions (1).
70. GIM, insertion: absent; not divided (0); joins GM, then forms posterior part of “Achilles tendon” onto hypotarsus and posterior tarsometatarsus (1).
71. GM and GL, relative size: GL larger than GM [no large cnemial crest] (0); GM larger than GL (including GIM, if present) [expanded cnemial crest] (1).
72. *M*. *extensor digitorum longus* (EDL), origin: anteromedial proximal tibia; distal to tibial tuberosity and deep to TA origin (0); anterior proximal tibia; distal, medial and deep to TA origin (1). Modified after Hattori & Tsuihiji, 2021.
73. EDL, insertion: anterolateral proximal metatarsals I–IV; especially metatarsal I (0); dorsal surfaces of phalanges, digits 2–4 [through extensor sulci to scars and pits] (1). Modified after Hattori & Tsuihiji, 2021.
74. EDL, extensor canal on cranial side of distal tibiotarsus: absent (0); present; shallow groove (1); present; deep groove enclosed anteriorly by an ossified supratendinal bridge (2). Ordered.
75. TA, number of heads: one (0); two (femoral and tibial heads of *M*. *tibialis cranialis* of Neornithes; TC) [fossa on anterolateral distal femur for femoral origin] (1).
76. TA, origin: anterior surface of distal lateral femoral condyle; superficial to EDL origin (0); femoral fossa and anterior surfaces of cnemial crests; proximal and superficial to EDL origin [fossa on femur and two cnemial crests presents]. Modified after Hattori & Tsuihiji, 2021.
77. TA, insertion: anterior surfaces of proximal metatarsals II–IV (0); tubercles on anterior proximal metatarsals II–IV [metatarsal I shifted distally] (1); *tuberositas m*. *tibialis cranialis*, on cranial proximal metatarsal II (2). Modified after Hattori & Tsuihiji, 2021.
78. *M*. *extensor hallucis longus* (EHL), origin: anterolateral distalmost fibula (0); anteromedial proximal tarsometatarsus [distal fibular shaft lost] (1).
79. EHL, insertion: anterior surfaces of digit 1 phalanges [hallux not retroverted] (0); posterior surfaces of digit 1 phalanges [hallux retroverted] (1).
80. *M*. *extensor digitorum brevis* (EDB): present (0); absent; presumably fused to distal EDL (1).
81. EDB, origin: anterior surfaces of proximal tarsals (0); absent (1).
82. EDB, insertion: dorsal surfaces of distal phalanges (0); absent (1).
83. *M*. *fibularis longus* (FL), origin: lateral fibular shaft; distal to IFLB; between FB and FDL (0); lateral side of lateral femoral condyle (1); lateral proximal fibular shaft and nearby cnemial crests [distal fibular shaft lost]; proximal to ILFB (2).
84. FL, insertion: lateral side of metatarsal V; distal to FB; and slight tendon to dorsal surfaces of digit V phalanges (0); lateral side of metatarsal V; distal to FB; and calcaneal tuber and slight tendon to dorsal surfaces of digit 5 phalanges [calcaneal tuber present] (1); lateral side of metatarsal V, calcaneal tuber, and flexor tendon [digit 5 phalanges lost] (2); lateral side of metatarsal V and flexor tendon [calcaneal tuber lost] (3); tibial cartilage and through *sulcus m*. *fibularis longi* on tarsometatarsus o tendon of *M*. *flexor perforates digitorium III* [metatarsal V lost] (4).
85. *M*. *fibularis brevis* (FB), origin: anterolateral distal fibula (and tibia); anterolateral and distal to FL (0); anterolateral distal tibial shaft [distal fibular shaft lost] (1).
86. FB, insertion: anterolateral side of metatarsal V (and IV); proximal to FL (0); anterolateral proximal metatarsal IV [metatarsal V lost; *Tuberculum m*. *fibularis brevis* present] (1).
